# An Improved Genghis Khan Shark Optimization Algorithm for Solving Optimization Problems

**DOI:** 10.3390/biomimetics11040270

**Published:** 2026-04-14

**Authors:** Yanjiao Wang, Jiaqi Wang

**Affiliations:** School of Electrical Engineering, Northeast Electric Power University, 169 Changchun Road, Jilin 132012, China; 20132485@neepu.edu.cn

**Keywords:** Genghis Khan shark optimizer, population partitioning method, opposition-based learning, novel diversity assessment mechanism, parameter adaptation strategy

## Abstract

As an innovative metaheuristic algorithm, Genghis Khan Shark Optimization (GKSO) faces challenges, including a tendency towards local optima and poor convergence speed and accuracy. To mitigate these limitations, an improved Genghis Khan shark optimizer (IGKSO) is proposed in this paper. A population partitioning method based on cosine similarity and fitness is introduced, where individuals are strategically assigned to different evolutionary phases: Disadvantaged populations are responsible for the foraging stage. By contrast, advantaged populations dominate the moving stage. In the moving stage, the base vector is randomly selected from multiple candidates, which ensures the evolutionary direction of the population while maintaining its diversity. An adaptive step-size mechanism is introduced to avoid boundary overflow problems. A subspace method is employed to prevent diversity loss during foraging. Additionally, in the hunting stage, a novel opposition-based learning strategy is proposed to moderate the tendency of converging to suboptimal solutions. Furthermore, during the self-protection phase, a criterion for assessing the diversity of the whole population is employed to monitor and supplement diversity in real time. The results of the CEC2017 and CEC2019 benchmark test sets reveal that IGKSO exhibits substantial advantages over the GKSO algorithm and eight other high-performance algorithms in terms of convergence speed and accuracy.

## 1. Introduction

Many engineering and scientific problems prevalent in the real world [1,2,3,4,5,6] can be abstracted as high-dimensional [7], nonlinear [8], and multimodal optimization challenges [9]. Traditional mathematical programming methods often face high computational complexity [10] and are prone to becoming trapped in local optima when solving such problems. Metaheuristic algorithms, characterized by their simple structure, ease of implementation, and low dependence on problem models, have been widely adopted for tackling complex optimization problems.

In recent years, researchers have continuously drawn inspiration from natural and social phenomena, leading to a series of novel metaheuristic algorithms. For instance, Jia et al. proposed the remora optimization algorithm (ROA) in 2021, inspired by the parasitic behavior of remoras. ROA has been applied to solve engineering problems such as I-beam design, welded beam design, pressure vessel design, three-bar truss design, and rolling bearing problems [11]. Xie et al. [12] proposed the tuna swarm optimization algorithm (TSO) in 2021, inspired by the cooperative foraging behavior of tuna schools. TSO has been widely applied in fields such as power system dispatch, wind farm layout, reservoir scheduling, workshop layout, logistics site selection, and vehicle routing problems. In 2022, Srivastava and Das proposed the bottlenose dolphin optimizer (BDO) for signal decomposition and parameter tuning problems. Inspired by the social behaviors of bottlenose dolphins—such as cooperative hunting, information transmission, and spatial positioning—BDO constructs a mathematical model for solving complex optimization problems [13]. Zhong et al. introduced the beluga whale optimization (BWO) algorithm, which simulates three core behaviors of beluga whales: swimming exploration, cooperative predation, and whale falling. BWO has been applied to power system economic dispatch, job shop scheduling, and machine learning model training [14]. Wang et al. [15] inspired by the survival strategies of rabbits in nature, proposed the artificial rabbits optimization (ARO) algorithm in 2022, which simulates behaviors such as detour foraging and random hiding to find optimal solutions. ARO has been successfully applied in function optimization, engineering design optimization (e.g., pressure vessel design), and wireless sensor network coverage optimization. Braik et al. [16] proposed the white shark optimizer (WSO) in 2022, inspired by the predatory behavior of great white sharks. WSO simulates their acute hearing, smell, and cooperative hunting strategies to solve practical engineering problems such as microgrid economic operation and motor fault diagnosis. Wang et al. proposed an improved Archimedes optimization (IAOA) algorithm, which introduces a simple mechanism to correct for poorer individuals and enhance the algorithm’s convergence speed and accuracy [17]. During the same year, Jia et al. proposed the crayfish optimization algorithm (COA), inspired by the foraging, summer vacation, and competitive behaviors of crayfish. COA is applied in 2D/3D path planning for UAVs, robots, and AGVs to optimize path length, smoothness, and obstacle avoidance, as well as in solar cell defect detection to optimize the hyperparameters of deep learning models [18]. In 2023, Hu et al. proposed the Genghis Khan shark optimizer (GKSO) for solving engineering problems such as gas transmission compressor design, multi-disk clutch brake design, stepped cone pulley problems, and welded beam design. By simulating four typical behaviors—hunting, moving, foraging, and self-protection—GKSO achieves a good balance between global exploration and local exploitation [19]. In 2024, Mohammad et al. introduced a multi-trial vector-based sine–cosine algorithm (MTV-SCA) [20]. MTV-SCA employs a multi-trial vector approach with three control parameters to achieve specific search objectives and has been applied to six non-convex engineering problems, including gas transmission compressor design and speed reducer design [20]. Ye et al. proposed a multi-strategy improved dung beetle optimizer (MDBO) in 2024 for three representative engineering applications: tension spring design, speed reducer design, and welded beam design [21]. In 2024, Abdulrab et al. developed a novel hybrid optimization algorithm called the transient triangular Harris Hawks optimizer (TTHHO) for solving wireless mesh network (WMN) optimization problems [22].

Among numerous novel metaheuristic algorithms, the Genghis Khan shark optimizer (GKSO) stands out due to its excellent search efficiency and superior balance between exploration and exploitation, achieving significantly better optimization performance than fish-based algorithms such as COA, BDO, and BWO. Since its inception, it has been applied to complex engineering optimization problems. For example, in 2024, Abdel-Salam et al. proposed an improved Genghis Khan optimizer (IGKSO1) for global optimization and feature selection problems, incorporating an enhanced solution quality (ESQ) strategy and a quasi-oppositional-based learning (QOBL) technique to improve solution quality [23]. Mostafa et al. proposed an enhanced Genghis Khan shark optimizer for multi-UAV network problems [24]. Zhou et al. introduced an improved Genghis Khan shark optimizer for diagnosing knee meniscus tears [25]. These studies have significantly enriched the theoretical framework and application scenarios of GKSO, laying a solid foundation for subsequent research.

However, these investigations have also revealed certain limitations of the GKSO algorithm. When dealing with high-dimensional and multimodal optimization problems, GKSO still exhibits a tendency toward premature convergence and insufficient population diversity. In view of this, this paper proposes an improved Genghis Khan shark optimizer (IGKSO) based on an in-depth analysis of GKSO’s search characteristics. Specifically, the main contributions of this paper are as follows:

(1) The hunting phase incorporates an opposition-based learning strategy to maintain population diversity and escape local optima more effectively.

(2) A dual-selection method based on fitness and cosine similarity is employed to partition the population, guiding the evolutionary process in a targeted manner. In the moving phase, the elite subpopulation adopts a novel basis vector selection scheme, randomly selecting one from two individuals generated by simulated binary crossover (SBX), the best individual, and the current individual. This approach ensures both the evolutionary direction and population diversity. Furthermore, compared with fixed step sizes, the proposed adaptive step size mechanism not only normalizes fitness but also fully considers the relationship between step size and population evolution state, constraining the step size within [0, 1] and thereby significantly reducing the likelihood of boundary violations. In the foraging phase, the inferior subpopulation updates individual positions using subspaces, avoiding excessive algorithm convergence, thus reducing the probability of diversity loss, and the use of adaptive parameters achieves a balance between exploration and exploitation.

(3) In the self-protection phase, population diversity is quantified in real time through the hypervolume ratio between the population space and the search space, enabling dynamic monitoring of the population’s distribution state and evolutionary progress. Additionally, the algorithm introduces multiple adaptive parameters that work in coordination to regulate the individual step sizes and search directions, collectively achieving an effective balance between global exploration and local exploitation. Experimental results on the CEC2017 and CEC2019 benchmark suites demonstrate that the proposed algorithm exhibits superior overall performance in terms of both convergence speed and convergence accuracy compared to eight other representative algorithms.

The subsequent sections of this paper are arranged as follows: Section 2 presents the fundamental principles and process of the original GKSO algorithm. Section 3 critically analyzes its limitations and systematically proposes the IGKSO algorithm with core innovations. Section 4 conducts comprehensive experimental comparisons between IGKSO, the original GKSO, and other excellent optimization algorithms on the CEC2017 and CEC2019 benchmark suites to rigorously evaluate its efficacy. Finally, Section 5 summarizes the key contributions of this paper.

## 2. Genghis Khan Shark Optimizer

**Figure 1:**
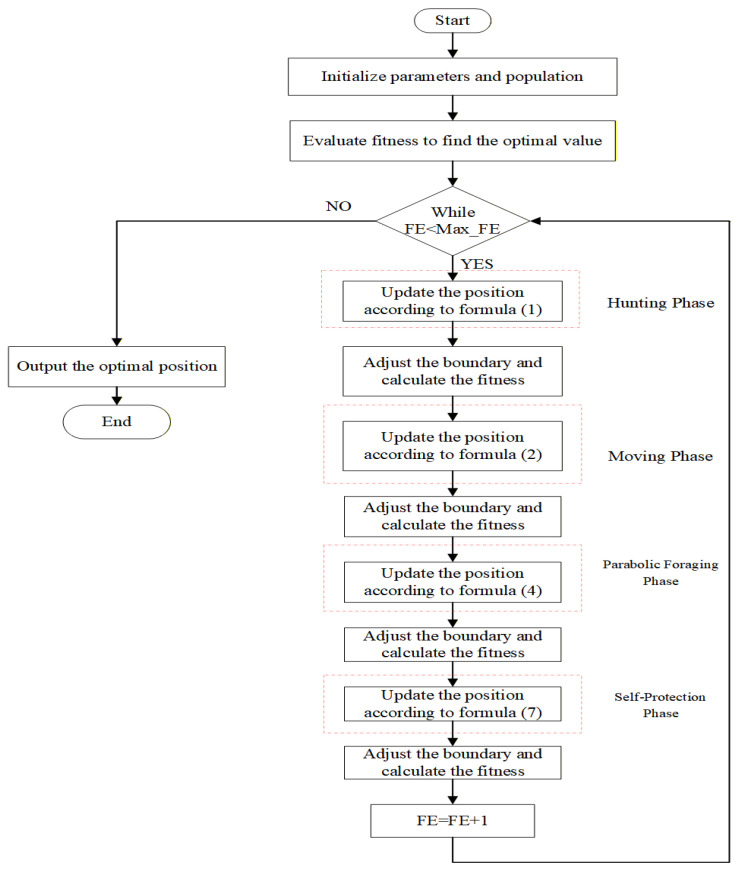
Flowchart of GKSO.

The GKS, a marine predator renowned for its robust physique and remarkable intelligence, exhibits sophisticated cooperative hunting strategies against prey. And when encountering predators that threaten their safety or compete for prey, the shark activates a rapid color-changing mechanism in which its tail and body coloration lightens to deter threats, enabling swift escape. Hu et al. (2023) [19] proposed the Genghis Khan shark optimizer (GKSO), inspired by these distinctive behaviors. In the GKSO framework, the individual represents the positional information of a Genghis Khan shark, and the fitness value quantifies the quality of each shark’s position, which comprises hunting, moving, foraging, and self-protection phases. The pseudocode and flowchart of GKSO are presented in Algorithm 1 and Figure 1.
**Algorithm 1 The Pseudo-Code of GKSO****Input:** Population: *N*; Dimension of Optimization Problem: *D*; Minimum Boundary Value of Optimization Problem: *lb*; Maximum Boundary Value of Optimization Problem: *ub*; Olfactory Intensity: *s*; Step Size: *p*; Adaptive Coefficient: *ρ*; Maximum Iterations: *Max_iter*.
**Output:** The optimal solution and its fitness value1. Variable initialization (*N*, *D*, *lb*, *ub*, *s*, *p*, *ρ*, *Max_iter*)2. Implement X=lb+rand×(ub−lb) to initialize population *X* containing *N* individuals; %rand~U(0,1)3. Evaluate fitness *fit_i_* for each individual $X_{i}$, identify *xbest* and *best_fit*4. **For** *t* = 1 **to** *Max_iter* **do**5.   $X_{i}^{t+1}$ ← $X_{i}^{t}$ as described in Equation (1).6.   Implement boundary handling mechanisms. 7.   Evaluate $X_{i}$ and replace the original individual if the new one is better. 8.   $X_{i}^{t+1}$ ← $X_{i}^{t}$ as described in Equation (2).9.   Implement boundary handling mechanisms.10.  Evaluate $X_{i}$ and replace the original individual if the new one is better. 11.  $X_{i}^{t+1}$ ← $X_{i}^{t}$ as described in Equation (4).12.  Implement boundary handling mechanisms.13.  Evaluate $X_{i}$ and replace the original individual if the new one is better. 14.  $X_{i}^{t+1}$ ← $X_{i}^{t}$ as described in Equation (7).15.  Implement boundary handling mechanisms. 16.  Evaluate $X_{i}$ and replace the original individual if the new one is better.17.**end For**18.**output** *xbest* and *best_fit*

### 2.1. Hunting Phase

The Genghis Khan shark (GKS) is a powerful predator in freshwater river ecosystems. It typically patrols near the riverbed to ensure that no dominant predators occupy its territory. This behavior not only safeguards the shark’s security but also expands the search space to satisfy convergence to the position of optimal prey.

The GKSO algorithm implements a hunting operation defined by Equation (1) to simulate this biological strategy. Each individual updates its position by identifying a stochastic location within the search space as an “optimal hunting location”.

(1) $$X_{i}^{j}(t+1)=X_{i}^{j}(t)+\frac{lb_{j}+r_{1}\times \left(ub_{j}-lb_{j}\right)}{t}$$

Here, i=1,…,N, *N* denotes population size; j=1,…,D, *D* represents problem dimension; t=1,…,Max_iter, *t* and Max_iter are the current iteration and the maximum iteration; $X_{i}$ indicates the current individual of the population; ub and lb represent the upper bound and lower bounds of the search space, respectively; and *r*_1_ represents a random variable limited to the range of [0, 1].

### 2.2. Moving Phase

The template Genghis Khan sharks primarily depend on their acute olfactory sense to acquire superior-quality prey, progressively approaching optimal prey locations. The algorithm implements the position updating through the movement operation defined in Equation (2), which simulates the aforementioned biological process. As can be seen from Equation (2), when  i=1, the new individual is determined solely by the difference vector between the current individual and the best individual. In contrast, when i≠1, the new individual depends not only on the aforementioned difference vector but also on its preceding individual.

(2) $$X_{i}^{j}(t+1)=\left\{\begin{array}{ll} s\times \left(X_{best}^{j}(t)-X_{i}^{j}(t)\right) & i=1\\ \frac{s\times \left(X_{best}^{j}(t)-X_{i}^{j}(t)\right)+X_{i-1}^{j}(t)}{2} & i\neq 1 \end{array}\right.$$

Here, $X_{best}$ denotes the optimal hunting position; *s* represents olfactory intensity toward the optimal prey during the moving phase, which is determined by the odor concentration released by the prey as formulated in Equation (3).

(3) $$s=mI^{r}$$

Here, the parameter *I* represents each individual’s objective function value; the parameter *m* is a constant typically assigned 1.5 for optimal performance; and *r* represents a random variable limited to the range of [0, 1], reflecting the current individual’s absorption rate of prey odor. When *r* = 0, it indicates that the odor released by the prey cannot be detected by the GKS at all. In contrast, when *r* = 1, the odor is entirely assimilated by the shark, which may cause the approach to either swiftly converge to the global optimum or get trapped in local optima.

### 2.3. Parabolic Foraging Phase

The Genghis Khan Shark (GKS) exhibits a characteristic parabolic predation strategy during foraging: initially approaching optimal prey positions gradually, then rapidly accelerating toward the target using speed advantages, and ultimately using its broad oral cavity to deliver a lethal strike to the prey’s head. This entire process follows a parabolic trajectory. To computationally model this behavior, the GKSO algorithm incorporates a parabolic foraging phase, as formalized in Equation (4).

(4) $$X_{i}^{j}(t+1)=X_{best}^{j}(t)+r_{2}\times (X_{best}^{j}(t)-X_{i}^{j}(t))+\lambda \times p^{2}\times (X_{best}^{j}(t)-X_{i}^{j}(t))$$

Here, *r*_2_ represents a random variable limited to the range of [0, 1]; λ randomly takes values from {−1, +1}; *p* controls the movement step size of GKS according to Equation (5); and |ω(t+1)| represents the weighting coefficient at time *t* + 1, which is calculated by Equation (6).

(5) $$p=2\times \left\{1-{\left(\frac{t}{Max\_iter}\right)}^{\frac{1}{4}}+|\omega (t+1)|\times \left[{\left(\frac{t}{Max\_iter}\right)}^{\frac{1}{4}}-{\left(\frac{t}{Max\_iter}\right)}^{3}\right]\right\}$$

(6) $$\left|\omega (t+1)|=1-2\omega^{4}(t\right)$$

where ω(0)=1.

### 2.4. Self-Protection Phase

Genghis Khan sharks (GKS) frequently encounter predators threatening their safety or competing for prey resources during the foraging phase. In such scenarios, GKS quickly flee and exhibit a distinctive defensive response that lightens their tail and body color. The GKSO algorithm imitates this survival strategy through a mathematically formalized self-protection mechanism, as specified in Equation (7).

(7) $$X_{i}^{j}(t+1)=\left\{\begin{array}{cc} X_{i}^{j}(t)+k_{1}(a_{1}X_{best}^{j}(t)-a_{2}X_{k}^{j}(t))+k_{2}\rho (a_{3}(X2^{j}(t)-X1^{j}(t))) & \\ \;+a_{2}(X_{u1}^{j}(t)-X_{u2}^{j}(t))/2 & if\;a_{1}<0.5\\ X_{best}^{j}(t)+k_{1}(a_{1}X_{best}^{j}(t)-a_{2}X_{k}^{j}(t))+k_{2}\rho (a_{3}(X2^{j}(t)-X1^{j}(t))) & \\ \;+a_{2}(X_{u1}^{j}(t)-X_{u2}^{j}(t))/2 & else \end{array}\right.$$

Here $k_{1}$ represents a random variable uniformly distributed in the interval [−1, 1]; $k_{2}$ follows a standard normal distribution; $a_{1}$, $a_{2}$ and $a_{3}$ are stochastic coefficients calculated by Equations (8)–(10), respectively; X1 and X2 denote two distinct individuals randomly initialized within the search domain; $X_{u1}$ and $X_{u2}$ represent two randomly chosen individuals from the current population; $X_{k}(t)$ represent a newly generated individual computed according to Equation (11); and ρ is an adaptive coefficient determined by Equation (12).

(8) $$a_{1}=l_{1}\times 2\times rand+(1-l_{1})$$

(9) $$a_{2}=l_{1}\times rand+(1-l_{1})$$

(10) $$a_{3}=l_{1}\times rand+(1-l_{1})$$

Here $l_{1}$ randomly takes values from {0, 1}.

(11) $$X_{k}^{j}(t)=l_{2}\times \left(X_{p}^{j}(t)-X_{r}^{j}(t)\right)+X_{r}^{j}(t)$$

$X_{r}$ represents a randomly initialized individual; $X_{p}$ denotes an individual randomly chosen from the population; and $l_{2}$ is identical to $l_{1}$.

(12) ρ=α×(2×rand−1)

Here, *α* is computed according to Equation (13).

(13) $$\alpha =\left|\beta \times \sin(\frac{3\pi }{2}+\sin(\frac{3\pi }{2}\beta ))\right|$$

Here, *β* is computed according to Equation (14).

(14) $$\beta =\beta_{\min}+(\beta_{\max}-\beta_{\min})\times {\left(1-{\left(\frac{t}{Max\_iter}\right)}^{3}\right)}^{2}$$

Here, *β_max_* and *β_min_* take values of 0.2 and 1.2, respectively.

## 3. Improved Genghis Khan Shark Optimizer

**Figure 2:**
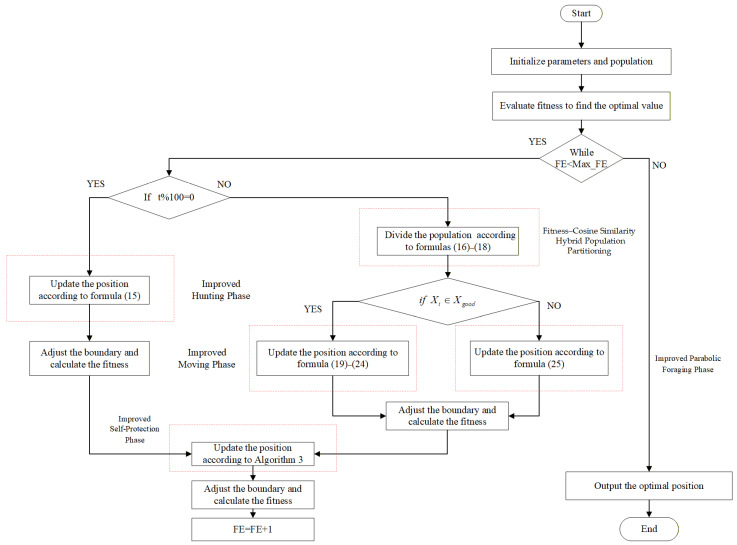
Flowchart of IGKSO.

To further enhance the precision of the GKSO algorithm, reduce its susceptibility to local optima, and maintain population diversity, IGKSO is proposed in this section. Its flowchart and pseudocode are represented in Algorithm 2 and Figure 2.
**Algorithm 2 The Pseudo-Code of IGKSO****Input:** Population: *N*; Dimension of Optimization Problem: *D*; Minimum Boundary Value of Optimization Problem: *lb*; Maximum Boundary Value of Optimization Problem: *ub*; Step Size: *p*_1_; Adaptive Coefficient: *ρ*; Maximum Iterations: *Max_iter;* Upper and Lower Boundary Value of Population: *u_x_* and *l_x_*, respectively; Number of Superior Sub-population: *N*_1_; Adaptive Step Size: *s*_1_.
**Output:** The optimal solution and its fitness value1. Variable initialization (*N*, *D*, *u_x_*, *l_x_*, *s*_1_, *p*_1_, *ρ*, *Max_iter*, *lb*, *ub*)2. Implement X=lb+rand×(ub−lb) to initialize population *X* containing *N* individuals; %rand~U(0,1)3. Evaluate fitness *fit_i_* for each individual $X_{i}$, identify *xbest* and *best_fit*4. **For** *FE* = 1 **to** *max_FE* **do**5.   **If** *t*%100 == 0 **do**6.      $X_{i}^{t+1}\leftarrow X_{i}^{t}$ as described in Equation (15).7.   **else**8.      **If** $\;X_{i}^{t}\in X_{good}$ **do**9.        $X_{i}^{t+1}\leftarrow X_{i}^{t}$ as described in Equation (19).10.     **else**11.       $X_{i}^{t+1}\leftarrow X_{i}^{t}$ as described in Equation (25).12.     **end If**13.  **end If**14.  Implement boundary handling mechanisms.15.  Evaluate *X_i_* and replace the original individual if the new one is better. 16.  $X_{i}^{t+1}\leftarrow X_{i}^{t}$ as described in Algorithm 317.  Implement boundary handling mechanisms.18.  Evaluate *X_i_* and replace the original individual if the new one is better.19.**end For**20.**output** *xbest* and *best_fit*

### 3.1. Improved Hunting Phase

Analysis of Equation (1) in Section 2.1 reveals that individual updates in the original GKSO hunting phase are implemented as “current position + decaying random perturbation” to maintain population diversity. However, this mechanism exhibits two critical limitations: In the early stage, excessive random perturbations induce disordered dispersion of individuals across the solution space, which disrupts fitness-driven directional evolution. Eventually, the problem of out-of-boundary occurs, which leads to futile search and waste of computation. Similarly, in later stages, faced with severe population diversity loss and high interindividual similarity, minor stochastic perturbations are ineffective in supplementing diversity and escaping from local optima, which results in a computational burden.

The analysis demonstrates that position updates via random perturbations have an extremely low probability of generating superior solutions compared with parent individuals, leading to inefficient resource utilization and ineffective diversity maintenance. Numerous experiments confirm that opposition-based learning demonstrates substantially better performance and achieves a higher probability of solution improvement and greater diversity preservation compared with random perturbations. Based on the above analysis, an enhanced hunting phase is proposed in this paper. The details are as follows: The execution frequency is activated every *k* generation (typically *k* = 100, though adjustable for specific problems), with the specific formula given in Equation (15).

(15) $$X_{i}(t+1)=u_{x}+l_{x}-X_{i}(t)$$

Here, $X_{i}(t)$ denotes current individual of the whole population; $u_{x}$ and $l_{x}$ indicate the upper and lower bounds of the whole population.

### 3.2. Improved Moving and Foraging Phase

In addition to the hunting phase, the GKSO algorithm primarily consists of the following three phases for individual updates: the moving phase, the foraging phase, and the self–protection phase. The roles of each phase are as follows: In the moving phase, individuals within the population continuously approach the optimal individual, accelerating algorithm convergence; in the foraging phase, each individual performs fine-grained exploitation near the global optimal individual; and the self-protection phase is mainly responsible for balancing exploration and exploitation. It is well known that different evolutionary phases have different requirements for algorithm performance. In the early iteration stage, individuals are relatively dispersed, with low similarity and good population diversity. At this point, it is desirable to have a larger number of superior individuals rapidly converge to the region of the optimal individual, while a smaller number of deviant individuals maintain population diversity. As evolution progresses, individuals gradually cluster together, becoming increasingly similar, and population diversity deteriorates. At this stage, it is desirable to have a small number of superior individuals perform a fine-grained search, while a larger number of deviant individuals supplement diversity to potentially escape local optima. However, in the GKSO algorithm, all individuals sequentially go through the moving phase, foraging phase, and self-protection phase without targeted evolution based on the evolutionary process, which significantly affects the algorithm’s search efficiency.

To enhance the algorithm’s convergence efficiency effectively, this section divides the population into superior and inferior subpopulations. The superior population relies on the moving phase for individual renewal, primarily responsible for achieving faster convergence, whereas the inferior subpopulation mainly relies on the foraging phase for individual renewal, primarily responsible for maintaining population diversity. The entire population then depends on the self-protection phase to maintain a balance between overall speed and population diversity. The specific methods of population division, motivations, and schemes for the moving and foraging phases are as follows.

#### 3.2.1. Fitness–Cosine Similarity Hybrid Population Partitioning

In nature, within a population of Genghis Khan sharks, the majority of individuals follow the fittest leader. However, some sharks do not blindly follow only this leader. Instead, they follow certain “scout” sharks that are relatively strong but deviate slightly from the leader’s direction. This behavior allows the group to explore potential directions for locating prey, thereby increasing the probability of a successful hunt and avoiding the risk of food shortage for the entire population that may result from an erroneous judgment made by the leader. Based on the above motivation, this section proposes a dynamic population partitioning method that combines cosine similarity and fitness criteria to guide targeted exploration. The rationale behind this method is to effectively segregate the population into superior and inferior subpopulations. While fitness serves as the most intuitive and fundamental indicator of an individual’s quality, relying solely on it presents challenges for complex multimodal problems. In such cases, the current best individual may reside on a different peak than the theoretical global optimum. Exclusive reliance on fitness can inadvertently guide the population to cluster excessively around the current best individual, thereby increasing the risk of falling into local optima. Conversely, individuals with relatively good fitness that differ significantly from the current best individual are highly likely to be located on alternative peaks. By allowing these individuals to participate in guiding the search, we significantly increase the probability of exploring other potential regions. Therefore, our method first selects top-performing individuals based on fitness to ensure rapid evolution and subsequently employs cosine similarity to retain diverse individuals. This strategy not only preserves the guiding role of the current best individual but also bolsters the exploration of other advantageous regions, effectively mitigating the risk of falling into local optima due to a single search direction. The partitioning procedure is as follows:

(1) Select the top *n* fittest individuals from the population (empirically, *n* = 15 yields robust performance, though it is adjustable for specific problems).

(2) Sort these *n* individuals in ascending order based on cosine similarity to the global best solution and extract the most dissimilar *N*_2_ individuals.

(3) Rank the remaining *n* − *N*_2_ individuals by fitness in ascending order and select the top *N*_1_ − *N*_2_ excellent solutions.

(4) Combine the *N*_1_ − *N*_2_ excellent individuals and *N*_2_ dissimilar individuals to form the superior subpopulation (size *N*_1_), whereas the remaining individuals constitute the inferior subpopulation.

The cosine similarity is computed via Equation (16), whereas the size of the superior population *N*_1_ and the size of dissimilar individuals *N*_2_ are shown in Equation (17) and Equation (18), respectively; $N_{\max}=10$ can get the best value.

(16) $$sim_{i,best}\left(t\right)=\frac{\sum_{j=1}^{D}X_{best,j}\left(t\right)\times X_{i,j}\left(t\right)}{\sqrt{\sum_{j=1}^{D}{\left(X_{best,j}\left(t\right)\right)}^{2}}\times \sqrt{\sum_{j=1}^{D}{\left(X_{i,j}\left(t\right)\right)}^{2}}}$$

(17) $$N_{1}=N_{\max}-\left\lceil 0.1\times N\times {\left(\frac{t}{Max\_iter}\right)}^{2}\right\rceil$$

(18) $$N_{2}=N_{1}-\left\lceil 0.6\times N_{1}-\frac{0.2\times N_{1}\times t}{Max\_iter}\right\rceil$$

Here, $sim_{i,best}\left(t\right)$ denotes the cosine similarity between the current individual and the optimal individual at iteration *t*, whereas $\left\lceil •\right\rceil$ represents the rounding operation, *N*_1_ is the size of the dominant individuals, and *N*_2_ is the size of the dissimilar individuals.

In summary, the proposed fitness–cosine similarity hybrid population partitioning method offers two key advantages:

(1) A dual-stage screening mechanism of “fitness-sort and cosine similarity” is adopted. Specifically, fitness-based primary selection prioritizes top-performing individuals to rapidly concentrate on high-performance regions, reducing computational waste in low-fitness areas and accelerating convergence. The following cosine similarity-based secondary screening prevents premature clustering around similar solutions and effectively maintains population diversity, helping escape local optima.

(2) The ratios of dissimilar individuals are dynamically adjusted. In the early stage, dissimilar individuals are fewer than similar ones, which enables rapid focusing on the potential optimal solution region and reduces ineffective search. As iterations progress, dissimilar individuals exceed similar ones, increasing the capacity to evade local optima, explore a broader solution space, and improve the chances of finding the global optimum.

#### 3.2.2. Enhanced Moving Phase

As described in Section 2.2, the original moving phase operates as follows: The first individual shifts toward the global best solution, while others move toward the midpoint between themselves and their predecessor, collectively pulling the population toward optimal regions. However, this mechanism exhibits three critical limitations:

(1) The first individual’s position update relies solely on the difference vector between itself and the global optimal solution, which only provides directional guidance. A lack of control over the base vector often results in overshooting, where the updated position diverges considerably from the current and optimal positions, preventing targeted exploration.

(2) For the other individuals moving toward the midpoint, the high dimensionality of the problem means that the intermediate positions cannot be guaranteed to exceed the original solutions.

(3) The adaptive step size *s* in Equation (3) mainly depends on individuals’ fitness values (*I*) and the random exponent (*r*). In the early stage or for complex problems, the fitness values and step size *s* are disproportionately large, causing frequent boundary violations and ineffective searches. Similarly, parameter *r* is randomly generated within [0, 1], without considering the requirements of the individual itself and the evolutionary stage of the parameters *r* and *s*, which affects the convergence performance of the algorithm.

An improved moving phase is proposed for the superior subpopulation to enhance the algorithm’s convergence speed, as formalized in Equation (19).

(19) $$X_{good\_i}^{j}(t+1)=X_{best1}^{j}(t)+s_{1}\times (X_{good\_r_{1}}^{j}(t)-X_{good\_r_{2}}^{j}(t))\;$$

Here, $X_{good\_i}^{j}(t+1)$ denotes updated current individual in the superior subpopulation; $X_{good\_r_{1}}^{j}(t)$ and $X_{good\_r_{2}}^{j}(t)$ indicate two individuals randomly chosen from the superior subpopulation at iteration *t*; the computation of *s*_1_ follows Equation (20); and the selection mechanism for $X_{best1}^{j}(t)$ is defined in Equation (21).

(20) $$s_{1}={\left(\frac{\left(fit\_\max-fit_{i}\right)}{\left(fit\_\max-fit\_\min\right)}\right)}^{{\left(\frac{t}{Max\_iter}\right)}^{2}}$$

(21) $$X_{best1}^{j}(t)=\left\{\begin{array}{cc} X_{best}^{j}(t) & if\;X_{i}=X_{best}\\ xpool_{rand}^{j}(t) & else \end{array}\right.$$

Here, $xpool_{rand}$ denotes an individual randomly selected from the set [$X_{i}$, $X_{best\_sbx}$, $X_{dis\_sbx}$], whereas the computational methods for $X_{best\_sbx}$ and $X_{dis\_sbx}$ are specified in Equation (22) and Equation (23), respectively.

(22) $$X_{best\_sbx}=0.5\times ((1+\beta_{u})\times X_{best}+(1-\beta_{u})\times X_{dis})$$

(23) $$X_{dis\_sbx}=0.5\times ((1+\beta_{u})\times X_{dis}+(1-\beta_{u})\times X_{best})$$

Here, $X_{dis}$ and $X_{best}$ represent the best individual from the dissimilar and similar subpopulation, respectively, whereas the computational formulation of $\beta_{u}$ is given by Equation (24).

(24) $$\beta_{u}=\left\{\begin{array}{cc} {\left(2\times rand\right)}^{\frac{1}{1+\eta }} & if\;rand<0.5\\ {\left(\frac{1}{2-2\times rand}\right)}^{\frac{1}{1+\eta }} & else \end{array}\right.$$

Here, *η* is typically set to 1, which can give the optimal effect; *rand* represents a random variable limited to the range of [0, 1].

In conclusion, the proposed moving mechanism exhibits distinct advantages over the GKSO algorithm. As formulated in Equation (19), the evolutionary process incorporates a base vector with two modes: If the current individual is the global optimum, it serves as the base vector to search around it, ensuring convergence direction. Otherwise, the base vector is randomly selected from three candidates: the current individual and two offspring generated via simulated binary crossover (SBX) between the global optimum and the best individual of the dissimilar subpopulation. The two offspring individuals generated through SBX maintain the evolutionary direction of the superior subpopulation, and the operation of SBX and using the current individual as the base vector sustain the diversity of the population. Moreover, the adaptive step size *s*_1_, operating under normalized fitness conditions, dynamically adjusts movement magnitudes according to iteration and fitness landscape variations. This not only complies with evolutionary requirements but also effectively addresses boundary violations caused by fixed-step approaches such as Equation (3).

#### 3.2.3. Enhanced Foraging Phase

Building upon the preceding analysis, the foraging phase in the original algorithm primarily conducts sophisticated exploitation around the global optimum and functionally resembles the moving phase in accelerating convergence. To balance convergence speed and population diversity better while improving overall efficiency, the proposed IGKSO algorithm implements a specialized division of labor: Superior individuals execute the enhanced moving phase to drive rapid convergence, whereas inferior individuals undergo a redesigned parabolic foraging phase to maintain diversity systematically.

To this end, a novel parabolic foraging mechanism, formalized in Equation (25), is introduced specifically for the inferior subpopulation.

(25) $$X_{bad\_i}(t+1)=X_{bad\_i}(t)+z\times r_{21}\times (X_{good\_rand}(t)-X_{bad\_i}(t))+z\times \lambda \times {p_{1}}^{2}\times (X_{r1}(t)-X_{r2}(t))$$

Here, $X_{bad\_i}$ denotes the position of current individual in the inferior subpopulation; the subspace selection vector $z\in {\left[0,1\right]}^{\dim}$ is a binary mask with each element randomly sampled to determine the active dimensions, dim∈[1,D]; $X_{good\_rand}$ represents an individual randomly chosen from the superior subpopulation; the terms $X_{r1}$ and $X_{r2}$ correspond to two distinct individuals randomly selected from the current population; and the computational procedures for the weighting coefficients $r_{21}$ and $p_{1}$ is rigorously defined by Equations (26) and (27).

(26) $$r_{21}=\left\{\begin{array}{cc} 0.2+{\left(1-\frac{t}{Max\_iter}\right)}^{0.25} & if\;\frac{t}{Max\_iter}<0.5\\ 0.2+0.3\times {\left(\frac{t}{Max\_iter}\right)}^{2} & else \end{array}\right.$$

(27) $$p_{1}=p/2$$

The proposed mechanism for the inferior subpopulation demonstrates three critical improvements compared with the foraging phase in GKSO. First, the base vector shifts from the global best individual to the current inferior individual and intentionally sacrifices convergence velocity for improved diversity maintenance, which is the foraging phase’s core objective. Moreover, this approach substantially mitigates premature convergence to local optima, which may occur when the current best individual resides within a local optimum. Second, both difference vectors undergo strategic redesign: (1) The first vector maintains evolutionary direction by learning from a randomly selected superior individual rather than exclusively following the global optimum while reducing premature convergence, even if slowing the convergence speed. (2) The second difference vector is generated through information exchange between two randomly selected individuals from the population, instead of fixed elite-current pairs, which improves diversity maintenance. Moreover, the subspace mode *z* probabilistically deactivates specific dimensions during updates. This stochastic dimensional selection for local search reduces the probability of diversity loss caused by rapid clustering toward superior individuals along specific dimensions. (3) Instead of employing a random number within the [0, 2] interval from the initial *r*_2_, the improved *r*_2_ (*r*_21_) of this study adopts a deterministic parameter that gradually varies with the iteration, as defined in Equation (26), which, while reducing a certain randomness, still accelerates the convergence speed while sustaining sufficient population diversity and better meeting the optimization requirements of different test functions; *p*_1_ is the improved parameter derived from *p*, and its value range is transformed from [0, 2] to [0, 1], which, although narrowing the exploration scope, also avoids the boundary overflow problem. Compared with the original coefficients *p*^2^ and *r*_2_, the improved *p*^2^ and *r*_2_, namely *p*_1_^2^ and *r*_21_, are presented in Equations (26) and (27) in this section. When used together, they can better satisfy the evolutionary requirements at different stages. Specifically, in the early stage, the initial value of *r*_21_ is 1.2, slightly larger than the initial value of *p*_1_^2^, which is 1. This causes the learning degree from the first difference vector to be slightly higher than that from the second one, enables the population to conduct extensive exploration to maintain diversity while accelerating the convergence speed, avoids premature convergence, and increases the possibility of converging to the optimum for different test functions. In the later stage, similarly, *r*_21_ > *p*_1_^2^, which ensures that the algorithm accelerates the convergence speed when the evolution direction has been determined while preventing excessive exploration from disrupting the original evolution direction.

### 3.3. Improved Self-Protection Phase

As described in Equation (7), the individual update mechanism fundamentally follows the formulation “base vector + step 1 × difference vector 1 + step 2 × difference vector 2 + step 3 × difference vector 3.” The roles of each component are as follows:

(1) It is well known that the updated individual is generated in the vicinity of the base vector, where the base vector in the GKSO algorithm is selected between the optimal individual and the current individual. Obviously, using the current individual as the base vector effectively maintains population diversity, while employing the optimal individual as the base vector enhances the algorithm’s convergence speed. In summary, the selection of the base vector balances the convergence speed and population diversity to a certain extent.

(2) Difference vector 1 lies on the mutual learning between $X_{k}$ and the optimal individual. Among them, learning from the optimal individual ensures the evolutionary direction and accelerates the convergence speed, whereas the other one $X_{k}$ is generated through dimensional crossover between a randomly initialized individual and a randomly selected individual within the population, which introduces new genes into certain dimensions of the random individual and enables the current population to explore other positions.

(3) Difference vectors 2 and 3 correspond to mutual learning between two randomly selected individuals within the population and between two randomly initialized individuals, respectively. Difference vector 2 effectively maintains population diversity, while difference vector 3 provides the population with new genes, which avoids premature convergence to local optima. In summary, the primary objective of the self-protection phase is to balance the algorithm’s convergence speed and population diversity.

Extensive experimental studies have demonstrated that the GKSO algorithm fails to converge to the global optimum when tackling complex optimization problems rapidly. To further strengthen the algorithm’s capacity for balancing speed and diversity, a novel self-protection mechanism is introduced, as mathematically described by Algorithm 3.
**Algorithm 3 The Pseudo-Code of Self-Protection****Input**: Population: *X*; Parameter: $k_{3}$, $k_{4}$, ρ, $a_{4}$, $a_{5}$, $a_{6}$, $a_{7}$, *nVOL*, *LIM* **Output**: The optimal solution and its fitness value
1. Implement Equation (30) to generate $X_{k1}(t)$2. Implement Equation (32) to generate $k_{4}$3. Implement Equation (33) to generate $k_{3}$4. Implement Equations (34)–(37) to generate $a_{4}$, $a_{5}$, $a_{6}$, $a_{7}$5. Implement Equations (38)–(40) to generate *nVOL*6. **If** *nVOL < LIM* **do**7.   $X_{i}^{t+1}\leftarrow X_{i}^{t}$ as described in Equation (28).8. **Else**9.   $X_{i}^{t+1}\leftarrow X_{i}^{t}$ as described in Equation (29).10.**End if**11.Implement Equation (31).


(28) $$X_{i}^{j}(t+1)=X_{i}^{j}(t)+k_{3}a_{4}(X_{good1\_rand}^{j}(t)-X_{k1}^{j}(t))+k_{4}\rho (a_{6}(X2^{j}(t)-X1^{j}(t)))\times U+a_{5}(X_{u1}^{j}(t)-X_{u2}^{j}(t))$$

(29) $$X_{i}^{j}(t+1)=X_{good1\_rand}^{j}(t)+k_{3}a_{7}(X_{good1\_rand}^{j}(t)-X_{k1}^{j}(t))\;+k_{4}\rho (a_{6}(X2^{j}(t)-X1^{j}(t)))\times U+a_{5}(X_{u1}^{j}(t)-X_{u2}^{j}(t))/2$$

Here, $X_{u1}$, $X_{u2}$, *X*2, *X*1 and ρ are precisely identical to those in the original GKSO; $X_{good1\_rand}$ represents an individual randomly chosen from the top five candidates sorted by fitness; $X_{k1}$ is a novel individual generated by Equation (30); the parameter *U* is shown in Equation (31); *k*_4_ denotes a stochastic variable with zero mean and variance σ^2^, where σ^2^ is specified in Equation (32); the improved parameter *k*_3_ is shown in Equation (33); the enhanced parameters *a*_4_, *a*_5_, *a*_6_ and *a*_7_ are presented in Equations (34)–(37), respectively; *LIM* is a threshold value, typically set to 0.15, which can yield good results and may be adjusted manually; and nVOL represents the diversity-judgment condition, the details of which are provided in Equation (38).

(30) $$X_{k1}^{j}(t)=l_{2}\times \left(X_{i}^{j}(t)-X_{p}^{j}(t)\right)+X_{p}^{j}(t)$$

Here, the selection methods of $X_{p}\left(t\right)$ and *l*_2_ are precisely identical to those in the original GKSO algorithm.

(31) $$U=\left\{\begin{array}{cc} 1 & if\;X_{i}^{j}(t+1)-X_{i}^{j}(t)<1e-10\\ 0 & else \end{array}\right.$$

(32) $$\sigma^{2}=1-{\left(\frac{t}{Max\_iter}\right)}^{2}$$

(33) $$k_{3}=(-1+2\times rand)\times (1-{\left(\frac{t}{Max\_iter}\right)}^{0.25})$$

(34) $$a_{4}=l_{1}\times 2\times (0.5+0.5\times \sin(\frac{\pi \times t}{Max\_iter}))+(1-l_{1})$$

(35) $$a_{5}=l_{1}\times (0.5+0.5\times (1-\cos(\frac{\pi \times t}{2\times Max\_iter})))+(1-l_{1})$$

(36) $$a_{6}=l_{1}\times (0.5+0.5\times \cos(\frac{\pi \times t}{Max\_iter}))+(1-l_{1})$$

(37) $$a_{7}=l_{1}\times 2\times (0.5+0.5\times (1-\sin(\frac{\pi \times t}{Max\_iter})))+(1-l_{1})$$

(38) $$nVOL=\sqrt{\frac{V_{pop}}{V_{lim}}}$$

Here, $V_{lim}$ and $V_{pop}$ denote the hypervolume of the entire search space and the whole population’s space, respectively, and their specific calculation methods are presented in Equation (39) and Equation (40), respectively.

(39) $$V_{lim}=\sqrt{\prod_{i=1}^{D}|ub_{i}-lb_{i}|}$$

(40) $$V_{pop}=\sqrt{\prod_{i=1}^{D}|\frac{\left(u_{x_{i}}-l_{x_{i}}\right)}{2}|}$$

Here, ub and *lb* denote the upper and lower limits of the search space, respectively; $u_{x_{i}}$ and $l_{x_{i}}$ symbolize the upper and lower bounds of the current population; ∏ represents the cumulative symbol; and $\left|\cdot \right|$ denotes the absolute value sign.

To sum up, compared with the self-protection stage of the GKSO algorithm, the improved self-protection phase offers the following merits: First, although the new selection of the base vectors slows down the population’s evolution speed within a specific range, while ensuring its evolution direction, it also adds new evolution directions, which greatly reduces local optima entrapment. Additionally, in the original self-protection stage, the selection condition of the base vector is controlled by *a*_1_, which takes a value of 2 × *rand* or 1, with a range of [0, 2]. Compared with the fixed threshold of 0.5, this results in the execution proportion of the optimal individual and the current one being approximately 3:1, which makes this stage overly dependent on the optimal individual and induces the algorithm to quickly converge to the region where the optimal value is located. This research direction is singular. Moreover, if the current optimal solution is a local optimal solution, it still leads to an inability to escape the local peak. However, the diversity assessment condition proposed in this section can better achieve the sophisticated regulation of the exploration–exploitation based on population aggregation state. Second, in difference vector 1, $X_{k}$ and the optimal individual $X_{best}$ are replaced by $X_{k1}$ and $X_{good1\_rand}$. The composition of $X_{k1}$ replaces the randomly initialized individual with the current individual, which, to a certain extent, avoids the loss of effective evolutionary information caused by random initialization and the invalid search caused by randomly generated individuals deviating from the current promising regions. Although the replacement method for $X_{best}$ slows down the convergence speed to a certain extent, it increases the directions of population evolution, provides greater population diversity, and reduces the possibility of the algorithm falling into local optima. In addition, in the original mutual learning mode, the difference vector includes $a_{1}\times X_{best}$ and $a_{2}\times X_{k}$. $a_{1}\times X_{best}$ and $a_{2}\times X_{k}$ represent some dimensions of the optimal individual and the current individual whose genes are changed to 2 *× rand* times and *rand* times of their original gene values, which provides more population diversity. However, because it completely cuts off the intrinsic connections between dimensions, the quality of $a_{1}\times X_{best}$ and $a_{2}\times X_{k}$ cannot be judged, and the evolution-direction ensuring the algorithm’s convergence cannot be guaranteed. However, the difference vector 1 designed in this section cancels the cross-dimension operations of the original algorithm, establishes a coordinated evolution mechanism among dimensions, and ensures the algorithm’s evolution direction. Third, regarding difference vector 2, compared with the original difference vector, this section only modifies the step size of difference vector 2, adds the “*×U*” term, and replaces the value of *a*_3_ with *a*_6_. The role of the “*×U*” term is as follows: When certain dimensions of individuals within the population become trapped in local optima, this improved method will determine whether a local optimum has been encountered based on the evolution of certain dimensions of individuals within the population before and after updating. If certain dimensions of individuals in the population show little change before and after updating, that is, when $X_{i}^{j}(t+1)-X_{i}^{j}(t)<1e-10$ is satisfied, this difference vector is introduced to provide the population with more diversity, enhancing the algorithm’s ability to escape local optima and facilitating further evolution. Otherwise, the difference vector is not activated in the corresponding dimensions, reducing interference with the current population’s evolution direction and thus improving convergence speed. Fourth, for difference vector 3, compared with the original version, when population diversity is insufficient, that is, when *nVOL* < 0.15, the operation of “×0.5” in the step size is removed, which increases the exploration extent of this component and further supplements population diversity. Fifth, regarding the comparative roles of the various parameters, *a*_1_, *a*_7_ and *a*_4_ control the step size of the difference vector 1; *a*_2_ and *a*_5_ control the step size of the difference vector 3; *a*_6_ and *a*_3_ control the step size of the difference vector 2; where *k*_1_*, k*_2_, *k*_3_ and *k*_4_ govern the direction. Compared with *k*_1_, *k*_3_ introduces the iteration count, with its magnitude decreasing as the number of iterations increases. This facilitates global exploration in the early stage and fine-grained local search in the later stage. Compared with *k*_2_, *k*_4_ replaces the standard deviation of 1 with σ, reducing the probability of taking values exceeding 1. Additionally, this part introduces boundary handling, confining the value range to [0, 1], which reduces the possibility of boundary violations. Next, comparing *a*_1_ with *a*_4_ and *a*_7_, comparing *a*_2_ with *a*_5_ and comparing *a*_3_ with *a*_6_ respectively: Relative to *a*_1_ (a random number in [0, 2]), *a*_4_ is a sine function in [1, 2] that first increases then decreases with iterations, reducing randomness, expanding the range of difference vector 1, and accelerating convergence. Similarly, *a*_7_ is an inverse sine function in [1, 2] that first decreases then increases, serving the same purpose; Compared with *a*_2_ (a random number in [0, 1]), *a*_5_ is a cosine function in [0.5, 1] that guides mutual learning between two randomly selected individuals. Although the value range is shortened, the probability of larger values increases, helping maintain population diversity. Additionally, the iteration dependence better aligns with the population evolution direction. Regarding *a*_3_ and *a*_6_, their value ranges remain unchanged, but *a*_6_ gradually decreases with iterations. This design maintains diversity in the early stage when individuals are dispersed, and in the later stage when diversity is poor, it reduces the risk of disrupting the evolution direction while avoiding boundary overflow caused by randomness. From a holistic perspective: In the early stage (individuals dispersed, diversity good), the condition nVOL ≥ 0.15 is executed. Here, *a*_7_ has a larger range than *a*_5_ and *a*_6_, making difference vector 1 dominant, which accelerates convergence while maintaining diversity. In the later stage (individuals clustered, diversity poor), the other formula is executed. Here, *a*_4_ and *a*_5_ have similar values, reducing the influence of difference vector 1 and balancing it with difference vector 2 to maintain diversity.

### 3.4. Comparative Analysis of GKSO and IGKSO

To further clarify the differences between the IGKSO and GKSO algorithms, this section introduces Wilcoxon rank-sum tests with a significance level of 5% for the IGKSO algorithm [26] and the Nemenyi post hoc test [27].

The Wilcoxon rank-sum test serves as a non-parametric statistical tool for evaluating differences between two independent samples. In this work, a *p*-value exceeding 0.05 suggests that no statistically significant distinction exists between the algorithms being compared, which is indicated by the symbol “=”. On the other hand, when the *p*-value falls below 0.05 and the average optimal objective value obtained by the enhanced algorithm across 20 independent runs is superior to that of the IGKSO algorithm, the enhanced algorithm is regarded as significantly better, denoted by “+”. Conversely, if the *p*-value is below 0.05 and the IGKSO algorithm achieves a better mean optimal value over the 20 trials, the enhanced algorithm is considered significantly worse, represented by “−”. The decision criteria for the post hoc tests are summarized as follows. If the *p*-value is less than 0.01, the null hypothesis is rejected at the 1% significance level, indicating a highly significant difference. If the *p*-value is between 0.01 and 0.05, the null hypothesis is rejected at the 5% significance level, indicating a significant difference. If the *p*-value is between 0.05 and 0.1, the null hypothesis is rejected at the 10% significance level, indicating a marginally significant difference. If the *p*-value is greater than or equal to 0.1, the null hypothesis is accepted, indicating that the two algorithms have similar statistical performance.

The specific experimental results and the experimental setup are as follows: the number of populations in this section of the experiment N = 50; the maximum number of function evaluations max_FE = 100,000 and the maximum number of iterations Max_iter = 1000 for the CEC2019 test suite; the maximum number of function evaluations *max_FE* = 300,000 and the maximum number of iterations *Max_iter* = 3000 for the CEC2017 test suite; the termination condition for all experiments are the maximum function evaluations. Each entry is presented over 20 independent runs.

Based on the comprehensive analysis of the Nemenyi post hoc test in Table 1 and the Wilcoxon rank-sum test results in Table 2, the following conclusions can be drawn.

In the Nemenyi test on the CEC2017 benchmark suite, IGKSO demonstrates statistically significant superiority over all other compared algorithms. On the CEC2019 benchmark suite, IGKSO shows marginally significant superiority over MDBO, while maintaining significant superiority over GKSO, MGKSO, TTHHO, IGKSO1, IAAO, EGKSO, ALA, and MTV-SCA.

In the Wilcoxon rank-sum test, the notation “+/=/−” indicates that IGKSO is inferior to, equivalent to, or superior to the compared algorithm, respectively. Based on the counts of “+”, “=”, and “−”, it is evident that IGKSO holds a clear advantage over the other compared algorithms on both the CEC2017 and CEC2019 benchmark suites.

Overall, as an improved variant of GKSO, IGKSO effectively addresses the limitations of the original algorithm, such as premature convergence and insufficient solution quality. The experimental results validate the effectiveness of the proposed improvements, demonstrating that IGKSO is a competitive and reliable optimizer for global optimization problems.

### 3.5. Time Complexity

Given that time complexity is a key metric for evaluating algorithmic performance, this section analyzes the time complexity of both IGKSO and GKSO. Generally, the overall computational cost is primarily determined by two core components: the initialization process and the individual position updates. To quantify this, we define the following variables: let *N* denote the population size, *D* the dimensionality of the optimization problem, and *Max_iter* the maximum number of iterations. Based on these settings, the time complexity analysis for both algorithms is as follows:

The GKSO algorithm mainly involves population initialization and position updates across four stages: hunting, moving, foraging, and self-protection. Since population initialization occurs only once and does not participate in the iterative loop, its complexity is O(N×D). As the complexity of each stage in every iteration is also O(N×D), the total complexity of GKSO is calculated as:

(41) $$\begin{array}{cc} O(GKSO) & =O(Initialization)+O(Hunting)+O(Moving)+O(Foraging)+O(Self-Protection)\\ & =O(N\times D)+O(4\times N\times D\times Max\_iter)\\ & =O(N\times D\times (1+4Max\_iter)) \end{array}$$

In the proposed IGKSO algorithm, the sub-space search strategy activates only a subset of dimensions in each iteration. For a rigorous analysis, we calculate the complexity based on the worst-case scenario (i.e., assuming all dimensions are involved in the update). The complexity is derived as:

(42) $$\begin{array}{ll} O(GKSO) & =O(Initialization)+O(Hunting,Moving,Foraging)+O(Self-Protection)\\ & =O(N\times D)+O(2\times N\times D\times Max\_iter)\\ & =O(N\times D\times (1+2Max\_iter)) \end{array}$$

By comparing the complexities of IGKSO and GKSO, it is evident that IGKSO achieves a significant improvement in performance with only a negligible amount of additional computational overhead, perfectly balancing computational efficiency with optimization capability.

## 4. Results and Analysis

To evaluate the performance of the IGKSO algorithm comprehensively, this section presents experiments divided into three parts: (1) parameter validity analysis; (2) verifying the effectiveness of the proposed three improvement strategies; (3) comparison of the performance of the improved algorithm IGKSO with the original algorithm and eight excellent algorithms; and (4) application to Photovoltaic Model Parameter Extraction. All experiments employ two benchmark suites: CEC2017 [28] and CEC2019 [29]. The CEC2017 test set comprises 30 benchmark functions, categorized as follows: unimodal functions (F1–F3), with F2 excluded due to its non-applicability for testing; multimodal functions with local minima (F4–F10); hybrid functions constructed by combining three or more CEC2017 benchmark functions via rotation or translation (F11–F20); and composite benchmark functions formed by combining at least three mixed functions or CEC2017 benchmark functions with rotation and translation (F21–F30). The CEC2019 test set contains 10 single-objective test functions with varying dimensionalities and search ranges. Detailed information on the two test sets can be found in the literature. To ensure a fair comparison of the algorithms, all comparative algorithms are implemented in MATLAB R2018a on a Windows 10 platform with an Intel(R) Core (TM) i5-1035G1 CPU @ 1.00 GHz 1.19 GHz processor.

### 4.1. Parameter Validity Analysis

The proposed algorithm involves a relatively large number of parameters. For ease of reference, all parameters along with their meanings and value ranges are summarized in Table 3. Based on this, sensitivity analysis is conducted on several key parameters that significantly affect algorithm performance, including *n*, *k*, *η*, *r*_21_, and *LIM*.

To verify the validity of the above parameters, the experimental setting, the mean and standard deviation of the results and the Wilcoxon rank-sum test [26] with a significance level of 5% on the CEC2019 test set will be analyzed in Table 4, Table 5, Table 6 and Table 7. To ensure the fairness of the validation, the number of populations in this section of the experiment *N* = 50, the maximum number of function evaluations *max_FE* = 100,000 and the maximum number of iterations *Max_iter* = 1000. The termination condition for all experiments is the maximum number of function evaluations. Similarly, to verify the validity of the values taken, the performance of the IGKSO algorithm is verified when the values of *k* are taken to be 50, 100 and 200, respectively; similarly, it is also verified when the values of *n* are taken to be 13, 15 and 20, when the values of *LIM* are taken to be 0.05, 0.10, 0.15 and 0.20, when the values of *η* are taken to be 1, 2, 5, 10 and 20, and when the values of *r*_21_ fall within the corresponding interval range. Among them, in Table 7, when *t/Max_iter* < 0.5, the numerical interval of *r*_21_ represented in the first line; otherwise, the numerical interval of *r*_21_ represented in the second line. The function that achieves the best result is black-labeled on each function. The specific content of the Friedman test is as follows:

The Friedman test is adopted as a non-parametric method to rank multiple algorithms across a variety of test functions. This procedure assigns a rank to each algorithm on every test function and subsequently computes its average rank across all functions. A lower average rank reflects a better overall standing and more robust comprehensive performance.

To further investigate the impact of parameters *n*, *N*_1_ and *N*_2_ on the population partitioning strategy across different dimensions, a sensitivity analysis on the CEC2017 test suite was designed for dimensions of 10, 30, and 50, as shown in Table 8, Table 9 and Table 10, where each entry is presented over 20 independent runs. The specific experimental settings are as follows: the number of populations in the experiment *N* = 50, the maximum number of function evaluations *max_FE* = 300,000 and the maximum number of iterations *Max_iter* = 3000. The termination condition for all experiments is the maximum number of function evaluations. To verify the validity of the parameter values, the performance of the IGKSO algorithm is evaluated with *n* set to 13, 15, and 20, and *N*_1_ set to 9, 10, and 11, respectively. In this process, *N*_2_ varies dynamically with *N*_1_. The function that achieves the best result is black-labeled on each function.

Based on the results in Table 4, the Wilcoxon signed-rank test regarding parameters *n* and *k* reveals that although the configurations with *k* = 50 or 200 and *n* = 13 or 20 yield performance comparable to that of *n* = 15 and *k* = 100 on most test functions and are inferior only on one function, the mean metric indicates that IGKSO achieves optimal performance when *n* = 15 and *k* = 100. As shown in Table 5, regarding mean performance, IGKSO attains the global optimum on 90% of the functions solely when *LIM* = 0.15, a benchmark that other parameter settings fail to match. Furthermore, Wilcoxon signed-rank test results, using *LIM* = 0.15 as the baseline, indicate that IGKSO yields comparable performance across all functions when *LIM* = 0.10 or 0.20. Conversely, when *LIM* = 0.15, the algorithm outperforms the baseline on only one function while showing comparable performance on the remainder. Consequently, the optimal results are obtained when *LIM* is set to 0.15. Similarly, in terms of the number of optimal solutions obtained, *η* = 1 also achieves the global optimum on 90% of the functions. Taking this setting as the baseline, the configuration with *η* = 10 or 20 outperforms the baseline on only one function. Considering these findings collectively, setting *η* = 1 yields the best results. Based on the results in Table 7, it is evident that IGKSO achieves the global optimum on all functions when *r*_21_ transitions from an initial range of [1.2, 0.2] to a final range of [0.2, 0.5]. Furthermore, taking this configuration as the baseline, no other parameter settings yield superior results. Consequently, the IGKSO algorithm exhibits optimal performance when *r*_21_ varies from [1.2, 0.2] in the early stage to [0.2, 0.5] in the later stage.

As shown in Table 8, Table 9 and Table 10, with *n* = 15 and *N*_max_ = 10, IGKSO achieves the highest number of global optima across all dimensions (10, 30, and 50).

### 4.2. Empirical Investigations into the Efficacy of Individual Enhancement Strategies

In order to evaluate the effectiveness of each improved strategy, we formed three improved IGKSO variants by systematically adding a single strategy. The three new improvement algorithms are the new variant formed by integrating the individual update strategy from Section 3.1 with GKSO, the new variant formed by integrating the individual update strategy from Section 3.2 with GKSO, and the new variant generated by integrating the individual update strategy from Section 3.3 with GKSO. These algorithms are subsequently compared with the GKSO and IGKSO algorithms using the CEC2019 test suite. For clarity, the three enhanced algorithms are denoted as IGKSO11, IGKSO2, and IGKSO3, respectively. Furthermore, to further investigate the effectiveness of the subspace search strategy versus the full-space search strategy, this section compares the complete IGKSO with a variant stripped of the subspace search strategy, referred to as IGKSO4.

To ensure a fair comparison, all methods are evaluated under identical experimental settings: a population size of *N* = 50 and a maximum of 100,000 function evaluations (*max_FE*). The termination condition for all experiments is the maximum number of function evaluations. Furthermore, each algorithm is independently executed 20 times on every test function to reduce the likelihood of bias resulting from a single, potentially anomalous run.

The experimental results of all algorithms on the CEC2019 test suite are summarized in Table 11, where each entry is presented over 20 independent runs. To examine the statistical differences between the IGKSO algorithm and its enhanced variants, we conducted the Wilcoxon rank-sum test with a significance level of 5% and the Friedman test [26]. The detailed outcomes of these statistical tests are reported in Table 12 and Table 13. Similarly, the comparative experiments regarding the subspace and full-space search strategies are presented in Table 14, where the symbols “+/=/−” denote the results of the Wilcoxon signed-rank test.

As can be seen from Table 12 and Table 13, IGKSO11 achieves significantly better performance than GKSO on 6 functions, is significantly inferior on 2 test functions, and exhibits comparable performance on the remaining 2 test functions. IGKSO2 yields significantly better results on 7 test functions, significantly worse results on 1 test function, and similar performance on 2 test functions. IGKSO3 is significantly superior to GKSO on 5 test functions, significantly inferior on 1 test function, and comparable on 4 test functions. IGKSO outperforms GKSO on 8 test functions and shows similar performance on 2 test functions. Furthermore, it is observed that the rank average of the improvement algorithms, after adding the corresponding improvement strategy in GKSO, surpasses that of the GKSO algorithm, which indicates that the three improved strategies proposed in this paper have an impact on the performance of the GKSO algorithm and that the improved moving phase and foraging phase in Section 3.2 have the greatest impact on the GKSO algorithm.

Based on the results shown in Table 14, compared with the full-space search strategy, the subspace search strategy enables the IGKSO algorithm to achieve the global optimum on more functions. Furthermore, as indicated by the symbols “+/−/=”, IGKSO4 does not outperform IGKSO on any function. Therefore, both comparative approaches demonstrate the superiority of the subspace search strategy.

### 4.3. Comparative Analysis of the IGKSO Algorithm in Relation to Other Algorithms

For a comprehensive assessment of the IGKSO algorithm’s optimization capabilities, a set of comparison algorithms is introduced, which mainly consist of two categories: The first category consists of improved variants of the GKSO algorithm, which are used to verify the extent of improvement achieved by the proposed IGKSO over the original GKSO. These mainly include IGKSO1, EGKSO, and MGKSO. The second category selects representative metaheuristic algorithms published in recent years as comparison algorithms to verify the overall optimization capability of the proposed IGKSO in the current evolutionary computation field. These include IAOA, MTV-SCA, MDBO, TTHHO and ALA [30]. To guarantee an equitable comparison, all algorithms are evaluated under strictly uniform conditions: for CEC2017, population size *N* = 50, dimensionality *D* = 30, and maximum function evaluations *max_FE* = 300,000. For CEC2019, population size *N* = 50 and *max_FE* = 100,000. The termination condition for all experiments is the maximum number of function evaluations. The parameter configurations of the comparative algorithms are presented in Table 15, which ensures that the parameters used for each comparison algorithm are consistent with those reported in the original papers.

#### 4.3.1. Comparison of IGKSO Algorithm with Other Algorithms Regarding Convergence Accuracy

To evaluate the convergence accuracy of the IGKSO algorithm against other methods, this section employs the CEC2019 and CEC2017 benchmark suites, where the dimension of the CEC2017 test set is 30. The statistical findings for the IGKSO algorithm and its competitors are presented in Table 16 and Table 17 based on 20 independent runs conducted on the CEC2017 (D = 30) and CEC2019 benchmark suites, respectively. The boldfaced data indicate the best performance achieved on each test function. For a more comprehensive comparison between the IGKSO algorithm and the other algorithms, Table 18, Table 19, Table 20, Table 21, Table 22 and Table 23 report the results of the Wilcoxon rank-sum test at a 5% significance level, the Friedman test and the Nemenyi post hoc test.

To further identify pairwise differences among the algorithms after the Friedman test, we conducted the Nemenyi post hoc test. The Nemenyi post hoc test is a widely adopted method for multiple comparisons in non-parametric statistical analysis. It determines whether the performance difference between two algorithms is statistically significant by calculating a *p*-value. The detailed computational procedure is described as follows.

(1) Calculate a test statistic(Q1) according to Equation (43).

(2) Calculate a *p*-value according to Equation (44).

(43) $$Q1=\left|Rank_{i}-Rank_{j}\right|/\sqrt{\frac{lk(lk+1)}{6H}}$$

Here, *lk* is the number of algorithms; *H* is the number of test functions; $Rank_{i}-Rank_{j}$ denotes the difference between the mean ranks of two algorithms; and $\left|\cdot \right|$ represents the absolute value.

(44) p=P(Q≥Q1|lk,∞)

Here, *Q* is the Studentized range distribution.

As shown in Table 16, on the 30-dimensional CEC2017 test suite, IGKSO achieves global optima on F1, F3, F6, F9, F11, F13–F15, F18–F20, F22, F25, and F30; ALA attains global optima on F4, F12 and F25; IGKSO1 obtains the global optimum on F10; MDBO secures global optima on F5, F7, F17, F21, F22, F24, F26, F28, and F29; IAOA attains global optima on F8, F16, and F23; TTHHO obtains global optima on F27; and the remaining comparison algorithms did not achieve the global optimal value on any function. Based on the number of global optima obtained, the IGKSO algorithm achieves the highest convergence accuracy. Specifically, for unimodal functions, the proposed IGKSO algorithm achieves the best accuracy; for multimodal functions, only a few algorithms manage to surpass IGKSO in specific cases: ALA achieves higher convergence accuracy than IGKSO solely on function F4, while MDBO and IAOA perform better on F4, F5, F7, and F8. IGKSO1 outperforms IGKSO only on F4 and F10, MTV-SCA only on F5 and F8, and EGKSO only on F4 and F10. The remaining algorithms fail to exceed IGKSO’s accuracy across all multimodal test functions; for hybrid functions, IGKSO maintains a leading position in most cases, with exceptions being ALA on F12, IAOA on F16 and MDBO on F17, where each slightly outperforms IGKSO. No other competitor achieves better results on any hybrid function. Among composite functions, MDBO shows better accuracy on F21, F23, F24, F26, F28, and F29, while matching IGKSO on F22. ALA matches IGKSO on F25 but underperforms on all others. MTV-SCA surpasses IGKSO only on F21 and F24, IAOA only on F21, F23 and F26, MGKSO only on F24, and IGKSO1 and EGKSO only on F28. All other comparison algorithms do not exceed IGKSO’s convergence accuracy on any composite test function, underscoring the robustness and general applicability of the proposed method. As shown in Table 18, according to the significance indicated by the Wilcoxon rank-sum test symbols “+/−/=”, the final row of the table reveals distinct comparative performance among the algorithms. Notably, GKSO, EGKSO, MGKSO, and MTV-SCA fail to surpass IGKSO on any of the test functions. In contrast, IGKSO1 and ALA outperform IGKSO on two functions, while TTHHO achieves superiority in only one case, and IAOA outperforms IGKSO on six functions. MDBO exhibits the strongest relative performance, exceeding IGKSO on nine functions. Regarding ties, GKSO matches IGKSO on two functions but is outperformed on 27; IGKSO1 performs equally well on three functions but is inferior on 24; and EGKSO ties on four functions while being weaker on 25. Both MGKSO and TTHHO show no ties with IGKSO: MGKSO is worse on all 29 functions, and TTHHO is outperformed on 28. IAOA matches IGKSO twice and falls short on 21 functions. MTV-SCA achieves equal performance in 10 cases but remains weaker in 19. MDBO ties on six functions and is inferior on 14, whereas ALA matches IGKSO on three functions and underperforms on 24. Collectively, these results underscore IGKSO’s robust performance across the majority of test functions, with only MDBO demonstrating a notable competitive advantage in a limited subset of cases. As shown in Table 20, IGKSO ranks first on the CEC2017 benchmark set compared to the other competing algorithms.

On the CEC2019 test suite, IGKSO still demonstrates strong competitive performance. As illustrated in Table 17, IGKSO achieves global optima on F1, F2, F6, F7, F8, and F10, including the theoretical optima on F1 and F6. While IGKSO1, EGKSO, MGKSO, MDBO, and TTHHO also achieve the theoretical optimum on F1, only MDBO attains the global optimum on F9, and IAOA does so on F4 and F5. In terms of comparative superiority, EGKSO, MTV-SCA, IAOA, and MDBO outperform IGKSO solely on function F5, whereas IGKSO1, IAOA, and MDBO surpass it only on F9. Additionally, IAOA demonstrates superior performance to IGKSO only on function F4. Based on the total number of functions for which each algorithm achieves the best performance, IGKSO clearly demonstrates superior overall effectiveness compared to the other algorithms. According to the last row of Table 19, it can be observed that MGKSO and TTHHO do not outperform IGKSO on any test function, and each achieves performance equivalent to IGKSO on only one function while underperforming on the remaining nine. Similarly, GKSO, EGKSO, and MDBO also fail to surpass IGKSO on any function. Among them, GKSO matches IGKSO on two functions and is inferior on eight; MDBO achieves equal performance on six functions and is weaker on four; and EGKSO ties on four functions and underperforms on six. Notably, ALA is outperformed by IGKSO across all functions. In contrast, both IGKSO1 and MTV-SCA manage to surpass IGKSO on one function each. Specifically, IGKSO1 equals IGKSO on three functions and falls short on six, whereas MTV-SCA, apart from its single superior result, underperforms on all other functions. As for IAOA, it outperforms IGKSO on two functions, achieves equivalent performance on one function, and underperforms on the remaining seven functions. Overall, IGKSO demonstrates consistently superior or competitive performance across the majority of the tested functions. As shown in Table 21, IGKSO demonstrates the lowest average rank among all nine algorithms, which indicates its superior performance.

According to Table 22 and Table 23, along with the decision criteria of the post hoc test, any pair of algorithms not marked in bold exhibits a statistically significant difference. In contrast, the bolded pairs indicate a marginally significant difference, meaning that the *p*-value is close to but slightly above the conventional significance level.

In summary, in comparison with other competing algorithms, the proposed IGKSO algorithm exhibits notable advantages in convergence accuracy.

#### 4.3.2. Comparison of IGKSO Algorithm with Other Algorithms Regarding Convergence Rate

**Figure 3:**
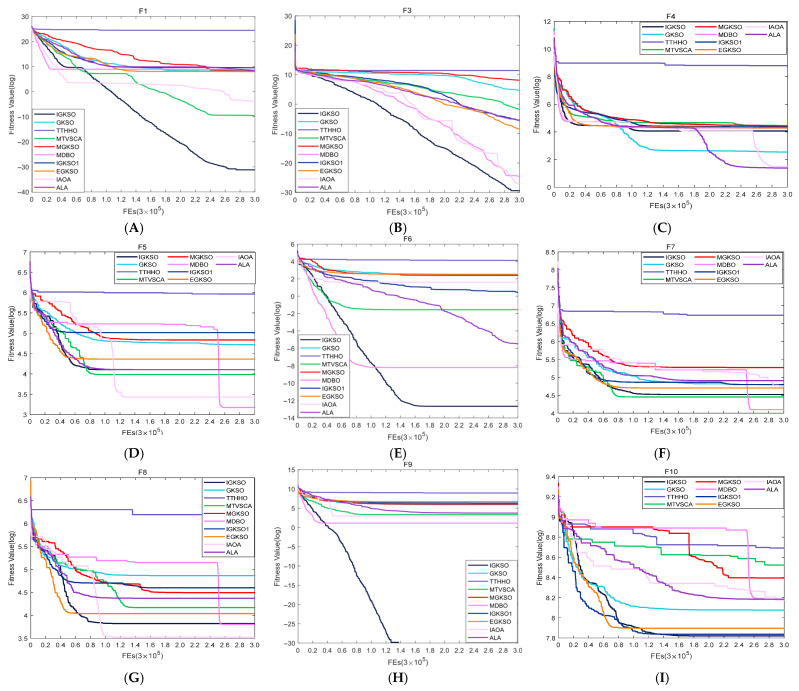

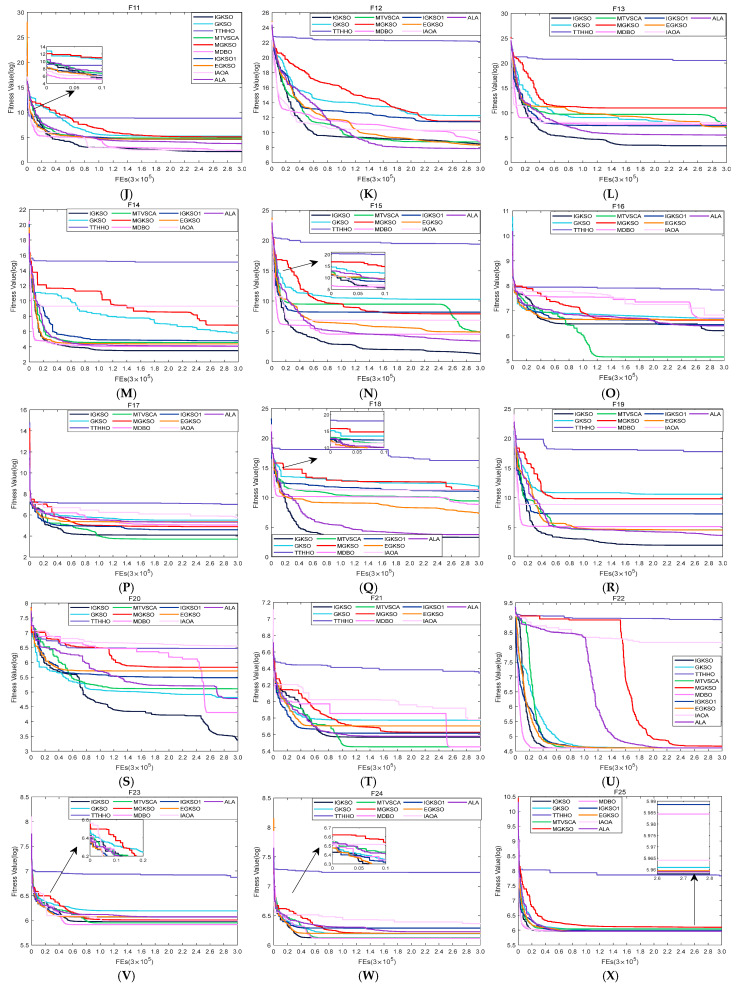

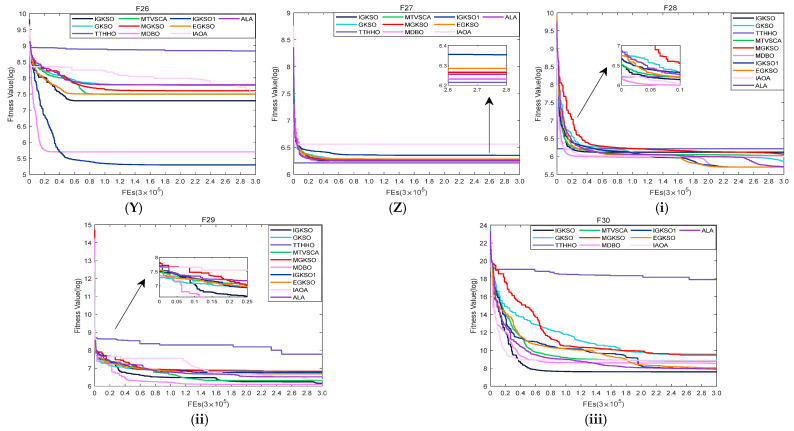
Convergence curves of the algorithms on the 30-dimensional CEC2017 test functions: (**A**) The convergence curve in F1. (**B**) The convergence curve in F3. (**C**) The convergence curve in F4. (**D**) The convergence curve in F5. (**E**) The convergence curve in F6. (**F**) The convergence curve in F7. (**G**) The convergence curve in F8. (**H**) The convergence curve in F9. (**I**) The convergence curve in F10. (**J**) The convergence curve in F11. (**K**) The convergence curve in F12. (**L**) The convergence curve in F13. (**M**) The convergence curve in F14. (**N**) The convergence curve in F15. (**O**) The convergence curve in F16. (**P**) The convergence curve in F17. (**Q**) The convergence curve in F18. (**R**) The convergence curve in F19. (**S**) The convergence curve in F20. (**T**) The convergence curve in F21. (**U**) The convergence curve in F22. (**V**) The convergence curve in F23. (**W**) The convergence curve in F24. (**X**) The convergence curve in F25. (**Y**) The convergence curve in F26. (**Z**) The convergence curve in F27. (**i**) The convergence curve in F28. (**ii**) The convergence curve in F29. (**iii**) The convergence curve in F30.

**Figure 4:**
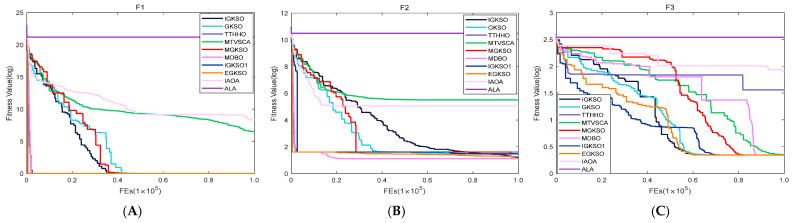

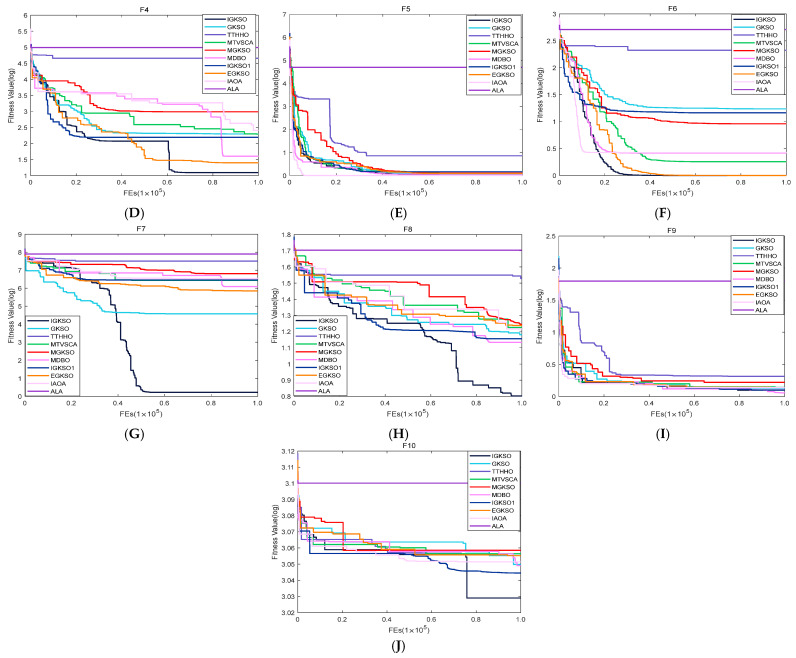
Convergence curves of the algorithms on the CEC2019 test functions: (**A**) The convergence curve in F1. (**B**) The convergence curve in F2. (**C**) The convergence curve in F3. (**D**) The convergence curve in F4. (**E**) The convergence curve in F5. (**F**) The convergence curve in F6. (**G**) The convergence curve in F7. (**H**) The convergence curve in F8. (**I**) The convergence curve in F9. (**J**) The convergence curve in F10.

For a more intuitive visualization of the disparities in convergence speed among the algorithms, Figure 3 and Figure 4 depict the convergence trajectories of all algorithms on the 30-dimensional CEC2017 and CEC2019 test suites, respectively. The abscissa indicates the number of function evaluations, whereas the ordinate represents the logarithmic fitness values.

Based on the convergence curves of all compared algorithms on the CEC2017 test suite, as shown in Figure 3, it can be observed that IGKSO achieves the global optimum on functions F1, F3, F6, F9, F10, F11, F13, F14, F15, F18, F19, F20, F25, and F30. A detailed analysis and summary are provided below in conjunction with the evolutionary process of each function.

For the unimodal functions F1 and F3, IGKSO demonstrates excellent global search capability and late-stage convergence performance. Specifically, in F1, the convergence speed of IGKSO is only slightly slower than that of MDBO and IAOA in the early evolutionary stage. Subsequently, all three algorithms fall into local optima, but only IGKSO successfully escapes after a period of search, ultimately ranking first in both convergence speed and convergence accuracy. In F3, the convergence speed of IGKSO is similar to that of the other compared algorithms in the early stage, showing no significant advantage. However, as the evolutionary process progresses, its convergence speed and accuracy gradually exhibit a clear advantage, eventually securing the top position. For the multimodal functions F4 to F10, the performance of IGKSO varies, reflecting its adaptability and competitiveness under different function characteristics. In F4, the convergence speed of IGKSO is only slightly slower than that of MDBO and IAOA in the early stage, after which all three fall into local optima. Near the middle stage, both IGKSO and IAOA show minor escapes, but in the later stage, IGKSO falls back into a local optimum and fails to escape, while IAOA successfully escapes. As a result, the final convergence accuracy of IGKSO is inferior to that of ALA, GKSO, and IAOA, ranking fourth. In F5, the convergence speed of IGKSO is only slightly slower than that of EGKSO in the early stage. Subsequently, both algorithms fall into local optima and fail to escape throughout the evolutionary process. Ultimately, IGKSO outperforms EGKSO in convergence accuracy and is close to ALA, but is inferior to MTVSCA, IAOA, and MDBO, also ranking fourth. In F6, the convergence speed of IGKSO is slower than that of MDBO and MTVSCA in the early stage. However, as evolution progresses, MDBO and MTVSCA successively fall into local optima, and IGKSO gradually surpasses them in convergence speed, finally achieving the global optimum in both convergence speed and accuracy. In F7, the convergence speed of IGKSO is among the forefront in the early stage, but later, like most algorithms, it falls into a local optimum, with final convergence accuracy only slightly inferior to MTVSCA and MDBO. In F8, the convergence speed of IGKSO is also among the forefront in the early stage and then gradually accelerates, being only slightly weaker than MGKSO. However, it subsequently falls into a local optimum and fails to escape, resulting in convergence accuracy inferior to MDBO and IAOA, ranking third. In F9, the convergence speed of IGKSO is slower than that of MDBO in the early stage. As evolution proceeds, MDBO falls into a local optimum, while IGKSO continues to optimize, ultimately achieving the global optimum in both convergence speed and accuracy. In F10, the convergence speed of IGKSO is slower than that of MTVSCA, IGKSO1, and EGKSO in the early stage. Subsequently, its convergence speed gradually increases and surpasses that of MTVSCA. Although all four algorithms eventually fall into local optima, IGKSO still achieves the global optimum in convergence accuracy.

For the hybrid functions F11 to F20, IGKSO achieves the global optimum on most functions, with only slight deficiencies in a few. In F11, the convergence speed of IGKSO is already among the forefront in the early stage, and it further accelerates to rank first, ultimately reaching the global optimum in convergence accuracy. In F12, the convergence speed of IGKSO is similar to that of MTVSCA in the early stage, only inferior to MDBO and IAOA. Subsequently, its convergence speed gradually surpasses these two algorithms, ranking first in convergence speed, but its final convergence accuracy is inferior to ALA and EGKSO, ranking third. In F13, the convergence speed of IGKSO is only inferior to MDBO and IAOA in the early stage, and it gradually accelerates, ultimately achieving the global optimum in both convergence speed and accuracy. In F14, the convergence speed of IGKSO is only inferior to MDBO and similar to EGKSO in the early stage. As time progresses, its convergence performance continuously improves, finally achieving the global optimum in convergence speed and accuracy. In F15, the convergence speed of IGKSO is only inferior to MDBO and similar to IAOA in the early stage. Subsequently, its convergence speed gradually accelerates and surpasses these two algorithms, ultimately achieving the global optimum. In F16, the convergence speed of IGKSO is among the forefront in the early stage and gradually accelerates to rank first, but it falls into a local optimum. Notably, in the later stage, IGKSO slightly escapes from the local optimum, and its convergence accuracy gradually surpasses that of other algorithms, ultimately ranking second, only inferior to MTVSCA. In F17, the convergence speed of IGKSO is also among the forefront in the early stage and gradually accelerates to rank first, but it falls into a local optimum and fails to escape, with final convergence accuracy also ranking second, only inferior to MTVSCA. In F18, the convergence speed of IGKSO is among the forefront in the early stage and gradually accelerates to rank first, ultimately achieving the global optimum in both convergence accuracy and speed. In F19, the convergence speed of IGKSO is similar to that of MTVSCA in the early stage, only inferior to MDBO and IAOA. Subsequently, its convergence speed gradually surpasses these two algorithms, finally achieving the global optimum. In F20, the convergence speed of IGKSO is only inferior to GKSO in the early stage, and it gradually surpasses GKSO, ultimately achieving the global optimum in convergence speed and accuracy. For the composite functions F21 to F30, although IGKSO does not achieve the optimum on most functions, its overall performance is stable, demonstrating strong competitiveness and robustness. In F21, the convergence speed of IGKSO is in the mid-to-late position in the early stage but is significantly stronger than that of IGKSO1, IAOA, and MGKSO. It gradually falls into a local optimum, and its final convergence accuracy is close to that of ALA, only inferior to MTVSCA and MDBO, ranking third. In F22, the convergence speed of IGKSO is in the middle position in the early stage, then gradually accelerates, being only inferior to MDBO. Its final convergence accuracy is better than that of MGKSO and is roughly on par with other algorithms. In F23, the convergence speed of IGKSO is also in the middle position in the early stage, after which it gradually falls into a local optimum. The final convergence accuracy of IGKSO is only inferior to MTVSCA and MDBO, ranking third. In F24, the convergence speed of IGKSO holds a clear advantage in the early stage, but eventually, like all other algorithms, it falls into a local optimum, with convergence accuracy close to that of MTVSCA and IAOA. In F25, the convergence speed of IGKSO also holds a clear advantage in the early stage. Although it falls into a local optimum along with other algorithms in the later stage, it still achieves the global optimum in convergence accuracy. In F26, the convergence speed of IGKSO has a clear advantage in the early stage, only inferior to IGKSO1 and MDBO. Subsequently, like most algorithms, it gradually falls into a local optimum, with final convergence accuracy ranking third, only inferior to the two aforementioned comparison algorithms. In F27, the convergence speed of IGKSO is in the middle position in the early stage, and then, like most algorithms, it falls into a local optimum, with final convergence accuracy ranking third, only inferior to TTHHO and MTVSCA. In F28, the convergence speed of IGKSO is among the forefront in the early stage, but it also falls into a local optimum later, with final convergence accuracy not showing a significant advantage. In F29, the convergence speed of IGKSO is among the forefront in the early stage, and it gradually accelerates, with final convergence accuracy ranking second, only inferior to MDBO. In F30, the convergence speed of IGKSO is among the forefront in the early stage, only slightly inferior to MDBO and IAOA. Subsequently, its convergence speed gradually accelerates and takes the lead, ultimately achieving the global optimum in both convergence speed and accuracy.

Based on the convergence curve analysis of the CEC2019 test suite presented in Figure 4, the IGKSO algorithm exhibits diverse convergence characteristics across different functions, collectively demonstrating strong late-stage acceleration capability and excellent global search performance. On function F1, IGKSO’s initial convergence speed is most similar to that of MTV-SCA, only outperforming ALA, IAOA, and MGKSO. However, as the evolution progresses, its convergence speed gradually surpasses that of ALA, IAOA, MTV-SCA, GKSO, and MGKSO. Ultimately, its final convergence accuracy is higher than that of ALA, IAOA, and MTV-SCA, while remaining comparable to the other algorithms. On function F2, IGKSO’s convergence speed is relatively slow in the early evolutionary stage, lagging behind other compared algorithms. Upon entering the later evolutionary stage, however, its convergence speed accelerates significantly, enabling it to continuously search for better solutions, while the other algorithms become trapped in local optima, leading to degraded search performance. Consequently, IGKSO achieves a convergence accuracy second only to MDBO. On function F3, IGKSO’s convergence speed is positioned in the middle during the early evolutionary phase, inferior only to GKSO, EGKSO, TTHHO, and IGKSO1. As time progresses, its convergence speed gradually rises to the top, ultimately achieving convergence accuracy significantly better than that of IAOA, ALA, and TTHHO, while remaining comparable to the other algorithms. On function F4, IGKSO initially holds a distinct advantage over the other algorithms in both convergence accuracy and speed. Subsequently, IGKSO1’s convergence speed temporarily overtakes that of IGKSO. However, as evolution proceeds, IGKSO gradually reclaims the leading position, ultimately ranking first in both convergence accuracy and speed. On function F5, IGKSO’s convergence speed is at a moderate level in the early evolutionary phase, inferior only to IGKSO1, IAOA, and MDBO. It maintains a position among the top tier during the middle phase, but all algorithms become trapped in local optima, unable to conduct further exploration. The final results indicate that IGKSO’s performance is largely comparable to that of the other algorithms, with the exception of ALA and TTHHO. On function F6, IGKSO’s convergence speed is second only to IAOA in the early evolutionary stage, and it progressively converges to the global optimum by the mid-to-early phase. On function F7, IGKSO’s convergence speed is at a moderate level in the early evolutionary stage. As evolution advances, its convergence speed gradually increases, and as it approaches the middle stage, both its convergence speed and accuracy improve dramatically. Ultimately, IGKSO ranks first in both convergence speed and accuracy. On function F8, IGKSO’s convergence speed is among the highest in the early evolutionary phase, second only to IGKDO1 and MDBO. Over time, it catches up with and gradually surpasses IGKSO1 in terms of convergence speed, finally achieving global optimality in both convergence speed and accuracy. On function F9, although IGKSO does not hold an advantage in convergence speed, the difference compared to other algorithms is minimal. Moreover, its convergence accuracy is second only to MDBO. On function F10, IGKSO’s convergence speed is only slightly inferior to that of IGKSO1 in the early evolutionary stage. During the middle and later stages, it gradually takes the lead in terms of both convergence speed and accuracy. In summary, the IGKSO algorithm exhibits remarkable late-stage convergence capability and global search performance across most functions, maintaining search vitality when other algorithms become trapped in local optima, and ultimately achieving convergence accuracy that is generally superior to or closely approaching the best-performing algorithms among those compared.

In conclusion, compared with the nine other approaches, IGKSO demonstrates certain advantages regarding convergence speed on both the CEC2017 and CEC2019 test suites, ultimately achieving competitive performance in terms of both convergence speed and accuracy.

### 4.4. Application to Photovoltaic Model Parameter Extraction

**Figure 5:**
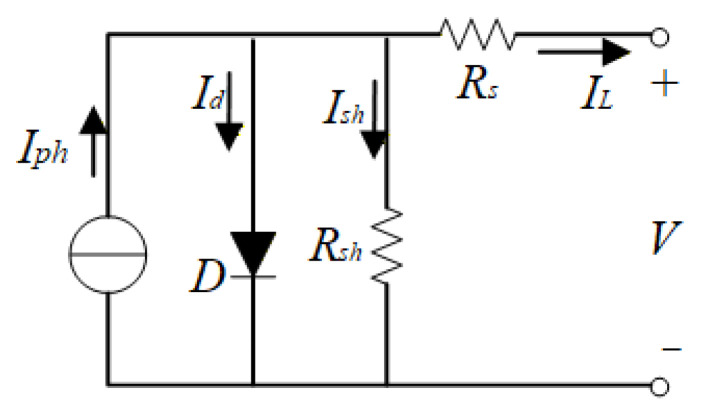
Single-diode photovoltaic model.

To verify the capability of the IGKSO algorithm in solving practical engineering optimization problems, this section applies it to a typical photovoltaic model parameter extraction problem. Photovoltaic (PV) model parameter extraction [31] is a fundamental core technology in PV system modeling, performance evaluation, and optimization control. Whether in scenarios such as power output prediction of PV power plants, fault diagnosis of PV modules, or maximum power point tracking and system optimization operation, it is through accurate parameter extraction that the current–voltage characteristics of PV cells are precisely characterized, which holds significant engineering value and broad application demand. In this section, the typical single-diode photovoltaic model (SDM) is adopted as the research model, and the root mean square error (RMSE) between the measured current and the experimental current is used as the objective function, as given in Equation (45). The goal is to find a set of optimal parameters that minimize the objective function. The single-diode photovoltaic model is shown in Figure 5, and its objective function is Equation (46).

(45) $$RMSE=\sqrt{\frac{1}{N}\sum_{i=1}^{N}f(V,I_{L},X)}$$

(46) $$\left\{\begin{array}{c} f(V,I_{L},x)=I_{ph}-I_{0}\cdot [\exp(\frac{Q\cdot (V+R_{s}\cdot I_{L})}{\eta \cdot \alpha \cdot T})-1]-\frac{V+R_{s}\cdot I_{L}}{R_{sh}}-I_{L}\\ x=\{I_{ph},I_{0},\alpha ,R_{sh},R_{s}\} \end{array}\right.$$

Here, $I_{L}$, $I_{ph}$, $I_{d}$ and $I_{sh}$ represent the output current of the cell, the photogenerated current, the diode current, and the shunt resistance current, respectively; $I_{0}$ is the reverse saturation current of the diode; *V* is the output voltage; $\eta =1.3806503\times 10^{-23}$ J/K is the Boltzmann constant; $Q=1.60217646\times 10^{-19}$ is the electron charge; α is the ideality factor of the diode; *T* is the temperature of the PV cell; $R_{s}$ and $R_{sh}$ are the series resistance and shunt resistance, respectively. Thus, in the single-diode model, five unknown parameters ($I_{ph}$, $I_{0}$, α, $R_{sh}$, $R_{s}$) need to be estimated.

**Figure 6:**
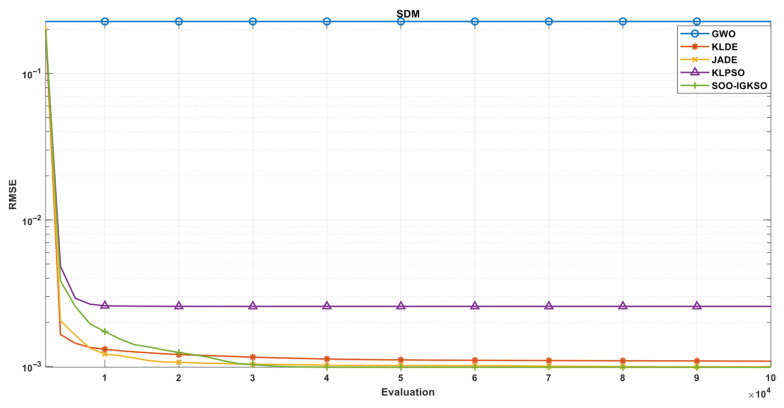
Convergence curve of RMSE on SDM.

To verify the effectiveness of the proposed IGKSO algorithm for the diode photovoltaic model optimization problem, we compared it with four other state-of-the-art evolutionary algorithms currently used for this application, including JADE, KLDE, KLPSO and GWO. The specific parameter settings are shown in Table 24, which are consistent with those in the original reference. In the experiments, each algorithm was independently run 10 times, with a population size of 50 and a maximum number of function evaluations of 100,000. The termination condition for all algorithms was the maximum number of function evaluations. The specific settings of the single-diode photovoltaic model can be found in Reference [30]. The detailed experimental results are presented in Table 25, and the corresponding convergence curves are shown in Figure 6.

According to the results shown in Table 25, the RMSE value obtained by IGKSO in the single-diode parameter extraction problem is significantly lower than those of the other comparison algorithms, demonstrating superior optimization performance. As shown in the convergence curves in Figure 6, IGKSO outperforms the other algorithms in terms of both convergence accuracy and stability. In summary, IGKSO not only performs excellently on benchmark test functions but also exhibits outstanding solution capability and practical value in real-world engineering optimization problems such as photovoltaic model parameter extraction.

## 5. Conclusions

This study proposes an Improved Genghis Khan shark optimization (IGKSO) algorithm, designed to overcome the limitations of the original algorithm, such as insufficient population diversity and a tendency toward premature convergence. First, an opposition-based learning mechanism is introduced in the hunting phase to generate oppositional solutions, thereby enhancing population diversity and effectively reducing the risk of falling into local suboptimal solutions. Second, a dual-factor population partitioning strategy based on fitness–cosine similarity is developed, dividing the population into dominant and non-dominant subpopulations according to individual quality and distribution characteristics. These subpopulations are employed in different search stages: the moving phase utilizes the dominant subpopulation for refined exploitation, while the parabolic foraging phase leverages the non-dominant subpopulation to maintain exploration capability. In the moving phase, a multi-candidate basis vector random selection mechanism is proposed, where the basis vector is randomly selected from four candidate individuals: two offspring generated by fusing the best individual and the fittest dissimilar individual via simulated binary crossover, the best individual, and the current individual. The first two candidates introduce stochasticity through crossover to preserve diversity, while the latter two facilitate convergence acceleration. Additionally, the step size mechanism in this phase employs fitness normalization to effectively prevent search boundary violations. In the foraging phase, a subspace pattern is introduced to avoid premature population convergence, reducing computational overhead while mitigating diversity loss. Moreover, dynamic coordination between exploration and exploitation is achieved through adaptive adjustment parameters placed before two difference vectors. Finally, in the self-protection phase, the ratio of the population’s spatial hypervolume to the overall problem space hypervolume is used to quantitatively assess the population’s clustering degree in real time, thereby measuring diversity levels and determining whether to enhance convergence speed or supplement diversity. However, the algorithm’s performance in handling high-dimensional complex optimization problems still requires further validation. Future research will focus on scalability in high-dimensional problems, theoretical analysis of convergence guarantees for the IGKSO algorithm, and a more comprehensive investigation into the time complexity of the comparison algorithms to further assess their computational efficiency.

## Figures and Tables

**Table 1:** Nemenyi post hoc test results.

Comparison	*p*-Value (CEC2017)	*p*-Value (CEC2019)
IGKSO vs. MDBO	0.0414	0.0548
IGKSO vs. ALA	0.0043	0.0157
IGKSO vs. MTV-SCA	0.0041	0.0153
IGKSO vs. EGKSO	0.0002	0.0061
IGKSO vs. IAOA	0.0000	0.0040
IGKSO vs. IGKSO1	0.0000	0.0015
IGKSO vs. GKSO	0.0000	0.0000
IGKSO vs. MGKSO	0.0000	0.0000
IGKSO vs. TTHHO	0.0000	0.0000

**Table 2:** Wilcoxon rank-sum test results for IGKSO.

Comparison	+/=/− (CEC2017)	+/=/− (CEC2019)
IGKSO vs. MDBO	9/6/14	0/6/4
IGKSO vs. ALA	2/3/24	0/0/10
IGKSO vs. MTV-SCA	0/10/19	1/0/9
IGKSO vs. EGKSO	0/4/25	0/4/6
IGKSO vs. IAOA	6/2/21	2/1/7
IGKSO vs. IGKSO1	2/3/24	1/3/6
IGKSO vs. GKSO	0/2/27	0/2/8
IGKSO vs. MGKSO	0/0/29	0/1/9
IGKSO vs. TTHHO	1/0/28	0/1/9

**Table 3:** Parameter description.

Parameter	Definition
*s*	Olfactory intensity toward the optimal prey, which is specifically defined by Equation (3)
*I*	Each individual’s objective function value (i.e., the raw, unnormalized fitness value)
*p*	Movement step size, which is specifically defined by Equation (5)
*N*_1_	The size of the superior population, which is specifically defined by Equation (17)
*N*_2_	The size of dissimilar individuals, which is specifically defined by Equation (18)
*s*_1_	Adaptive movement step size, which is specifically defined by Equation (20) (the improved version of parameter *s*)
*fit_max*, *fit_min*	Maximum fitness and minimum fitness
*X_k_*	It is specifically defined by Equation (11)
$X_{best\_sbx}$ $X_{dis\_sbx}$	Two individuals generated by *SBX* between the optimal individual and the individual with the best fitness among dissimilar individuals
*z*	Subspace mode
*p*_1_	Movement step size, *p*_1_ = *p*/2 (the improved version of parameter *p*)
*r*_21_	Adaptive coefficient, which is specifically defined by Equation (26) (the improved version of parameter *r*_2_)
*LIM*	Diversity threshold
*X*_u1_, *X*_u2_	Two random individuals in the whole population
*X*2, *X*1	Two individuals generated by random initialization
*a*_1_, *a*_2_, *a*_3_	stochastic coefficients, which is specifically defined by Equations (8)–(10)
*k*_2_	Standard normal distribution
*X_r_*_1_, *X_r_*_2_	Two random individuals in the whole population
$X_{dis}$	The individual with the best fitness among dissimilar individuals
*k*_4_	Normal distribution, whose standard deviation is determined by Equation (32) (the improved version of parameter *k*_2_)
*a*_4_, *a*_5_, *a*_6_, *a*_7_	Adaptive coefficients, which is specifically defined by Equations (34)–(37) (the improved version of parameter *a*_1_, *a*_2_, *a*_3_)
*X_k_*_1_	It is specifically defined by Equation (30) (the improved version of parameter *X_k_*)
*l_x_*	Upper limit of population
*u_x_*	Lower limit of population
*k*_1_	Random number between [0, 1]
*k*_3_	Random number, which is specifically defined by Equation (33) (the improved version of parameter *k*_1_)

**Table 4:** Parameter analysis on *k* and *n*.

Function		*k* = 50	*k* = 100	*k* = 200	*n* = 13	*n* = 15	*n* = 20
F1	Mean Std	0.00 × 10^0^ (=) 0.00 × 10^0^	0.00 × 10^0^ 0.00 × 10^0^	0.00 × 10^0^ (=) 0.00 × 10^0^	0.00 × 10^0^ (=) **0.00 × 10^0^**	0.00 × 10^0^ 0.00 × 10^0^	0.00 × 10^0^ (=) 0.00 × 10^0^
F2	Mean Std	5.57 × 10^−1^ (=) 5.37 × 10^−1^	**4.03 × 10^−1^** **4.93 × 10^−1^**	4.17 × 10^−1^ (=) 5.14 × 10^−1^	4.37 × 10^−1^ (=) 5.90 × 10^−1^	4.03 × 10^−1^ 4.93 × 10^−1^	**3.94 × 10^−1^** (=) **4.87 × 10^−1^**
F3	Mean Std	6.54 × 10^−1^ (=) 1.19 × 10^0^	**3.32 × 10^−1^** 1.72 × 10^−1^	3.89 × 10^−1^ (=) **9.15 × 10^−2^**	3.89 × 10^−1^ (=) **9.15 × 10^−2^**	**3.32 × 10^−1^** 1.72 × 10^−1^	3.50 × 10^−1^ (=) 1.51 × 10^−1^
F4	Mean Std	6.42 × 10^0^ (−) **2.13 × 10^0^**	**4.83 × 10^0^** 2.58 × 10^0^	5.47 × 10^0^ (=) 2.25 × 10^0^	5.49 × 10^0^ (=) 2.46 × 10^0^	**4.83 × 10^0^** 2.58 × 10^0^	5.72 × 10^0^ (=) **2.01 × 10^0^**
F5	Mean Std	8.40 × 10^−2^ (=) 3.38 × 10^−2^	**5.09 × 10^−2^** 3.46 × 10^−2^	6.05 × 10^−2^ (=) **3.13 × 10^−2^**	8.31 × 10^−2^ (−) 4.88 × 10^−2^	**5.09 × 10^−2^** 3.46 × 10^−2^	6.46 × 10^−2^ (=) **3.27 × 10^−2^**
F6	Mean Std	9.33 × 10^−15^ (=) 3.27 × 10^−14^	0.00 × 10^0^ 0.00 × 10^0^	1.60 × 10^−2^ (=) 7.16 × 10^−2^	2.35 × 10^−14^ (=) 8.36 × 10^−14^	0.00 × 10^0^ 0.00 × 10^0^	3.49 × 10^−14^ (=) 1.25 × 10^−13^
F7	Mean Std	1.77 × 10^2^ (=) 2.07 × 10^2^	**6.79 × 10^1^** **1.14 × 10^2^**	1.27 × 10^2^ (=) 1.34 × 10^2^	1.28 × 10^2^ (=) 1.42 × 10^2^	**6.79 × 10^1^** **1.14 × 10^2^**	1.28 × 10^2^ (=) 1.52 × 10^2^
F8	Mean Std	2.06 × 10^0^ (=) 3.68 × 10^−1^	**1.71 × 10^0^** 5.37 × 10^−1^	2.08 × 10^0^ (−) **3.55 × 10^−1^**	1.78 × 10^0^ (=) **4.19 × 10^−1^**	**1.71 × 10^0^** 5.37 × 10^−1^	1.98 × 10^0^ (=) 4.38 × 10^−1^
F9	Mean Std	1.24 × 10^−1^ (=) **3.66 × 10^−2^**	**1.09 × 10^−1^** 4.07 × 10^−2^	1.15 × 10^−1^ (=) 4.08 × 10^−2^	**1.19 × 10^−1^** (=) 4.01 × 10^−2^	**1.09 × 10^−1^** 4.07 × 10^−2^	1.41 × 10^−1^ (−) **3.78 × 10^−2^**
F10	Mean Std	2.01 × 10^1^ (=) **8.10 × 10^−2^**	**1.54 × 10^1^** 8.51 × 10^0^	1.72 × 10^1^ (=) 7.25 × 10^0^	2.01 × 10^1^ (=) **6.71 × 10^−2^**	**1.54 × 10^1^** 8.51 × 10^0^	1.82 × 10^1^ (=) 6.02 × 10^0^
(+/−/=)		0/1/9	**Base**	0/1/9	0/1/9	**Base**	0/1/9

**Table 5:** Parameter analysis on *LIM*.

Function		*LIM* = 0.05	*LIM* = 0.10	*LIM* = 0.15	*LIM* = 0.20
F1	Mean Std	0.00 × 10^0^ (=) 0.00 × 10^0^	0.00 × 10^0^ (=) 0.00 × 10^0^	0.00 × 10^0^ 0.00 × 10^0^	0.00 × 10^0^ (=) 0.00 × 10^0^
F2	Mean Std	8.19 × 10^−1^ (=) 9.44 × 10^−1^	8.13 × 10^−1^ (=) 9.16 × 10^−1^	**4.03 × 10^−1^** **4.93 × 10^−1^**	7.26 × 10^−1^ (=) 5.09 × 10^−1^
F3	Mean Std	3.89 × 10^−1^ (=) 9.15 × 10^−2^	**3.27 × 10^−1^** (=) 1.68 × 10^−1^	3.32 × 10^−1^ 1.72 × 10^−1^	4.09 × 10^−1^ (=) **9.82 × 10^−5^**
F4	Mean Std	7.51 × 10^0^ (−) 2.79 × 10^0^	5.37 × 10^0^ (=) 2.22 × 10^0^	**4.83 × 10^0^** 2.58 × 10^0^	5.62 × 10^0^ (=) **2.12 × 10^0^**
F5	Mean Std	6.18 × 10^−2^ (=) 3.76 × 10^−2^	6.19 × 10^−2^ (=) 3.69 × 10^−2^	**5.09 × 10^−2^** 3.46 × 10^−2^	7.38 × 10^−2^ (=) **3.26 × 10^−2^**
F6	Mean Std	9.39 × 10^−2^ (=) 4.20 × 10^−1^	2.63 × 10^−2^ (=) 1.03 × 10^−1^	0.00 × 10^0^ 0.00 × 10^0^	4.63 × 10^−2^ (=) 1.42 × 10^−1^
F7	Mean Std	1.46 × 10^2^ (=) 1.92 × 10^2^	1.36 × 10^2^ (=) 1.49 × 10^2^	**6.79 × 10^1^** **1.14 × 10^2^**	1.88 × 10^2^ (=) 1.61 × 10^2^
F8	Mean Std	1.72 × 10^0^ (=) 5.27 × 10^−1^	1.82 × 10^0^ (=) **2.99 × 10^−1^**	**1.71 × 10^0^** 5.37 × 10^−1^	2.00 × 10^0^ (=) 3.82 × 10^−1^
F9	Mean Std	1.13 × 10^−1^ (=) 4.04 × 10^−2^	1.30 × 10^−1^ (=) **3.66 × 10^−2^**	**1.09 × 10^−1^** 4.07 × 10^−2^	1.21 × 10^−1^ (=) 3.73 × 10^−2^
F10	Mean Std	1.91 × 10^1^ (=) **4.50 × 10^0^**	1.91 × 101 (=) 4.59 × 10^0^	**1.54 × 10^1^** 8.51 × 10^0^	1.81 × 10^1^ (=) 6.19 × 10^0^
(+/−/=)		0/1/9	0/0/10	**Base**	0/0/10

**Table 6:** Parameter analysis on *η*.

Function		*η* = 1	*η* = 2	*η* = 5	*η* = 10	*η* = 20
F1	Mean Std	0.00 × 10^0^ 0.00 × 10^0^	0.00 × 10^0^ (=) 0.00 × 10^0^	0.00 × 10^0^ (=) 0.00 × 10^0^	0.00 × 10^0^ (=) 0.00 × 10^0^	0.00 × 10^0^ (=) 0.00 × 10^0^
F2	Mean Std	**4.03 × 10^−1^** **4.93 × 10^−1^**	8.37 × 10^−1^ (=) 1.09 × 10^0^	4.81 × 10^−1^ (=) 5.01 × 10^−1^	7.04 × 10^−1^ (=) 6.45 × 10^−1^	8.54 × 10^−1^ (=) 9.55 × 10^−1^
F3	Mean Std	**3.32 × 10^−1^** 1.72 × 10^−1^	3.68 × 10^−1^ (=) 1.26 × 10^−1^	3.89 × 10^−1^ (=) 9.15 × 10^−2^	3.89 × 10^−1^ (=) 9.15 × 10^−2^	4.09 × 10^−1^ (=) **5.34 × 10^−4^**
F4	Mean Std	**4.83 × 10^0^** 2.58 × 10^0^	5.62 × 10^0^ (=) 2.63 × 10^0^	6.02 × 10^0^ (=) 2.25 × 10^0^	5.62 × 10^0^ (=) **2.02 × 10^0^**	5.37 × 10^0^ (=) 2.13 × 10^0^
F5	Mean Std	**5.09 × 10^−2^** 3.46 × 10^−2^	6.55 × 10^−2^ (=) 4.07 × 10^−2^	7.21 × 10^−2^ (=) 4.28 × 10^−2^	7.01 × 10^−2^ (=) **3.10 × 10^−2^**	7.80 × 10^−2^ (=) 4.01 × 10^−2^
F6	Mean Std	0.00 × 10^0^ **0.00 × 10^0^**	1.23 × 10^−12^ (=) 3.90 × 10^−12^	3.74 × 10^−2^ (−) 1.19 × 10^−1^	2.30 × 10^−2^ (=) 1.03 × 10^−1^	4.63 × 10^−2^ (=) 1.42 × 10^−1^
F7	Mean Std	**6.79 × 10^1^** **1.14 × 10^2^**	2.07 × 10^2^ (=) 1.87 × 10^2^	1.92 × 10^2^ (=) 1.79 × 10^2^	1.64 × 10^2^ (=) 1.68 × 10^2^	1.72 × 10^2^ (=) 1.44 × 10^2^
F8	Mean Std	1.71 × 10^0^ 5.37 × 10^−1^	2.04 × 10^0^ (=) 4.72 × 10^−1^	1.73 × 10^0^ (=) 4.35 × 10^−1^	1.69 × 10^0^ (+) **4.22 × 10^−1^**	**1.68 × 10^0^** (+) 4.41 × 10^−1^
F9	Mean Std	**1.09 × 10^−1^** **4.07 × 10^−2^**	1.34 × 10^−1^ (=) 4.91 × 10^−2^	1.19 × 10^−1^ (=) 4.76 × 10^−2^	1.12 × 10^−1^ (=) 4.51 × 10^−2^	1.10 × 10^−1^ (=) 4.26 × 10^−2^
F10	Mean Std	**1.54 × 10^1^** 8.51 × 10^0^	1.71 × 10^1^ (=) 7.38 × 10^0^	1.81 × 10^1^ (=) 6.19 × 10^0^	1.71 × 10^1^ 7.38 × 10^0^ (=)	1.92 × 10^1^ (=) **4.51 × 10^0^**
(+/−/=)		**Base**	0/0/10	0/1/9	1/0/9	1/0/9

**Table 7:** Parameter analysis on *r*_21_.

	*r*_21_	[1.2–0.2] [0.2–0.4]	[1.2–0.2] [0.2–0.5]	[1.2–0.2] [0.2–0.6]	[1.1–0.1] [0.1–0.4]	[1.1–0.1] [0.1–0.5]	[1.1–0.1] [0.1–0.6]
Function							
F1	Mean Std	**0.00 × 10^0^** (=) **0.00 × 10^0^**	**0.00 × 10** **^0^** **0.00 × 10** **^0^**	**0.00 × 10^0^** (=) **0.00 × 10^0^**	**0.00 × 10^0^** (=) **0.00 × 10^0^**	**0.00 × 10^0^** (=) **0.00 × 10^0^**	**0.00 × 10^0^** (=) **0.00 × 10^0^**
F2	Mean Std	7.56 × 10^−1^ (=) 1.14 × 10^0^	**4.03 × 10^−1^** **4.93 × 10^−1^**	8.14 × 10^−1^ (=) 1.27 × 10^0^	5.93 × 10^−1^ (=) 6.12 × 10^−1^	9.94 × 10^−1^ (=) 1.34 × 10^0^	6.59 × 10^−1^ (=) 7.02 × 10^−1^
F3	Mean Std	4.09 × 10^−1^ (=) **2.93 × 10^−7^**	**3.32 × 10^−1^** 1.72 × 10^−1^	3.90 × 10^−1^ (=) 9.20 × 10^−2^	4.09 × 10^−1^ (=) 4.68 × 10^−6^	3.48 × 10^−1^ (=) 1.50 × 10^−1^	3.96 × 10^−1^ (=) 9.87 × 10^−2^
F4	Mean Std	5.72 × 10^0^ (=) **1.96 × 10^0^**	**4.83 × 10^0^** 2.58 × 10^0^	5.57 × 10^0^ (−) 2.15 × 10^0^	6.52 × 10^0^ (−) 2.88 × 10^0^	7.56 × 10^0^ (−) 2.47 × 10^0^	7.01 × 10^0^ (−) 2.98 × 10^0^
F5	Mean Std	5.86 × 10^−2^ (=) 3.54 × 10^−2^	**5.09 × 10^−2^** 3.46 × 10^−2^	6.21 × 10^−2^ (=) **2.41 × 10^−2^**	7.35 × 10^−2^ (−) 2.93 × 10^−2^	8.33 × 10^−2^ (=) 3.51 × 10^−2^	6.43 × 10^−2^ (=) 4.11 × 10^−2^
F6	Mean Std	4.63 × 10^−2^ (=) 1.42 × 10^−1^	**0.00 × 10^0^** **0.00 × 10^0^**	1.13 × 10^−13^ (=) 4.17 × 10^−13^	3.29 × 10^−3^ (=) 1.47 × 10^−2^	2.33 × 10^−2^ (=) 1.04 × 10^−1^	2.30 × 10^−2^ (=) 1.03 × 10^−1^
F7	Mean Std	1.58 × 10^2^ (−) 1.37 × 10^2^	**6.79 × 10^1^** **1.14 × 10^2^**	1.18 × 10^2^ (=) 1.56 × 10^2^	2.62 × 10^2^ (−) 1.94 × 10^2^	2.32 × 10^2^ (−) 2.17 × 10^2^	2.89 × 10^2^ (−) 2.21 × 10^2^
F8	Mean Std	2.13 × 10^0^ (−) **3.76 × 10^−1^**	**1.71 × 10^0^** 5.37 × 10^−1^	1.78 × 10^0^ (=) 4.56 × 10^−1^	1.75 × 10^0^ (=) 5.73 × 10^−1^	1.76 × 10^0^ (=) 6.23 × 10^−1^	1.78 × 10^0^ (=) 5.66 × 10^−1^
F9	Mean Std	1.16 × 10^−1^ (=) 4.98 × 10^−2^	**1.09 × 10^−1^** 4.07 × 10^−2^	1.24 × 10^−1^ (=) 3.49 × 10^−2^	1.31 × 10^−1^ (=) 3.59 × 10^−2^	1.11 × 10^−1^ (=) 3.90 × 10^−2^	1.22 × 10^−1^ (=) **2.98 × 10^−2^**
F10	Mean Std	1.92 × 10^1^ (=) **4.44 × 10^0^**	**1.54 × 10^1^** 8.51 × 10^0^	1.82 × 10^1^ (=) 6.03 × 10^0^	1.81 × 10^1^ (=) 6.21 × 10^0^	1.71 × 10^1^ (=) 7.38 × 10^0^	1.81 × 10^1^ (=) 6.19 × 10^0^
(+/−/=)		0/2/8	**Base**	0/1/9	0/3/7	0/2/8	0/2/8

**Table 8:** Parameter analysis on *n* and *N*_max_ on the 10-dimensional CEC2017 test set.

		*n* = 13			*n* = 15			*n* = 20		
Function		*N_max_* = 10	*N_max_* = 9	*N_max_* = 11	*N_max_* = 10	*N_max_* = 9	*N_max_* = 11	*N_max_* = 10	*N_max_* = 9	*N_max_* = 11
F1	mean	**0.00 × 10^0^**	6.25 × 10^−14^	6.11 × 10^−14^	**0.00 × 10^0^**	**0.00 × 10^0^**	7.39 × 10^−14^	**0.00 × 10^0^**	5.26 × 10^−14^	8.81 × 10^−14^
	std	**0.00 × 10^0^**	3.36 × 10^−14^	2.02 × 10^−14^	**0.00 × 10^0^**	**0.00 × 10^0^**	8.70 × 10^−14^	**0.00 × 10^0^**	2.49 × 10^−14^	8.09 × 10^−14^
F3	mean	**0.00 × 10^0^**	1.76 × 10^−13^	1.36 × 10^−13^	**0.00 × 10^0^**	**0.00 × 10^0^**	1.53 × 10^−13^	**0.00 × 10^0^**	1.51 × 10^−13^	1.48 × 10^−13^
	std	**0.00 × 10^0^**	6.81 × 10^−14^	4.79 × 10^−14^	**0.00 × 10^0^**	**0.00 × 10^0^**	4.68 × 10^−14^	**0.00 × 10^0^**	5.31 × 10^−14^	3.97 × 10^−14^
F4	mean	**0.00 × 10^0^**	5.42 × 10^1^	4.29 × 10^1^	**0.00 × 10^0^**	**0.00 × 10^0^**	3.82 × 10^1^	**0.00 × 10^0^**	3.60 × 10^1^	4.21 × 10^1^
	std	**0.00 × 10^0^**	1.78 × 10^1^	2.79 × 10^1^	**0.00 × 10^0^**	**0.00 × 10^0^**	3.12 × 10^1^	**0.00 × 10^0^**	3.02 × 10^1^	2.91 × 10^1^
F5	mean	4.38 × 10^0^	6.42 × 10^1^	5.57 × 10^1^	**4.28 × 10^0^**	4.93 × 10^0^	4.83 × 10^1^	4.78 × 10^0^	4.67 × 10^1^	4.88 × 10^1^
	std	3.48 × 10^0^	2.16 × 10^1^	2.11 × 10^1^	**1.63 × 10^0^**	2.08 × 10^0^	9.19 × 10^0^	2.32 × 10^0^	1.09 × 10^1^	1.25 × 10^1^
F6	mean	**0.00 × 10^0^**	3.08 × 10^−6^	2.14 × 10^−5^	**0.00 × 10^0^**	**0.00 × 10^0^**	1.79 × 10^−5^	**0.00 × 10^0^**	4.26 × 10^−6^	4.42 × 10^−6^
	std	**0.00 × 10^0^**	4.60 × 10^−6^	4.78 × 10^−5^	**0.00 × 10^0^**	**0.00 × 10^0^**	3.83 × 10^−5^	**0.00 × 10^0^**	6.11 × 10^−6^	5.56 × 10^−6^
F7	mean	1.51 × 10^1^	8.92 × 10^1^	8.08 × 10^1^	1.46 × 10^1^	1.48 × 10^1^	8.16 × 10^1^	**1.44 × 10^1^**	8.36 × 10^1^	8.45 × 10^1^
	std	2.02 × 10^0^	1.60 × 10^1^	1.94 × 10^1^	2.48 × 10^0^	2.80 × 10^0^	1.77 × 10^1^	**1.98 × 10^0^**	1.51 × 10^1^	9.06 × 10^0^
F8	mean	4.58 × 10^0^	4.71 × 10^1^	5.06 × 10^1^	5.77 × 10^0^	5.37 × 10^0^	5.15 × 10^1^	**4.43 × 10^0^**	4.84 × 10^1^	5.49 × 10^1^
	std	2.31 × 10^0^	1.38 × 10^1^	1.53 × 10^1^	2.34 × 10^0^	2.67 × 10^0^	1.06 × 10^1^	**2.15 × 10^0^**	1.82 × 10^1^	1.13 × 10^1^
F9	mean	**0.00 × 10^0^**	7.23 × 10^−2^	1.91 × 10^−1^	**0.00 × 10^0^**	**0.00 × 10^0^**	2.00 × 10^−1^	**0.00 × 10^0^**	7.26 × 10^−2^	8.95 × 10^−3^
	std	**0.00 × 10^0^**	1.70 × 10^−1^	3.41 × 10^−1^	**0.00 × 10^0^**	**0.00 × 10^0^**	5.70 × 10^−1^	**0.00 × 10^0^**	2.22 × 10^−1^	2.83 × 10^−2^
F10	mean	**7.26 × 10^1^**	2.89 × 10^3^	2.74 × 10^3^	2.00 × 10^2^	1.58 × 10^2^	3.10 × 10^3^	1.62 × 10^2^	2.98 × 10^3^	2.98 × 10^3^
	std	**7.98 × 10^1^**	5.56 × 10^2^	6.48 × 10^2^	1.53 × 10^2^	1.33 × 10^2^	6.70 × 10^2^	1.49 × 10^2^	5.07 × 10^2^	6.22 × 10^2^
F11	mean	**0.00 × 10^0^**	1.31 × 10^1^	1.15 × 10^1^	1.99 × 10^−1^	4.97 × 10^−2^	1.25 × 10^1^	1.99 × 10^−1^	1.31 × 10^1^	1.28 × 10^1^
	std	**0.00 × 10^0^**	4.03 × 10^0^	4.62 × 10^0^	4.20 × 10^−1^	2.22 × 10^−1^	5.75 × 10^0^	4.08 × 10^−1^	4.46 × 10^0^	5.05 × 10^0^
F12	mean	**2.29 × 10^−1^**	8.66 × 10^3^	2.84 × 10^3^	1.37 × 10^0^	8.08 × 10^−1^	4.52 × 10^3^	2.71 × 10^−1^	2.71 × 10^3^	5.94 × 10^3^
	std	**1.54 × 10^−1^**	9.97 × 10^3^	3.86 × 10^3^	3.52 × 10^0^	2.45 × 10^0^	6.80 × 10^3^	1.92 × 10^−1^	3.30 × 10^3^	7.76 × 10^3^
F13	mean	2.42 × 10^0^	2.97 × 10^1^	2.99 × 10^1^	**9.95 × 10^−2^**	1.68 × 10^0^	2.86 × 10^1^	1.54 × 10^0^	2.50 × 10^1^	3.35 × 10^1^
	std	2.55 × 10^0^	1.05 × 10^1^	9.04 × 10^0^	**3.15 × 10^−1^**	2.20 × 10^0^	9.76 × 10^0^	2.05 × 10^0^	1.12 × 10^1^	1.09 × 10^1^
F14	mean	**0.00 × 10^0^**	1.67 × 10^1^	2.57 × 10^1^	**0.00 × 10^0^**	**0.00 × 10^0^**	2.54 × 10^1^	**0.00 × 10^0^**	2.40 × 10^1^	2.00 × 10^1^
	std	**0.00 × 10^0^**	1.12 × 10^1^	9.10 × 10^0^	**0.00 × 10^0^**	**0.00 × 10^0^**	9.25 × 10^0^	**0.00 × 10^0^**	8.70 × 10^0^	1.27 × 10^1^
F15	mean	6.55 × 10^−2^	1.25 × 10^1^	1.18 × 10^1^	**4.35 × 10^−2^**	1.02 × 10^−1^	9.21 × 10^0^	6.47 × 10^−2^	9.53 × 10^0^	1.09 × 10^1^
	std	1.43 × 10^−1^	6.03 × 10^0^	3.75 × 10^0^	**1.23 × 10^−1^**	1.69 × 10^−1^	3.76 × 10^0^	1.33 × 10^−1^	3.63 × 10^0^	8.11 × 10^0^
F16	mean	3.22 × 10^−1^	3.54 × 10^2^	2.76 × 10^2^	**2.50 × 10^−1^**	3.51 × 10^−1^	3.98 × 10^2^	3.42 × 10^−1^	4.24 × 10^2^	2.96 × 10^2^
	std	2.96 × 10^−1^	1.68 × 10^2^	2.05 × 10^2^	**1.79 × 10^−1^**	2.50 × 10^−1^	1.43 × 10^2^	2.33 × 10^−1^	1.39 × 10^2^	2.03 × 10^2^
F17	mean	2.39 × 10^0^	6.39 × 10^1^	8.44 × 10^1^	7.34 × 10^−1^	6.45 × 10^−1^	9.00 × 10^1^	**6.21 × 10^−1^**	6.28 × 10^1^	7.98 × 10^1^
	std	6.90 × 10^0^	1.13 × 10^1^	5.60 × 10^1^	6.64 × 10^−1^	7.78 × 10^−1^	5.43 × 10^1^	**5.44 × 10^−1^**	3.28 × 10^1^	3.22 × 10^1^
F18	mean	1.09 × 10^−1^	2.74 × 10^1^	2.72 × 10^1^	**2.66 × 10^−2^**	8.09 × 10^−2^	2.49 × 10^1^	6.61 × 10^−2^	2.79 × 10^1^	2.82 × 10^1^
	std	1.77 × 10^−1^	6.06 × 10^0^	6.78 × 10^0^	**4.94 × 10^−2^**	1.34 × 10^−1^	9.90 × 10^0^	1.35 × 10^−1^	1.33 × 10^1^	4.95 × 10^0^
F19	mean	**0.00 × 10^0^**	8.49 × 10^0^	1.07 × 10^1^	2.93 × 10^−3^	8.76 × 10^−3^	1.08 × 10^1^	1.02 × 10^−2^	9.72 × 10^0^	8.85 × 10^0^
	std	**0.00 × 10^0^**	1.04 × 10^0^	2.35 × 10^0^	6.58 × 10^−3^	1.18 × 10^−2^	3.49 × 10^0^	1.07 × 10^−2^	2.47 × 10^0^	3.36 × 10^0^
F20	mean	**0.00 × 10^0^**	8.53 × 10^1^	7.38 × 10^1^	**0.00 × 10^0^**	**0.00 × 10^0^**	5.68 × 10^1^	**0.00 × 10^0^**	9.78 × 10^1^	6.74 × 10^1^
	std	**0.00 × 10^0^**	5.70 × 10^1^	5.91 × 10^1^	**0.00 × 10^0^**	**0.00 × 10^0^**	5.01 × 10^1^	**0.00 × 10^0^**	6.55 × 10^1^	4.01 × 10^1^
F21	mean	1.76 × 10^2^	2.47 × 10^2^	2.53 × 10^2^	**1.54 × 10^2^**	1.76 × 10^2^	2.45 × 10^2^	1.82 × 10^2^	2.50 × 10^2^	2.44 × 10^2^
	std	5.25 × 10^1^	1.41 × 10^1^	1.13 × 10^1^	5.67 × 10^1^	5.13 × 10^1^	1.88 × 10^1^	4.84 × 10^1^	1.77 × 10^1^	**8.74 × 10^0^**
F22	mean	**8.18 × 10^1^**	8.19 × 10^2^	1.00 × 10^2^	8.35 × 10^1^	1.01 × 10^2^	1.00 × 10^2^	9.57 × 10^1^	4.97 × 10^2^	1.00 × 10^2^
	std	4.02 × 10^1^	1.52 × 10^3^	2.14 × 10^−13^	3.69 × 10^1^	4.60 × 10^−1^	3.74 × 10^−13^	2.25 × 10^1^	1.23 × 10^3^	**1.92 × 10^−13^**
F23	mean	3.07 × 10^2^	4.09 × 10^2^	4.10 × 10^2^	3.07 × 10^2^	**3.06 × 10^2^**	3.95 × 10^2^	3.07 × 10^2^	3.94 × 10^2^	3.96 × 10^2^
	std	**1.57 × 10^0^**	1.57 × 10^1^	1.43 × 10^1^	1.89 × 10^0^	2.56 × 10^0^	1.72 × 10^1^	2.66 × 10^0^	1.17 × 10^1^	9.24 × 10^0^
F24	mean	3.36 × 10^2^	4.85 × 10^2^	4.69 × 10^2^	3.36 × 10^2^	3.26 × 10^2^	4.77 × 10^2^	**3.03 × 10^2^**	4.71 × 10^2^	4.75 × 10^2^
	std	**4.00 × 10^0^**	1.43 × 10^1^	1.34 × 10^1^	5.25 × 10^0^	5.32 × 10^1^	2.30 × 10^1^	8.74 × 10^1^	1.49 × 10^1^	1.78 × 10^1^
F25	mean	4.07 × 10^2^	**3.87 × 10^2^**	**3.87 × 10^2^**	4.08 × 10^2^	4.00 × 10^2^	**3.87 × 10^2^**	4.03 × 10^2^	**3.87 × 10^2^**	**3.87 × 10^2^**
	std	1.97 × 10^1^	**6.07 × 10^−2^**	1.05 × 10^−1^	2.02 × 10^1^	1.02 × 10^1^	1.65 × 10^−1^	1.40 × 10^1^	1.15 × 10^−1^	1.19 × 10^−1^
F26	mean	**3.00 × 10^2^**	1.47 × 10^3^	1.39 × 10^3^	**3.00 × 10^2^**	**3.00 × 10^2^**	1.51 × 10^3^	**3.00 × 10^2^**	1.50 × 10^3^	1.47 × 10^3^
	std	**0.00 × 10^0^**	1.81 × 10^2^	1.56 × 10^2^	**0.00 × 10^0^**	**0.00 × 10^0^**	1.55 × 10^2^	**0.00 × 10^0^**	1.45 × 10^2^	1.91 × 10^2^
F27	mean	**3.89 × 10^2^**	5.05 × 10^2^	4.96 × 10^2^	**3.89 × 10^2^**	**3.89 × 10^2^**	4.95 × 10^2^	**3.89 × 10^2^**	5.02 × 10^2^	4.98 × 10^2^
	std	8.31 × 10^−1^	5.77 × 10^0^	1.29 × 10^1^	**2.16 × 10^−1^**	7.73 × 10^−1^	1.42 × 10^1^	2.58 × 10^−1^	9.35 × 10^0^	7.50 × 10^0^
F28	mean	3.28 × 10^2^	3.63 × 10^2^	3.70 × 10^2^	3.60 × 10^2^	3.57 × 10^2^	3.45 × 10^2^	3.30 × 10^2^	3.34 × 10^2^	**3.25 × 10^2^**
	std	8.97 × 10^1^	5.44 × 10^1^	7.67 × 10^1^	1.26 × 10^2^	1.16 × 10^2^	5.76 × 10^1^	9.18 × 10^1^	5.49 × 10^1^	**5.39 × 10^1^**
F29	mean	2.32 × 10^2^	4.99 × 10^2^	4.81 × 10^2^	**2.31 × 10^2^**	2.34 × 10^2^	4.96 × 10^2^	2.32 × 10^2^	5.16 × 10^2^	4.90 × 10^2^
	std	4.80 × 10^0^	4.96 × 10^1^	4.12 × 10^1^	4.70 × 10^0^	4.18 × 10^0^	3.94 × 10^1^	**4.11 × 10^0^**	7.78 × 10^1^	7.18 × 10^1^
F30	mean	**3.95 × 10^2^**	2.08 × 10^3^	2.11 × 10^3^	**3.95 × 10^2^**	3.97 × 10^2^	2.08 × 10^3^	3.96 × 10^2^	2.06 × 10^3^	2.02 × 10^3^
	std	**5.10 × 10^−2^**	1.13 × 10^2^	2.06 × 10^2^	6.86 × 10^−2^	1.08 × 10^1^	1.47 × 10^2^	3.99 × 10^0^	1.55 × 10^2^	8.53 × 10^1^

**Table 9:** Parameter analysis on *n* and *N*_max_ on the 30-dimensional CEC2017 test set.

		*n* = 13			*n* = 15			*n* = 20		
Function		*N_max_* = 9	*N_max_* = 10	*N_max_* = 11	*N_max_* = 9	*N_max_* = 10	*N_max_* = 11	*N_max_* = 9	*N_max_* = 10	*N_max_* = 11
F1	mean	**3.69 × 10^−14^**	6.75 × 10^−14^	6.39 × 10^−14^	1.78 × 10^−13^	5.68 × 10^−14^	5.61 × 10^−14^	5.68 × 10^−14^	7.11 × 10^−14^	4.83 × 10^−14^
	std	2.14 × 10^−14^	3.63 × 10^−14^	3.62 × 10^−14^	5.81 × 10^−13^	2.32 × 10^−14^	2.55 × 10^−14^	1.77 × 10^−14^	6.98 × 10^−14^	**1.62 × 10^−14^**
F3	mean	**1.48 × 10^−13^**	1.65 × 10^−13^	1.53 × 10^−13^	1.65 × 10^−13^	1.65 × 10^−13^	1.79 × 10^−13^	1.71 × 10^−13^	1.53 × 10^−13^	1.76 × 10^−13^
	std	**5.49 × 10^−14^**	9.02 × 10^−14^	6.02 × 10^−14^	1.04 × 10^−13^	5.65 × 10^−14^	6.19 × 10^−14^	5.99 × 10^−14^	6.42 × 10^−14^	6.36 × 10^−14^
F4	mean	4.80 × 10^1^	5.18 × 10^1^	**4.21 × 10^1^**	4.54 × 10^1^	4.27 × 10^1^	4.56 × 10^1^	5.42 × 10^1^	4.52 × 10^1^	5.53 × 10^1^
	std	2.54 × 10^1^	2.14 × 10^1^	2.91 × 10^1^	2.61 × 10^1^	2.95 × 10^1^	2.71 × 10^1^	**1.78 × 10^1^**	2.64 × 10^1^	1.85 × 10^1^
F5	mean	4.28 × 10^1^	4.99 × 10^1^	5.28 × 10^1^	4.82 × 10^1^	4.53 × 10^1^	4.84 × 10^1^	**3.93 × 10^1^**	4.58 × 10^1^	4.52 × 10^1^
	std	1.82 × 10^1^	1.44 × 10^1^	1.77 × 10^1^	1.99 × 10^1^	**1.15 × 10^1^**	1.41 × 10^1^	1.16 × 10^1^	1.27 × 10^1^	1.21 × 10^1^
F6	mean	2.51 × 10^−4^	6.79 × 10^−6^	5.67 × 10^−6^	1.91 × 10^−5^	2.74 × 10^−6^	8.74 × 10^−6^	**1.60 × 10^−6^**	3.99 × 10^−6^	3.80 × 10^−6^
	std	7.91 × 10^−4^	1.79 × 10^−5^	9.42 × 10^−6^	3.78 × 10^−5^	5.34 × 10^−6^	1.81 × 10^−5^	**1.42 × 10^−6^**	5.45 × 10^−6^	5.57 × 10^−6^
F7	mean	8.77 × 10^1^	8.64 × 10^1^	7.97 × 10^1^	8.89 × 10^1^	8.04 × 10^1^	8.54 × 10^1^	**7.40 × 10^1^**	8.42 × 10^1^	7.60 × 10^1^
	std	**1.10 × 10^1^**	1.65 × 10^1^	1.47 × 10^1^	1.49 × 10^1^	1.30 × 10^1^	1.73 × 10^1^	1.43 × 10^1^	1.40 × 10^1^	1.11 × 10^1^
F8	mean	4.57 × 10^1^	5.03 × 10^1^	5.20 × 10^1^	5.48 × 10^1^	**4.14 × 10^1^**	5.52 × 10^1^	4.80 × 10^1^	5.19 × 10^1^	4.54 × 10^1^
	std	1.51 × 10^1^	1.98 × 10^1^	1.09 × 10^1^	1.45 × 10^1^	1.49 × 10^1^	1.71 × 10^1^	**1.05 × 10^1^**	1.37 × 10^1^	1.59 × 10^1^
F9	mean	9.98 × 10^−2^	2.54 × 10^−1^	3.98 × 10^−1^	1.40 × 10^−1^	**5.68 × 10^−14^**	5.89 × 10^−2^	2.00 × 10^−1^	1.50 × 10^−1^	6.81 × 10^−2^
	std	2.11 × 10^−1^	4.81 × 10^−1^	8.99 × 10^−1^	2.86 × 10^−1^	**5.99 × 10^−14^**	1.54 × 10^−1^	3.23 × 10^−1^	4.90 × 10^−1^	1.66 × 10^−1^
F10	mean	3.17 × 10^3^	3.24 × 10^3^	3.37 × 10^3^	2.91 × 10^3^	3.15 × 10^3^	2.91 × 10^3^	**2.67 × 10^3^**	2.97 × 10^3^	3.00 × 10^3^
	std	6.05 × 10^2^	6.07 × 10^2^	**4.57 × 10^2^**	5.17 × 10^2^	5.66 × 10^2^	5.22 × 10^2^	8.36 × 10^2^	6.44 × 10^2^	6.55 × 10^2^
F11	mean	1.25 × 10^1^	1.14 × 10^1^	1.45 × 10^1^	1.06 × 10^1^	**9.65 × 10^0^**	1.06 × 10^1^	1.61 × 10^1^	1.30 × 10^1^	1.26 × 10^1^
	std	4.88 × 10^0^	4.18 × 10^0^	1.04 × 10^1^	**3.45 × 10^0^**	4.48 × 10^0^	4.71 × 10^0^	7.00 × 10^0^	7.28 × 10^0^	4.02 × 10^0^
F12	mean	5.13 × 10^3^	3.12 × 10^3^	3.76 × 10^3^	4.20 × 10^3^	**3.06 × 10^3^**	6.87 × 10^3^	6.93 × 10^3^	6.07 × 10^3^	4.17 × 10^3^
	std	3.78 × 10^3^	3.90 × 10^3^	**2.73 × 10^3^**	4.77 × 10^3^	4.38 × 10^3^	1.39 × 10^4^	8.04 × 10^3^	7.83 × 10^3^	6.06 × 10^3^
F13	mean	**2.18 × 10^1^**	2.97 × 10^1^	2.85 × 10^1^	2.93 × 10^1^	2.89 × 10^1^	2.60 × 10^1^	2.94 × 10^1^	2.77 × 10^1^	2.94 × 10^1^
	std	1.09 × 10^1^	9.04 × 10^0^	**8.11 × 10^0^**	1.28 × 10^1^	1.22 × 10^1^	8.86 × 10^0^	1.19 × 10^1^	1.04 × 10^1^	1.13 × 10^1^
F14	mean	2.09 × 10^1^	1.88 × 10^1^	2.01 × 10^1^	2.25 × 10^1^	2.52 × 10^1^	**1.72 × 10^1^**	2.59 × 10^1^	1.91 × 10^1^	2.30 × 10^1^
	std	1.14 × 10^1^	1.05 × 10^1^	1.20 × 10^1^	1.11 × 10^1^	9.88 × 10^0^	1.17 × 10^1^	**7.63 × 10^0^**	1.12 × 10^1^	1.14 × 10^1^
F15	mean	8.80 × 10^0^	1.12 × 10^1^	1.04 × 10^1^	9.80 × 10^0^	1.01 × 10^1^	1.12 × 10^1^	**7.63 × 10^0^**	1.00 × 10^1^	1.05 × 10^1^
	std	**1.94 × 10^0^**	4.96 × 10^0^	4.46 × 10^0^	3.93 × 10^0^	2.28 × 10^0^	3.81 × 10^0^	2.39 × 10^0^	5.07 × 10^0^	4.52 × 10^0^
F16	mean	3.33 × 10^2^	3.18 × 10^2^	3.56 × 10^2^	3.93 × 10^2^	4.29 × 10^2^	**3.00 × 10^2^**	3.43 × 10^2^	3.80 × 10^2^	4.18 × 10^2^
	std	**1.41 × 10^2^**	1.88 × 10^2^	1.81 × 10^2^	1.52 × 10^2^	1.58 × 10^2^	1.78 × 10^2^	1.90 × 10^2^	1.81 × 10^2^	1.86 × 10^2^
F17	mean	7.30 × 10^1^	7.26 × 10^1^	6.46 × 10^1^	7.05 × 10^1^	7.43 × 10^1^	**6.22 × 10^1^**	7.62 × 10^1^	7.39 × 10^1^	6.58 × 10^1^
	std	4.35 × 10^1^	2.82 × 10^1^	**1.21 × 10^1^**	3.84 × 10^1^	1.33 × 10^1^	1.81 × 10^1^	5.93 × 10^1^	6.10 × 10^1^	2.18 × 10^1^
F18	mean	2.75 × 10^1^	2.89 × 10^1^	**2.39 × 10^1^**	2.74 × 10^1^	2.92 × 10^1^	2.79 × 10^1^	2.88 × 10^1^	2.98 × 10^1^	3.08 × 10^1^
	std	5.91 × 10^0^	3.71 × 10^0^	9.40 × 10^0^	**3.55 × 10^0^**	9.91 × 10^0^	3.64 × 10^0^	**8.51 × 10^0^**	7.37 × 10^0^	5.39 × 10^0^
F19	mean	1.04 × 10^1^	1.03 × 10^1^	9.63 × 10^0^	9.80 × 10^0^	1.01 × 10^1^	9.07 × 10^0^	**8.96 × 10^0^**	9.40 × 10^0^	9.28 × 10^0^
	std	**1.85 × 10^0^**	2.32 × 10^0^	2.45 × 10^0^	2.37 × 10^0^	3.27 × 10^0^	2.23 × 10^0^	2.09 × 10^0^	2.80 × 10^0^	2.34 × 10^0^
F20	mean	6.48 × 10^1^	9.03 × 10^1^	6.86 × 10^1^	7.32 × 10^1^	8.54 × 10^1^	5.79 × 10^1^	9.76 × 10^1^	8.62 × 10^1^	**4.79 × 10^1^**
	std	6.19 × 10^1^	7.46 × 10^1^	4.66 × 10^1^	5.77 × 10^1^	6.07 × 10^1^	4.29 × 10^1^	6.96 × 10^1^	7.47 × 10^1^	**4.24 × 10^1^**
F21	mean	2.62 × 10^2^	2.52 × 10^2^	2.55 × 10^2^	2.52 × 10^2^	2.53 × 10^2^	2.49 × 10^2^	2.48 × 10^2^	2.47 × 10^2^	**2.46 × 10^2^**
	std	1.54 × 10^1^	1.74 × 10^1^	1.93 × 10^1^	**1.22 × 10^1^**	1.42 × 10^1^	1.68 × 10^1^	1.46 × 10^1^	1.47 × 10^1^	1.55 × 10^1^
F22	mean	**1.00 × 10^2^**	2.58 × 10^2^	4.87 × 10^2^	**1.00 × 10^2^**	**1.00 × 10^2^**	4.51 × 10^2^	3.93 × 10^2^	4.69 × 10^2^	**1.00 × 10^2^**
	std	**1.92 × 10^−13^**	7.04 × 10^2^	1.23 × 10^3^	7.56 × 10^−1^	2.35 × 10^−13^	1.08 × 10^3^	9.28 × 10^2^	1.14 × 10^3^	2.23 × 10^−13^
F23	mean	4.01 × 10^2^	4.01 × 10^2^	4.04 × 10^2^	3.96 × 10^2^	4.04 × 10^2^	3.97 × 10^2^	3.93 × 10^2^	**3.91 × 10^2^**	4.03 × 10^2^
	std	1.21 × 10^1^	2.00 × 10^1^	2.08 × 10^1^	1.23 × 10^1^	1.49 × 10^1^	1.63 × 10^1^	**1.01 × 10^1^**	1.34 × 10^1^	1.45 × 10^1^
F24	mean	4.74 × 10^2^	4.73 × 10^2^	**4.63 × 10^2^**	4.76 × 10^2^	4.64 × 10^2^	4.68 × 10^2^	4.67 × 10^2^	4.67 × 10^2^	4.65 × 10^2^
	std	1.44 × 10^1^	1.46 × 10^1^	**9.64 × 10^0^**	1.19 × 10^1^	1.16 × 10^1^	1.15 × 10^1^	1.68 × 10^1^	1.32 × 10^1^	1.37 × 10^1^
F25	mean	**3.87 × 10^2^**	**3.87 × 10^2^**	**3.87 × 10^2^**	**3.87 × 10^2^**	**3.87 × 10^2^**	**3.87 × 10^2^**	**3.87 × 10^2^**	**3.87 × 10^2^**	**3.87 × 10^2^**
	std	1.19 × 10^−1^	1.14 × 10^−1^	1.53 × 10^−1^	7.74 × 10^−1^	1.19 × 10^−1^	1.23 × 10^−1^	9.81 × 10^−2^	1.22 × 10^−1^	8.06 × 10^−2^
F26	mean	1.57 × 10^3^	1.45 × 10^3^	1.57 × 10^3^	1.46 × 10^3^	1.51 × 10^3^	1.48 × 10^3^	1.51 × 10^3^	**1.43 × 10^3^**	1.46 × 10^3^
	std	1.48 × 10^2^	1.82 × 10^2^	2.32 × 10^2^	1.76 × 10^2^	1.24 × 10^2^	1.69 × 10^2^	**1.06 × 10^2^**	1.64 × 10^2^	1.79 × 10^2^
F27	mean	5.03 × 10^2^	5.00 × 10^2^	4.99 × 10^2^	5.02 × 10^2^	4.99 × 10^2^	5.03 × 10^2^	5.00 × 10^2^	**4.94 × 10^2^**	5.01 × 10^2^
	std	**5.97 × 10^0^**	7.24 × 10^0^	1.02 × 10^1^	1.10 × 10^1^	9.41 × 10^0^	6.41 × 10^0^	1.55 × 10^1^	9.94 × 10^0^	1.04 × 10^1^
F28	mean	3.33 × 10^2^	3.36 × 10^2^	3.33 × 10^2^	3.25 × 10^2^	3.44 × 10^2^	3.33 × 10^2^	3.32 × 10^2^	3.44 × 10^2^	**3.27 × 10^2^**
	std	5.18 × 10^1^	5.66 × 10^1^	5.34 × 10^1^	5.16 × 10^1^	5.55 × 10^1^	**5.12 × 10^1^**	5.17 × 10^1^	5.54 × 10^1^	5.57 × 10^1^
F29	mean	5.05 × 10^2^	**4.92 × 10^2^**	5.00 × 10^2^	5.07 × 10^2^	5.11 × 10^2^	5.10 × 10^2^	4.95 × 10^2^	4.95 × 10^2^	5.21 × 10^2^
	std	5.00 × 10^1^	5.96 × 10^1^	5.60 × 10^1^	6.22 × 10^1^	8.22 × 10^1^	6.61 × 10^1^	**3.63 × 10^1^**	6.15 × 10^1^	9.69 × 10^1^
F30	mean	2.06 × 10^3^	2.02 × 10^3^	2.09 × 10^3^	2.04 × 10^3^	2.00 × 10^3^	2.05 × 10^3^	2.01 × 10^3^	2.03 × 10^3^	**1.76 × 10^−13^**
	std	9.54 × 10^1^	5.21 × 10^1^	1.14 × 10^2^	8.59 × 10^1^	5.44 × 10^1^	9.92 × 10^1^	5.37 × 10^1^	8.21 × 10^1^	**6.36 × 10^−14^**

**Table 10:** Parameter analysis on *n* and *N*_max_ on the 50-dimensional CEC2017 test set.

		*n* = 13			*n* = 15			*n* = 20		
Function		*N_max_* = 10	*N_max_* = 9	*N_max_* = 11	*N_max_* = 10	*N_max_* = 9	*N_max_* = 11	*N_max_* = 10	*N_max_* = 9	*N_max_* = 11
F1	mean	7.02 × 10^1^	1.49 × 10^2^	1.75 × 10^2^	2.87 × 10^1^	**1.07 × 10^1^**	1.74 × 10^2^	3.01 × 10^1^	1.57 × 10^1^	1.08 × 10^1^
	std	1.07 × 10^2^	2.31 × 10^2^	3.72 × 10^2^	4.33 × 10^1^	**1.16 × 10^1^**	2.85 × 10^2^	4.98 × 10^1^	2.57 × 10^1^	1.80 × 10^1^
F3	mean	2.53 × 10^−2^	9.68 × 10^−3^	1.96 × 10^−2^	1.55 × 10^−2^	1.24 × 10^−2^	3.35 × 10^−2^	3.45 × 10^−2^	5.45 × 10^−2^	**7.53 × 10** **^−3^**
	std	3.60 × 10^−2^	9.36 × 10^−3^	2.34 × 10^−2^	1.81 × 10^−2^	1.56 × 10^−2^	4.22 × 10^−2^	3.59 × 10^−2^	7.54 × 10^−2^	**6.45 × 10** **^−3^**
F4	mean	8.29 × 10^1^	7.79 × 10^1^	1.19 × 10^2^	**6.93 × 10^1^**	1.00 × 10^2^	9.55 × 10^1^	9.92 × 10^1^	7.00 × 10^1^	7.62 × 10^1^
	std	4.66 × 10^1^	5.01 × 10^1^	5.17 × 10^1^	**4.14 × 10^1^**	4.97 × 10^1^	5.29 × 10^1^	6.27 × 10^1^	5.17 × 10^1^	4.99 × 10^1^
F5	mean	1.04 × 10^2^	9.35 × 10^1^	9.86 × 10^1^	9.24 × 10^1^	9.80 × 10^1^	9.06 × 10^1^	9.90 × 10^1^	**8.78 × 10^1^**	9.71 × 10^1^
	std	2.56 × 10^1^	2.59 × 10^1^	2.93 × 10^1^	2.11 × 10^1^	2.48 × 10^1^	1.87 × 10^1^	1.88 × 10^1^	**2.23 × 10^1^**	2.22 × 10^1^
F6	mean	**3.97 × 10^−4^**	2.80 × 10^−3^	1.16 × 10^−3^	2.86 × 10^−3^	1.22 × 10^−3^	5.46 × 10^−4^	1.18 × 10^−3^	1.65 × 10^−3^	2.45 × 10^−3^
	std	**2.75 × 10^−4^**	3.47 × 10^−3^	2.26 × 10^−3^	1.04 × 10^−2^	2.46 × 10^−3^	5.47 × 10^−4^	2.00 × 10^−3^	3.64 × 10^−3^	5.35 × 10^−3^
F7	mean	**1.45 × 10^2^**	1.54 × 10^2^	1.73 × 10^2^	1.59 × 10^2^	1.60 × 10^2^	1.49 × 10^2^	1.65 × 10^2^	1.58 × 10^2^	1.47 × 10^2^
	std	2.44 × 10^1^	2.43 × 10^1^	3.30 × 10^1^	2.75 × 10^1^	2.13 × 10^1^	3.09 × 10^1^	2.18 × 10^1^	2.25 × 10^1^	**1.84 × 10^1^**
F8	mean	9.50 × 10^1^	1.12 × 10^2^	9.61 × 10^1^	9.82 × 10^1^	1.05 × 10^2^	9.30 × 10^1^	**8.75 × 10^1^**	1.03 × 10^2^	9.13 × 10^1^
	std	2.13 × 10^1^	2.81 × 10^1^	2.39 × 10^1^	3.11 × 10^1^	3.18 × 10^1^	2.89 × 10^1^	2.29 × 10^1^	**1.60 × 10^1^**	1.77 × 10^1^
F9	mean	7.98 × 10^0^	8.21 × 10^1^	7.02 × 10^0^	1.19 × 10^1^	8.52 × 10^0^	7.51 × 10^0^	**2.03 × 10^0^**	6.23 × 10^0^	8.96 × 10^0^
	std	7.65 × 10^0^	2.32 × 10^2^	8.67 × 10^0^	1.83 × 10^1^	6.06 × 10^0^	7.03 × 10^0^	**1.20 × 10^0^**	5.27 × 10^0^	1.34 × 10^1^
F10	mean	6.53 × 10^3^	5.72 × 10^3^	5.67 × 10^3^	6.25 × 10^3^	6.13 × 10^3^	5.66 × 10^3^	5.66 × 10^3^	5.66 × 10^3^	**5.33 × 10^3^**
	std	9.03 × 10^2^	7.83 × 10^2^	**5.83 × 10^2^**	1.13 × 10^3^	8.55 × 10^2^	7.18 × 10^2^	7.72 × 10^2^	9.45 × 10^2^	8.30 × 10^2^
F11	mean	5.31 × 10^1^	4.86 × 10^1^	5.71 × 10^1^	5.74 × 10^1^	6.11 × 10^1^	6.43 × 10^1^	**4.58 × 10^1^**	5.00 × 10^1^	4.92 × 10^1^
	std	2.45 × 10^1^	1.27 × 10^1^	1.45 × 10^1^	2.90 × 10^1^	3.10 × 10^1^	2.71 × 10^1^	**1.20 × 10^1^**	1.21 × 10^1^	1.57 × 10^1^
F12	mean	6.85 × 10^4^	5.93 × 10^4^	8.61 × 10^4^	6.84 × 10^4^	5.70 × 10^4^	**4.67 × 10^4^**	4.90 × 10^4^	7.54 × 10^4^	7.74 × 10^4^
	std	3.94 × 10^4^	3.19 × 10^4^	3.98 × 10^4^	4.38 × 10^4^	**1.88 × 10^4^**	1.98 × 10^4^	2.46 × 10^4^	5.08 × 10^4^	4.26 × 10^4^
F13	mean	8.84 × 10^2^	1.11 × 10^3^	1.37 × 10^3^	2.86 × 10^3^	2.44 × 10^3^	4.67 × 10^2^	1.71 × 10^3^	**1.20 × 10^3^**	7.28 × 10^2^
	std	1.45 × 10^3^	1.43 × 10^3^	3.12 × 10^3^	4.30 × 10^3^	4.67 × 10^3^	**4.02 × 10^2^**	2.69 × 10^3^	1.24 × 10^3^	8.91 × 10^2^
F14	mean	6.07 × 10^1^	**5.71 × 10^1^**	6.38 × 10^1^	6.45 × 10^1^	6.47 × 10^1^	6.06 × 10^1^	6.84 × 10^1^	5.94 × 10^1^	**5.71 × 10^1^**
	std	1.26 × 10^1^	1.22 × 10^1^	1.38 × 10^1^	9.90 × 10^0^	1.15 × 10^1^	1.35 × 10^1^	2.65 × 10^1^	**1.03 × 10^1^**	9.87 × 10^0^
F15	mean	9.59 × 10^1^	7.74 × 10^1^	8.26 × 10^1^	7.57 × 10^1^	7.74 × 10^1^	8.14 × 10^1^	**7.43 × 10^1^**	7.72 × 10^1^	9.30 × 10^1^
	std	4.02 × 10^1^	3.29 × 10^1^	3.07 × 10^1^	**2.43 × 10^1^**	2.25 × 10^1^	4.06 × 10^1^	2.83 × 10^1^	2.52 × 10^1^	3.88 × 10^1^
F16	mean	8.58 × 10^2^	1.06 × 10^3^	8.99 × 10^2^	**1.05 × 10^3^**	1.10 × 10^3^	1.07 × 10^3^	1.18 × 10^3^	9.12 × 10^2^	1.13 × 10^3^
	std	3.33 × 10^2^	3.72 × 10^2^	2.99 × 10^2^	2.65 × 10^2^	3.62 × 10^2^	2.56 × 10^2^	2.58 × 10^2^	**1.99 × 10^2^**	3.55 × 10^2^
F17	mean	6.02 × 10^2^	6.45 × 10^2^	**5.81 × 10^2^**	6.08 × 10^2^	5.88 × 10^2^	6.23 × 10^2^	6.42 × 10^2^	5.89 × 10^2^	5.98 × 10^2^
	std	1.69 × 10^2^	1.57 × 10^2^	1.84 × 10^2^	2.14 × 10^2^	1.50 × 10^2^	2.30 × 10^2^	1.52 × 10^2^	2.51 × 10^2^	**1.18 × 10^2^**
F18	mean	7.98 × 10^2^	6.48 × 10^2^	9.69 × 10^2^	**4.56 × 10^2^**	1.00 × 10^3^	1.69 × 10^3^	6.72 × 10^2^	1.15 × 10^3^	6.44 × 10^2^
	std	8.06 × 10^2^	1.09 × 10^3^	8.85 × 10^2^	**3.22 × 10^2^**	8.64 × 10^2^	1.94 × 10^3^	3.93 × 10^2^	8.54 × 10^2^	5.56 × 10^2^
F19	mean	3.52 × 10^1^	3.78 × 10^1^	4.02 × 10^1^	4.15 × 10^1^	3.66 × 10^1^	4.29 × 10^1^	3.58 × 10^1^	3.60 × 10^1^	**3.48 × 10^1^**
	std	1.06 × 10^1^	1.32 × 10^1^	1.19 × 10^1^	1.50 × 10^1^	1.60 × 10^1^	1.52 × 10^1^	9.19 × 10^0^	**8.81 × 10^0^**	1.07 × 10^1^
F20	mean	6.10 × 10^2^	5.26 × 10^2^	4.84 × 10^2^	4.17 × 10^2^	4.24 × 10^2^	5.97 × 10^2^	5.48 × 10^2^	**3.58 × 10^2^**	5.55 × 10^2^
	std	2.29 × 10^2^	1.85 × 10^2^	2.51 × 10^2^	**1.33 × 10^2^**	2.11 × 10^2^	1.70 × 10^2^	2.21 × 10^2^	1.79 × 10^2^	1.45 × 10^2^
F21	mean	2.99 × 10^2^	3.08 × 10^2^	3.03 × 10^2^	3.01 × 10^2^	3.02 × 10^2^	3.10 × 10^2^	**2.81 × 10^2^**	2.89 × 10^2^	2.99 × 10^2^
	std	2.11 × 10^1^	2.20 × 10^1^	2.97 × 10^1^	2.99 × 10^1^	2.42 × 10^1^	2.47 × 10^1^	1.68 × 10^1^	**1.64 × 10^1^**	2.80 × 10^1^
F22	mean	6.76 × 10^3^	5.94 × 10^3^	6.42 × 10^3^	6.26 × 10^3^	6.10 × 10^3^	6.35 × 10^3^	5.99 × 10^3^	**5.82 × 10^3^**	6.36 × 10^3^
	std	9.97 × 10^2^	1.06 × 10^3^	1.78 × 10^3^	2.23 × 10^3^	8.43 × 10^2^	8.17 × 10^2^	9.08 × 10^2^	**4.83 × 10^2^**	7.48 × 10^2^
F23	mean	5.30 × 10^2^	5.19 × 10^2^	5.28 × 10^2^	**5.17 × 10^2^**	5.26 × 10^2^	5.29 × 10^2^	5.23 × 10^2^	5.23 × 10^2^	5.23 × 10^2^
	std	2.53 × 10^1^	2.12 × 10^1^	2.59 × 10^1^	2.45 × 10^1^	2.98 × 10^1^	2.38 × 10^1^	**1.71 × 10^1^**	2.02 × 10^1^	2.00 × 10^1^
F24	mean	5.99 × 10^2^	5.95 × 10^2^	5.97 × 10^2^	**5.76 × 10^2^**	5.94 × 10^2^	5.95 × 10^2^	5.96 × 10^2^	5.78 × 10^2^	5.85 × 10^2^
	std	3.10 × 10^1^	2.65 × 10^1^	2.93 × 10^1^	**1.44 × 10^1^**	1.97 × 10^1^	4.22 × 10^1^	2.41 × 10^1^	1.56 × 10^1^	2.95 × 10^1^
F25	mean	5.24 × 10^2^	**5.09 × 10^2^**	5.24 × 10^2^	5.17 × 10^2^	5.27 × 10^2^	5.25 × 10^2^	5.40 × 10^2^	5.17 × 10^2^	5.26 × 10^2^
	std	3.33 × 10^1^	4.00 × 10^1^	3.28 × 10^1^	**3.08 × 10^1^**	3.77 × 10^1^	4.12 × 10^1^	3.26 × 10^1^	3.73 × 10^1^	3.39 × 10^1^
F26	mean	2.29 × 10^3^	2.24 × 10^3^	2.18 × 10^3^	2.15 × 10^3^	2.19 × 10^3^	2.22 × 10^3^	2.04 × 10^3^	**1.92 × 10^3^**	2.21 × 10^3^
	std	2.82 × 10^2^	2.43 × 10^2^	2.18 × 10^2^	2.15 × 10^2^	3.39 × 10^2^	2.71 × 10^2^	2.73 × 10^2^	**1.73 × 10^2^**	2.78 × 10^2^
F27	mean	5.42 × 10^2^	5.63 × 10^2^	5.41 × 10^2^	5.42 × 10^2^	5.47 × 10^2^	5.42 × 10^2^	**5.34 × 10^2^**	5.44 × 10^2^	5.53 × 10^2^
	std	2.53 × 10^1^	4.33 × 10^1^	2.29 × 10^1^	**1.59 × 10^1^**	1.60 × 10^1^	2.08 × 10^1^	1.67 × 10^1^	2.29 × 10^1^	1.75 × 10^1^
F28	mean	4.84 × 10^2^	4.81 × 10^2^	4.85 × 10^2^	**4.80 × 10^2^**	4.94 × 10^2^	4.78 × 10^2^	**4.80 × 10^2^**	4.82 × 10^2^	4.82 × 10^2^
	std	2.37 × 10^1^	3.00 × 10^1^	2.25 × 10^1^	2.38 × 10^1^	3.60 × 10^1^	2.52 × 10^1^	**2.30 × 10^1^**	2.45 × 10^1^	2.50 × 10^1^
F29	mean	6.94 × 10^2^	6.84 × 10^2^	6.31 × 10^2^	6.31 × 10^2^	6.41 × 10^2^	**6.18 × 10^2^**	6.31 × 10^2^	7.01 × 10^2^	5.61 × 10^2^
	std	1.76 × 10^2^	1.53 × 10^2^	2.11 × 10^2^	**1.59 × 10^2^**	1.96 × 10^2^	2.09 × 10^2^	1.74 × 10^2^	2.22 × 10^2^	1.74 × 10^2^
F30	mean	6.51 × 10^5^	6.52 × 10^5^	**6.10 × 10^5^**	6.37 × 10^5^	6.21 × 10^5^	6.22 × 10^5^	6.26 × 10^5^	6.12 × 10^5^	6.37 × 10^5^
	std	4.74 × 10^4^	9.70 × 10^4^	3.20 × 10^4^	5.73 × 10^4^	**3.04 × 10^4^**	3.14 × 10^4^	4.33 × 10^4^	4.59 × 10^4^	5.59 × 10^4^

**Table 11:** Running results of each improvement strategy in CEC2019 test.

Function		IGKSO	IGKSO11	IGKSO2	IGKSO3	GKSO
F1	Mean Std	**0.00 × 10^0^** **0.00 × 10^0^**	1.01 × 10^−10^ 4.52 × 10^−10^	9.97 × 10^−2^ 2.55 × 10^−1^	**0.00 × 10^0^** **0.00 × 10^0^**	7.33 × 10^−2^ 3.28 × 10^−1^
F2	Mean Std	**4.03 × 10^−1^** 4.93 × 10^−1^	3.36 × 10^0^ **2.25 × 10^−1^**	2.11 × 10^2^ 1.00 × 10^2^	7.35 × 10^0^ 3.60 × 10^0^	4.19 × 10^0^ 3.22 × 10^0^
F3	Mean Std	**3.32 × 10^−1^** **1.72 × 10^−1^**	6.74 × 10^−1^ 1.15 × 10^0^	4.88 × 10^−1^ 4.60 × 10^−1^	1.68 × 10^0^ 1.73 × 10^0^	1.10 × 10^0^ 1.31 × 10^0^
F4	Mean Std	**4.83 × 10^0^** 2.58 × 10^0^	1.41 × 10^1^ 5.91 × 10^0^	7.06 × 10^0^ 1.88 × 10^0^	5.52 × 10^0^ **1.38 × 10^0^**	1.02 × 10^1^ 3.18 × 10^0^
F5	Mean Std	**5.09 × 10^−2^** **3.46 × 10^−2^**	1.89 × 10^−1^ 1.01 × 10^−1^	7.06 × 10^−2^ 3.77 × 10^−2^	8.75 × 10^−2^ 4.34 × 10^−2^	8.83 × 10^−2^ 4.15 × 10^−2^
F6	Mean Std	**0.00 × 10^0^** **0.00 × 10^0^**	1.66 × 10^0^ 1.00 × 10^0^	3.41 × 10^−1^ 7.26 × 10^−1^	8.58 × 10^−1^ 1.08 × 10^0^	4.72 × 10^0^ 1.83 × 10^0^
F7	Mean Std	**6.79 × 10^1^** **1.14 × 10^2^**	5.29 × 10^2^ 2.14 × 10^2^	3.05 × 10^2^ 1.61 × 10^2^	3.23 × 10^2^ 1.86 × 10^2^	6.24 × 10^2^ 1.64 × 10^2^
F8	Mean Std	**1.71 × 10^0^** 5.37 × 10^−1^	2.46 × 10^0^ 3.89 × 10^−1^	1.95 × 10^0^ **3.10 × 10^−1^**	1.98 × 10^0^ 4.51 × 10^−1^	2.57 × 10^0^ 3.77 × 10^−1^
F9	Mean Std	**1.09 × 10^−1^** 4.07 × 10^−2^	2.07 × 10^−1^ 7.86 × 10^−2^	1.39 × 10^−1^ 3.29 × 10^−2^	1.40 × 10^−1^ 4.44 × 10^−2^	1.30 × 10^−1^ **3.24 × 10^−2^**
F10	Mean Std	**1.54 × 10^1^** 8.51 × 10^0^	1.95 × 10^1^ 3.86 × 10^0^	1.91 × 10^1^ 4.50 × 10^0^	1.70 × 10^1^ 7.33 × 10^0^	2.02 × 10^1^ **3.72 × 10^−2^**

**Table 12:** Wilcoxon rank-sum test results of each improved algorithm, GKSO algorithm and IGKSO algorithm.

Function	IGKSO11	IGKSO2	IGKSO3	IGKSO
F1	0.342 (=)	0.000 (−)	0.342 (=)	0.342 (=)
F2	0.454 (=)	0.000 (+)	0.000 (+)	0.000 (−)
F3	0.152 (=)	0.047 (−)	0.147 (=)	0.010 (−)
F4	0.070 (=)	0.000 (−)	0.000 (−)	0.000 (−)
F5	0.000 (+)	0.221 (=)	0.684 (=)	0.004 (−)
F6	0.000 (−)	0.000 (−)	0.000 (−)	0.000 (−)
F7	0.140 (=)	0.000 (−)	0.000 (−)	0.000 (−)
F8	0.394 (=)	0.000 (−)	0.000 (−)	0.000 (−)
F9	0.000 (+)	0.362 (=)	0.221 (=)	0.108 (=)
F10	0.000 (−)	0.000 (−)	0.000 (−)	0.000 (−)
(+/−/=)	2/6/2	1/7/2	1/5/4	0/8/2

**Table 13:** Friedman test results of each improved algorithm.

	IGKSO	IGKSO11	IGKSO2	IGKSO3	GKSO
Avg.rank	1	3.9	2.9	3.1	4.1
sort	1	4	2	3	5

**Table 14:** Experimental results of subspace and full-space strategies on the CEC2019 test suite.

Function		IGKSO	IGKSO4
F1	Mean Std	**0.00 × 10^0^** **0.00 × 10^0^**	**0.00 × 10^0^ (*NaN*) =** **0.00 × 10^0^**
F2	Mean Std	**4.03 × 10^−1^** 4.93 × 10^−1^	5.09 × 10^1^ (6.77 × 10^−8^) − 3.38 × 10^1^
F3	Mean Std	**3.32 × 10^−1^** **1.72 × 10^−1^**	7.09 × 10^−1^ (1.51 × 10^−1^) = 9.96 × 10^−1^
F4	Mean Std	**4.83 × 10^0^** 2.58 × 10^0^	1.09 × 10^1^ (1.78 × 10^−5^) − 4.75 × 10^0^
F5	Mean Std	**5.09 × 10^−2^** **3.46 × 10^−2^**	8.25 × 10^−2^ (7.40 × 10^−3^) − 3.58 × 10^−2^
F6	Mean Std	**0.00 × 10^0^** **0.00 × 10^0^**	6.01 × 10^−2^ (1.64 × 10^−4^) − 1.33 × 10^−1^
F7	Mean Std	**6.79 × 10^1^** **1.14 × 10^2^**	4.97 × 10^2^ (5.25 × 10^−5^) − 2.98 × 10^2^
F8	Mean Std	**1.71 × 10^0^** 5.37 × 10^−1^	2.08 × 10^0^ (5.83 × 10^−2^) = **5.01 × 10^−1^**
F9	Mean Std	**1.09 × 10^−1^** 4.07 × 10^−2^	1.19 × 10^−1^ (6.06 × 10^−1^) = 4.31 × 10^−2^
F10	Mean Std	**1.54 × 10^1^** 8.51 × 10^0^	1.77 × 10^1^ (3.07 × 10^−1^) = **6.16 × 10^0^**
(+/−/=)		**Base**	**0/5/5**

**Table 15:** The parameter configurations of all comparative algorithms.

Algorithm	Related Parameters
GKSO	*m* = 1.5
ALA	*R* = [−1, 1]; *F* = 1/−1; *β* = 1.5;
MDBO	yz = 1 × 10^−5^; RDBN_np = 0.2 × np; YDBN_np = 0.2 × np, DDBN_np = round (0.25 × np); TDBN_np = np-YDBN_np-RDBN_np-DDBN_np
MTV-SCA	λ = 0.25; *T* = 3000; *D* = 5; *limit* = [−100, 100]
TTHHO	*T* = 3000
IGKSO1	*T* = 967; *m* = 1.5
MGKSO	*T* = 1500; *m* = 1.5
EGKSO	*T* = 967 *F*0 = 0.5 *m* = 1.5
IAOA	*C*2 = 6; *C*3 = 2; *C*4 = 0.5

**Table 16:** Data results on the 30-dimensional CEC2017 test set.

Function		IGKSO	GKSO	IGKSO1	EGKSO	MGKSO	IAOA	MTV-SCA	MDBO	TTHHO	ALA
F1	mean	**5.68 × 10^−14^**	6.86 × 10^3^	7.97 × 10^3^	5.58 × 10^3^	1.04 × 10^4^	1.40 × 10^0^	2.32 × 10^−7^	1.12 × 10^3^	3.35 × 10^10^	1.18 × 10^3^
	std	**2.57 × 10^−14^**	6.74 × 10^3^	4.79 × 10^3^	4.62 × 10^3^	8.33 × 10^3^	4.78 × 10^0^	5.46 × 10^−7^	1.25 × 10^3^	1.03 × 10^10^	1.64 × 10^3^
F3	mean	**1.62 × 10^−13^**	2.12 × 10^1^	3.63 × 10^−4^	6.76 × 10^−7^	3.32 × 10^2^	2.74 × 10^−1^	8.01 × 10^−1^	3.85 × 10^−10^	8.55 × 10^4^	6.02 × 10^−4^
	std	**4.97 × 10^−14^**	5.07 × 10^1^	2.61 × 10^−4^	5.99 × 10^−7^	3.76 × 10^2^	8.29 × 10^−1^	1.93 × 10^0^	8.45 × 10^−10^	9.13 × 10^3^	1.51 × 10^−3^
F4	mean	4.85 × 10^1^	6.20 × 10^1^	4.64 × 10^1^	2.42 × 10^1^	8.97 × 10^1^	3.43 × 10^1^	5.47 × 10^1^	1.05 × 10^1^	5.68 × 10^3^	**9.89 × 10^0^**
	std	2.41 × 10^1^	2.78 × 10^1^	3.38 × 10^1^	3.04 × 10^1^	2.64 × 10^1^	3.01 × 10^1^	3.72 × 10^1^	2.24 × 10^1^	1.73 × 10^3^	**1.54 × 10^1^**
F5	mean	5.07 × 10^1^	9.76 × 10^1^	9.85 × 10^1^	8.25 × 10^1^	9.19 × 10^1^	2.89 × 10^1^	4.59 × 10^1^	**3.16 × 10^1^**	3.59 × 10^2^	6.98 × 10^1^
	std	9.79 × 10^0^	2.17 × 10^1^	2.36 × 10^1^	1.37 × 10^1^	2.31 × 10^1^	**8.34 × 10^0^**	1.03 × 10^1^	1.39 × 10^1^	2.96 × 10^1^	1.25 × 10^1^
F6	mean	**4.78 × 10^−6^**	1.20 × 10^1^	9.37 × 10^−1^	1.18 × 10^1^	1.55 × 10^1^	1.92 × 10^0^	2.86 × 10^−1^	7.71 × 10^−3^	6.19 × 10^1^	1.19 × 10^−1^
	std	**1.33 × 10^−5^**	4.36 × 10^0^	5.64 × 10^−1^	3.97 × 10^0^	6.39 × 10^0^	1.66 × 10^0^	3.03 × 10^−1^	1.82 × 10^−2^	3.83 × 10^0^	2.83 × 10^−1^
F7	mean	7.97 × 10^1^	1.48 × 10^2^	1.35 × 10^2^	1.33 × 10^2^	1.67 × 10^2^	6.93 × 10^1^	1.10 × 10^2^	**6.38 × 10^1^**	7.55 × 10^2^	1.13 × 10^2^
	std	1.56 × 10^1^	2.27 × 10^1^	2.34 × 10^1^	1.84 × 10^1^	3.63 × 10^1^	1.73 × 10^1^	2.01 × 10^1^	**9.11 × 10^0^**	5.61 × 10^1^	1.60 × 10^1^
F8	mean	5.35 × 10^1^	9.76 × 10^1^	8.79 × 10^1^	8.32 × 10^1^	7.80 × 10^1^	**2.80 × 10^1^**	5.34 × 10^1^	3.29 × 10^1^	3.75 × 10^2^	6.65 × 10^1^
	std	1.73 × 10^1^	2.03 × 10^1^	2.08 × 10^1^	1.96 × 10^1^	2.34 × 10^1^	**6.09 × 10^0^**	1.43 × 10^1^	1.03 × 10^1^	4.76 × 10^1^	1.26 × 10^1^
F9	mean	**2.36 × 10^−1^**	6.05 × 10^2^	4.61 × 10^2^	4.73 × 10^2^	5.93 × 10^2^	1.64 × 10^1^	6.44 × 10^1^	5.28 × 10^0^	7.42 × 10^3^	2.23 × 10^2^
	std	**5.18 × 10^−1^**	2.91 × 10^2^	2.30 × 10^2^	2.84 × 10^2^	3.77 × 10^2^	2.06 × 10^1^	7.20 × 10^1^	4.71 × 10^0^	9.71 × 10^2^	2.86 × 10^2^
F10	mean	2.98 × 10^3^	3.03 × 10^3^	**2.31 × 10^3^**	2.79 × 10^3^	3.98 × 10^3^	3.77 × 10^3^	3.23 × 10^3^	3.41 × 10^3^	6.01 × 10^3^	3.56 × 10^3^
	std	5.89 × 10^2^	**2.90 × 10^2^**	5.28 × 10^2^	3.27 × 10^2^	1.43 × 10^3^	5.89 × 10^2^	7.96 × 10^2^	4.11 × 10^2^	8.21 × 10^2^	6.87 × 10^2^
F11	mean	**1.21 × 10^1^**	3.67 × 10^2^	1.09 × 10^2^	1.26 × 10^2^	2.68 × 10^2^	3.07 × 10^2^	9.69 × 10^1^	3.94 × 10^1^	6.53 × 10^3^	3.46 × 10^1^
	std	**3.83 × 10^0^**	1.85 × 10^2^	3.22 × 10^1^	5.40 × 10^1^	1.23 × 10^2^	5.25 × 10^2^	5.13 × 10^1^	2.83 × 10^1^	2.24 × 10^3^	8.25 × 10^0^
F12	mean	3.10 × 10^3^	1.28× 10^5^	8.40 × 10^4^	2.06 × 10^4^	1.49× 10^5^	2.26 × 10^4^	1.33 × 10^4^	1.47 × 10^4^	5.13 × 10^9^	**1.11 × 10^3^**
	std	3.54 × 10^3^	1.26× 10^5^	6.54 × 10^4^	1.22 × 10^4^	2.91× 10^5^	1.74 × 10^4^	8.44 × 10^3^	1.08 × 10^4^	2.05 × 10^9^	**5.38 × 10^2^**
F13	mean	**2.94 × 10^1^**	1.98 × 10^4^	1.96 × 10^4^	4.01 × 10^3^	1.84 × 10^4^	2.46 × 10^4^	2.38 × 10^3^	5.30 × 10^3^	1.13 × 10^9^	1.90 × 10^2^
	std	**9.59 × 10^0^**	1.72 × 10^4^	2.02 × 10^4^	4.62 × 10^3^	2.18 × 10^4^	2.53 × 10^4^	3.69 × 10^3^	4.16 × 10^3^	1.72 × 10^9^	1.08 × 10^2^
F14	mean	**2.46 × 10^1^**	1.28 × 10^3^	2.49 × 10^2^	9.05 × 10^1^	3.69 × 10^3^	1.59 × 10^4^	1.27 × 10^2^	5.38 × 10^1^	2.39 × 10^6^	5.22 × 10^1^
	std	**8.48 × 10^0^**	2.03 × 10^3^	1.17 × 10^2^	3.49 × 10^1^	3.61 × 10^3^	6.06 × 10^4^	6.95 × 10^1^	1.73 × 10^1^	1.57 × 10^6^	1.16 × 10^1^
F15	mean	**9.04 × 10^0^**	1.11 × 10^4^	8.78 × 10^3^	2.19 × 10^2^	1.11 × 10^4^	5.46 × 10^3^	2.90 × 10^2^	2.08 × 10^2^	1.86 × 10^8^	5.79 × 10^1^
	std	**3.94 × 10^0^**	8.97 × 10^3^	9.97 × 10^3^	1.30 × 10^2^	1.23 × 10^4^	8.20 × 10^3^	6.70 × 10^2^	1.16 × 10^2^	2.90 × 10^8^	3.44 × 10^1^
F16	mean	4.09 × 10^2^	7.48 × 10^2^	6.36 × 10^2^	6.30 × 10^2^	7.36 × 10^2^	**3.40 × 10^2^**	4.56 × 10^2^	4.30 × 10^2^	2.68 × 10^3^	5.18 × 10^2^
	std	2.43 × 10^2^	2.20 × 10^2^	2.05 × 10^2^	1.82 × 10^2^	2.50 × 10^2^	**1.56 × 10^2^**	1.82 × 10^2^	2.44 × 10^2^	6.13 × 10^2^	1.98 × 10^2^
F17	mean	6.25 × 10^1^	2.16 × 10^2^	2.06 × 10^2^	1.77 × 10^2^	3.29 × 10^2^	1.00 × 10^2^	7.85 × 10^1^	**5.46 × 10^1^**	1.17 × 10^3^	1.89 × 10^2^
	std	**2.47 × 10^1^**	8.26 × 10^1^	8.01 × 10^1^	7.81 × 10^1^	1.65 × 10^2^	3.28 × 10^1^	4.99 × 10^1^	2.63 × 10^1^	3.01 × 10^2^	9.21 × 10^1^
F18	mean	**2.90 × 10^1^**	9.59 × 10^4^	1.50 × 10^4^	1.81 × 10^3^	1.70× 10^5^	4.02 × 10^4^	1.39 × 10^4^	7.48 × 10^3^	1.63 × 10^7^	6.73 × 10^1^
	std	**5.18 × 10^0^**	4.68 × 10^4^	1.50 × 10^4^	2.53 × 10^3^	1.57× 10^5^	6.36 × 10^4^	1.22 × 10^4^	1.04 × 10^4^	2.43 × 10^7^	2.42 × 10^1^
F19	mean	**8.32 × 10^0^**	8.54 × 10^3^	4.20 × 10^3^	1.55 × 10^2^	1.20 × 10^4^	9.20 × 10^3^	7.98 × 10^1^	4.37 × 10^2^	1.63 × 10^8^	3.29 × 10^1^
	std	**2.77 × 10^0^**	1.17 × 10^4^	4.36 × 10^3^	9.87 × 10^1^	1.70 × 10^4^	1.08 × 10^4^	4.21 × 10^1^	1.20 × 10^3^	2.16 × 10^8^	1.04 × 10^1^
F20	mean	**6.92 × 10^1^**	2.20 × 10^2^	2.15 × 10^2^	2.54 × 10^2^	3.93 × 10^2^	5.56 × 10^2^	1.26 × 10^2^	1.04 × 10^2^	7.64 × 10^2^	1.92 × 10^2^
	std	**5.95 × 10^1^**	8.69 × 10^1^	6.78 × 10^1^	8.40 × 10^1^	1.37 × 10^2^	2.49 × 10^2^	7.61 × 10^1^	9.63 × 10^1^	1.40 × 10^2^	9.59 × 10^1^
F21	mean	2.46 × 10^2^	2.91 × 10^2^	2.73 × 10^2^	2.71 × 10^2^	2.72 × 10^2^	2.35 × 10^2^	2.41 × 10^2^	**2.29 × 10^2^**	5.32 × 10^2^	2.59 × 10^2^
	std	9.81 × 10^0^	2.08 × 10^1^	1.76 × 10^1^	1.39 × 10^1^	1.67 × 10^1^	1.38 × 10^1^	**7.17 × 10^0^**	7.69 × 10^0^	5.44 × 10^1^	1.44 × 10^1^
F22	mean	**1.00 × 10^2^**	7.26 × 10^2^	3.79 × 10^2^	1.01 × 10^2^	1.02 × 10^2^	3.57 × 10^3^	2.56 × 10^2^	**1.00 × 10^2^**	5.79 × 10^3^	2.40 × 10^3^
	std	**2.14 × 10^−13^**	1.29 × 10^3^	8.64 × 10^2^	1.38 × 10^0^	2.25 × 10^0^	1.02 × 10^3^	6.92 × 10^2^	7.62 × 10^−1^	1.80 × 10^3^	1.77 × 10^3^
F23	mean	4.01 × 10^2^	4.52 × 10^2^	4.44 × 10^2^	4.36 × 10^2^	4.42 × 10^2^	**3.81 × 10^2^**	4.04 × 10^2^	3.84 × 10^2^	9.62 × 10^2^	4.21 × 10^2^
	std	1.51 × 10^1^	2.33 × 10^1^	2.14 × 10^1^	2.90 × 10^1^	2.54 × 10^1^	**1.07 × 10^1^**	1.70 × 10^1^	2.00 × 10^1^	1.42 × 10^2^	1.66 × 10^1^
F24	mean	4.71 × 10^2^	5.06 × 10^2^	5.16 × 10^2^	5.01 × 10^2^	5.03 × 10^2^	4.73 × 10^2^	4.69 × 10^2^	**4.49 × 10^2^**	1.08 × 10^3^	5.00 × 10^2^
	std	1.46 × 10^1^	2.01 × 10^1^	2.24 × 10^1^	2.14 × 10^1^	2.29 × 10^1^	1.92 × 10^1^	1.47 × 10^1^	**1.22 × 10^1^**	1.40 × 10^2^	1.83 × 10^1^
F25	mean	**3.87 × 10^2^**	3.95 × 10^2^	3.97 × 10^2^	3.92 × 10^2^	4.10 × 10^2^	3.98 × 10^2^	3.94 × 10^2^	3.88 × 10^2^	1.82 × 10^3^	**3.87 × 10^2^**
	std	**7.93 × 10^−1^**	1.69 × 10^1^	1.70 × 10^1^	1.45 × 10^1^	2.31 × 10^1^	1.80 × 10^1^	1.08 × 10^1^	6.11 × 10^0^	4.94 × 10^2^	8.16 × 10^−1^
F26	mean	1.56 × 10^3^	1.89 × 10^3^	1.76 × 10^3^	1.66 × 10^3^	1.57 × 10^3^	1.31 × 10^3^	1.63 × 10^3^	**1.06 × 10^3^**	6.55 × 10^3^	1.79 × 10^3^
	std	**1.47 × 10^2^**	6.42 × 10^2^	8.10 × 10^2^	8.76 × 10^2^	6.81 × 10^2^	2.10 × 10^2^	4.22 × 10^2^	5.44 × 10^2^	8.60 × 10^2^	4.09 × 10^2^
F27	mean	5.04 × 10^2^	5.40 × 10^2^	5.44 × 10^2^	5.35 × 10^2^	5.41 × 10^2^	5.85 × 10^2^	5.26 × 10^2^	5.18 × 10^2^	**5.00 × 10^2^**	5.16 × 10^2^
	std	9.50 × 10^0^	1.92 × 10^1^	1.88 × 10^1^	1.61 × 10^1^	1.54 × 10^1^	5.53 × 10^1^	1.48 × 10^1^	1.56 × 10^1^	**6.25 × 10^−5^**	2.07 × 10^1^
F28	mean	3.35 × 10^2^	3.72 × 10^2^	3.23 × 10^2^	3.22 × 10^2^	4.19 × 10^2^	5.48 × 10^2^	3.52 × 10^2^	**3.17 × 10^2^**	5.00 × 10^2^	3.65 × 10^2^
	std	5.59 × 10^1^	5.75 × 10^1^	4.09 × 10^1^	4.57 × 10^1^	2.15 × 10^1^	7.82 × 10^2^	5.79 × 10^1^	4.05 × 10^1^	**6.07 × 10^−3^**	6.65 × 10^0^
F29	mean	5.02 × 10^2^	8.58 × 10^2^	6.71 × 10^2^	7.80 × 10^2^	8.76 × 10^2^	6.51 × 10^2^	5.80 × 10^2^	**4.79 × 10^2^**	2.51 × 10^3^	6.45 × 10^0^
	std	**3.83 × 10^1^**	1.76 × 10^2^	1.15 × 10^2^	1.21 × 10^2^	1.94 × 10^2^	1.90 × 10^2^	7.19 × 10^1^	3.93 × 10^1^	8.91 × 10^2^	1.37 × 10^2^
F30	mean	**2.08 × 10^3^**	1.39 × 10^4^	6.52 × 10^3^	5.37 × 10^3^	2.22 × 10^4^	5.81 × 10^3^	4.68 × 10^3^	4.00 × 10^3^	1.51 × 10^8^	2.48 × 10^3^
	std	**1.52 × 10^2^**	7.63 × 10^3^	2.98 × 10^3^	3.40 × 10^3^	3.06 × 10^4^	3.41 × 10^3^	1.94 × 10^3^	1.32 × 10^3^	1.28 × 10^8^	6.31 × 10^2^

**Table 17:** Data results on the CEC2019 test set.

Function		IGKSO	GKSO	IGKSO1	EGKSO	MGKSO	IAOA	MTV-SCA	MDBO	TTHHO	ALA
F1	mean	**0.00 × 10^0^**	1.25 × 10^−7^	**0.00 × 10^0^**	**0.00 × 10^0^**	**0.00 × 10^0^**	1.85 × 10^1^	6.05 × 10^2^	**0.00 × 10^0^**	**0.00 × 10^0^**	1.48 × 10^9^
	std	**0.00 × 10^0^**	5.59 × 10^−7^	**0.00 × 10^0^**	**0.00 × 10^0^**	5.09 × 10^−17^	5.07 × 10^1^	1.16 × 10^3^	**0.00 × 10^0^**	**0.00 × 10^0^**	7.55 × 10^8^
F2	mean	**4.03 × 10^−1^**	3.47 × 10^0^	3.34 × 10^0^	2.08 × 10^0^	3.34× 10^0^	2.00 × 10^2^	1.44 × 10^2^	2.28 × 10^0^	3.99 × 10^0^	2.93 × 10^4^
	std	4.93 × 10^−1^	3.14 × 10^−1^	2.36 × 10^−1^	1.27 × 10^−1^	1.69 × 10^−1^	9.62 × 10^1^	7.07 × 10^1^	2.81 × 10^−1^	**5.61 × 10^−2^**	9.29 × 10^3^
F3	mean	**3.32 × 10^−1^**	9.80 × 10^−1^	4.09 × 10^−1^	4.16 × 10^−1^	1.11 × 10^0^	1.68 × 10^0^	1.10 × 10^0^	3.68 × 10^−1^	4.50 × 10^0^	1.14 × 10^1^
	std	1.72 × 10^−1^	1.46 × 10^0^	2.25 × 10^−6^	2.46 × 10^−1^	1.34 × 10^0^	1.01 × 10^0^	8.68 × 10^−1^	**1.26 × 10^−1^**	1.28 × 10^0^	4.52 × 10^−1^
F4	mean	4.83 × 10^0^	8.87 × 10^0^	1.13 × 10^1^	5.64 × 10^0^	1.55 × 10^1^	**1.53 × 10^0^**	6.34 × 10^0^	6.52 × 10^0^	6.40 × 10^1^	1.50 × 10^2^
	std	2.58 × 10^0^	2.60 × 10^0^	4.48 × 10^0^	1.89 × 10^0^	8.04 × 10^0^	**1.05 × 10^0^**	2.18 × 10^0^	2.93 × 10^0^	1.68 × 10^1^	2.28 × 10^1^
F5	mean	5.09 × 10^−2^	9.16 × 10^−2^	1.28 × 10^−1^	4.44 × 10^−2^	2.14 × 10^−1^	**1.06 × 10^−2^**	2.49 × 10^−2^	4.01 × 10^−2^	3.93 × 10^1^	1.79 × 10^2^
	std	3.46 × 10^−2^	3.88 × 10^−2^	7.26 × 10^−2^	3.64 × 10^−2^	1.33 × 10^−1^	**1.16 × 10^−2^**	2.38 × 10^−2^	1.98 × 10^−2^	1.83 × 10^1^	5.62 × 10^1^
F6	mean	**0.00 × 10^0^**	1.36 × 10^0^	1.12 × 10^0^	3.95 × 10^−2^	1.98 × 10^0^	1.44 × 10^0^	9.33 × 10^−2^	2.59 × 10^−2^	8.33 × 10^0^	1.43 × 10^1^
	std	**0.00 × 10^0^**	1.05 × 10^0^	9.58 × 10^−1^	1.10 × 10^−1^	9.41 × 10^−1^	1.29 × 10^0^	1.31 × 10^−1^	1.16 × 10^−1^	9.65 × 10^−1^	1.02 × 10^0^
F7	mean	**6.79 × 10^1^**	4.39 × 10^2^	4.28 × 10^2^	4.37 × 10^2^	6.22 × 10^2^	4.91 × 10^2^	5.07 × 10^2^	4.12 × 10^2^	1.44 × 10^3^	2.73 × 10^3^
	std	**1.14 × 10^2^**	1.80 × 10^2^	1.79 × 10^2^	2.23 × 10^2^	3.59 × 10^2^	2.92 × 10^2^	1.35 × 10^2^	2.50 × 10^2^	2.37 × 10^2^	2.92 × 10^2^
F8	mean	**1.71 × 10^0^**	1.99 × 10^0^	2.20 × 10^0^	1.98 × 10^0^	2.49 × 10^0^	2.19 × 10^0^	2.36 × 10^0^	1.93 × 10^0^	3.67 × 10^0^	4.48 × 10^0^
	std	5.37 × 10^−1^	4.82 × 10^−1^	4.78 × 10^−1^	4.40 × 10^−1^	3.32 × 10^−1^	6.05 × 10^−1^	2.47 × 10^−1^	4.28 × 10^−1^	2.49 × 10^−1^	**1.94 × 10^−1^**
F9	mean	1.09 × 10^−1^	1.41 × 10^−1^	7.13 × 10^−2^	1.19 × 10^−1^	1.91 × 10^−1^	1.04 × 10^−1^	1.68 × 10^−1^	**6.29 × 10^−2^**	1.11 × 10^0^	4.89 × 10^0^
	std	4.07 × 10^−2^	4.80 × 10^−2^	3.79 × 10^−2^	**2.10 × 10^−2^**	7.37 × 10^−2^	3.61 × 10^−2^	3.51 × 10^−2^	2.58 × 10^−2^	7.89 × 10^−1^	8.27 × 10^−1^
F10	mean	**1.54 × 10^1^**	1.74 × 10^1^	1.66 × 10^1^	1.83 × 10^1^	1.95 × 10^1^	2.02 × 10^1^	2.02 × 10^1^	1.65 × 10^1^	2.03 × 10^1^	2.11 × 10^1^
	std	8.51 × 10^0^	6.64 × 10^0^	7.34 × 10^0^	5.98 × 10^0^	3.87 × 10^0^	4.65 × 10^−2^	**5.68 × 10^−2^**	7.16 × 10^0^	1.64 × 10^−1^	1.49 × 10^−1^

**Table 18:** Wilcoxon rank-sum test results for IGKSO and other algorithms on the CEC2017 test set.

	GKSO	IGKSO1	EGKSO	MGKSO	IAOA	MTV-SCA	MDBO	TTHHO	ALA
F1	0.000 (−)	0.000 (−)	0.000 (−)	0.000 (−)	0.000 (−)	0.000 (−)	0.000 (−)	0.000 (−)	0.000 (−)
F3	0.000 (−)	0.000 (+)	0.000 (−)	0.000 (−)	0.000 (−)	0.000 (−)	0.000 (−)	0.000 (−)	0.000 (−)
F4	0.000 (−)	0.142 (=)	0.193 (=)	0.000 (−)	0.128 (=)	0.013 (−)	0.004 (+)	0.000 (−)	0.003 (+)
F5	0.000 (−)	0.000 (−)	0.000 (−)	0.000 (−)	0.000 (+)	0.099 (=)	0.000 (+)	0.000 (−)	0.000 (−)
F6	0.000 (−)	0.000 (−)	0.000 (−)	0.000 (−)	0.000 (−)	0.000 (−)	0.000 (−)	0.000 (−)	0.000 (−)
F7	0.000 (−)	0.000 (−)	0.000 (−)	0.000 (−)	0.023 (+)	0.000 (−)	0.001 (+)	0.000 (−)	0.000 (−)
F8	0.000 (−)	0.000 (−)	0.000 (−)	0.000 (−)	0.000 (+)	0.978 (=)	0.000 (+)	0.000 (−)	0.004 (−)
F9	0.000 (−)	0.000 (−)	0.000 (−)	0.000 (−)	0.000 (−)	0.000 (−)	0.000 (−)	0.000 (−)	0.000 (−)
F10	0.433 (=)	0.003 (+)	0.490 (=)	0.014 (−)	0.000 (−)	0.351 (=)	0.011 (−)	0.000 (−)	0.008 (−)
F11	0.000 (−)	0.000 (−)	0.000 (−)	0.000 (−)	0.000 (−)	0.000 (−)	0.000 (−)	0.000 (−)	0.000 (−)
F12	0.000 (−)	0.000 (−)	0.000 (−)	0.000 (−)	0.000 (−)	0.000 (−)	0.000 (−)	0.000 (−)	0.008 (+)
F13	0.000 (−)	0.000 (−)	0.000 (−)	0.000 (−)	0.000 (−)	0.000 (−)	0.000 (−)	0.000 (−)	0.000 (−)
F14	0.000 (−)	0.000 (−)	0.000 (−)	0.000 (−)	0.000 (−)	0.000 (−)	0.000 (−)	0.000 (−)	0.000 (−)
F15	0.000 (−)	0.000 (−)	0.000 (−)	0.000 (−)	0.000 (−)	0.000 (−)	0.000 (−)	0.000 (−)	0.000 (−)
F16	0.000 (−)	0.006 (−)	0.005 (−)	0.000 (−)	0.310 (=)	0.579 (=)	0.797 (=)	0.000 (−)	0.117 (=)
F17	0.000 (−)	0.000 (−)	0.000 (−)	0.000 (−)	0.000 (−)	0.829 (=)	0.110 (=)	0.000 (−)	0.000 (−)
F18	0.000 (−)	0.000 (−)	0.000 (−)	0.000 (−)	0.000 (−)	0.000 (−)	0.000 (−)	0.000 (−)	0.000 (−)
F19	0.000 (−)	0.000 (−)	0.000 (−)	0.000 (−)	0.000 (−)	0.000 (−)	0.000 (−)	0.000 (−)	0.000 (−)
F20	0.000 (−)	0.000 (−)	0.000 (−)	0.000 (−)	0.000 (−)	0.025 (−)	0.199 (=)	0.000 (−)	0.000 (−)
F21	0.000 (−)	0.000 (−)	0.000 (−)	0.000 (−)	0.008 (+)	0.171 (=)	0.000 (+)	0.000 (−)	0.003 (−)
F22	0.002 (−)	0.081 (=)	0.009 (−)	0.000 (−)	0.000 (−)	0.010 (−)	0.162 (=)	0.000 (−)	0.000 (−)
F23	0.000 (−)	0.000 (−)	0.000 (−)	0.000 (−)	0.000 (+)	0.665 (=)	0.001 (+)	0.000 (−)	0.001 (−)
F24	0.000 (−)	0.000 (−)	0.000 (−)	0.000 (−)	0.903 (−)	0.607 (=)	0.000 (+)	0.000 (−)	0.000 (−)
F25	0.001 (−)	0.001 (−)	0.106 (=)	0.000 (−)	0.000 (−)	0.000 (−)	0.204 (=)	0.000 (−)	1.000 (=)
F26	0.000 (−)	0.003 (−)	0.016 (−)	0.006 (−)	0.000 (+)	0.099 (=)	0.000 (+)	0.000 (−)	0.000 (−)
F27	0.000 (−)	0.000 (−)	0.000 (−)	0.000 (−)	0.000 (−)	0.000 (−)	0.001 (−)	0.001 (+)	0.037 (−)
F28	0.078 (=)	0.871 (=)	0.499 (=)	0.000 (−)	0.041 (−)	0.206 (=)	0.258 (=)	0.000 (−)	0.070 (=)
F29	0.000 (−)	0.000 (−)	0.000 (−)	0.000 (−)	0.000 (−)	0.000 (−)	0.030 (+)	0.000 (−)	0.000 (−)
F30	0.000 (−)	0.000 (−)	0.000 (−)	0.000 (−)	0.000 (−)	0.000 (−)	0.000 (−)	0.000 (−)	0.000 (−)
+/=/−	0/2/27	2/3/24	0/4/25	0/0/29	6/2/21	0/10/19	9/6/14	1/0/28	2/3/24

**Table 19:** Wilcoxon rank–sum test results for IGKSO and other algorithms on the CEC2019 test set.

	GKSO	IGKSO1	EGKSO	MGKSO	IAOA	MTV–SCA	MDBO	TTHHO	ALA
F1	NaN (=)	NaN (=)	NaN (=)	NaN (=)	0.000 (−)	0.000 (−)	NaN (=)	NaN (=)	0.000 (−)
F2	0.000 (−)	0.000 (−)	0.000 (−)	0.000 (−)	0.000 (−)	0.000 (−)	0.000 (−)	0.000 (−)	0.000 (−)
F3	0.000 (−)	0.164 (=)	0.225 (=)	0.000 (−)	0.000 (−)	0.000 (−)	0.667 (=)	0.000 (−)	0.000 (−)
F4	0.000 (−)	0.000 (−)	0.042 (−)	0.000 (−)	0.000 (+)	0.017 (−)	0.079 (=)	0.000 (−)	0.000 (−)
F5	0.000 (−)	0.001 (−)	0.177 (=)	0.000 (−)	0.000 (+)	0.001 (+)	0.164 (=)	0.000 (−)	0.000 (−)
F6	0.000 (−)	0.000 (−)	0.040 (−)	0.000 (−)	0.000 (−)	0.004 (−)	0.342 (=)	0.000 (−)	0.000 (−)
F7	0.000 (−)	0.000 (−)	0.000 (−)	0.000 (−)	0.000 (−)	0.000 (−)	0.000 (−)	0.000 (−)	0.000 (−)
F8	0.000 (−)	0.006 (+)	0.068 (−)	0.000 (−)	0.023 (−)	0.000 (−)	0.152 (=)	0.000 (−)	0.000 (−)
F9	0.000 (−)	0.003 (−)	0.438 (=)	0.000 (−)	0.663 (=)	0.000 (−)	0.000 (−)	0.000 (−)	0.000 (−)
F10	0.372 (=)	0.162 (=)	0.001 (−)	0.000 (−)	0.012 (−)	0.000 (−)	0.014 (−)	0.001 (−)	0.000 (−)
+/=/−	0/2/8	1/3/6	0/4/6	0/1/9	2/1/7	1/0/9	0/6/4	0/1/9	0/0/10

**Table 20:** Friedman test results on the 30-dimensional CEC2017 test set.

	IGKSO	GKSO	IGKSO1	EGKSO	MGKSO	IAOA	MTV-SCA	MDBO	TTHHO	ALA
Avg.rank	2.10	7.76	6.41	5.03	7.90	5.45	4.06	2.59	9.66	4.03
sort	1	8	7	5	9	6	4	2	10	3

**Table 21:** Friedman test results on the CEC2019 test set.

	IGKSO	GKSO	IGKSO1	EGKSO	MGKSO	IAOA	MTV-SCA	MDBO	TTHHO	ALA
Avg.rank	1.80	5.50	4.20	3.60	7.00	5.50	6.20	2.70	8.50	10.00
sort	1	5	4	3	8	6	7	2	9	10

**Table 22:** Nemenyi post hoc test results on the 30-dimensional CEC2017 test set.

	IGKSO	GKSO	IGKSO1	EGKSO	MGKSO	IAOA	MTV-SCA	MDBO	TTHHO	ALA
**IGKSO**	–	0.0000	0.0000	0.0002	0.0000	0.0000	0.0041	0.0414	0.0000	0.0043
**GKSO**	0.0000	–	0.0111	0.0066	0.0668	0.0022	0.0000	0.0000	0.0045	0.0000
**IGKSO** **1**	0.0000	0.0111	–	0.0106	0.0089	0.0206	0.0021	0.0000	0.0000	0.0020
**E** **GKSO**	0.0002	0.0066	0.0106	–	0.0003	0.0457	0.0203	0.0018	0.0000	0.0194
**M** **GKSO**	0.0000	**0.0668**	0.0089	0.0003	–	0.0017	0.0000	0.0000	0.0057	0.0000
**IAOA**	0.0000	0.0022	0.0206	0.0457	0.0017	–	0.0104	0.0003	0.0000	0.0100
**MTV–SCA**	0.0041	0.0000	0.0021	0.0203	0.0000	0.0104	–	0.0092	0.0000	0.0771
**MDBO**	0.0414	0.0000	0.0000	0.0018	0.0000	0.0003	0.0092	–	0.0000	0.0096
**TTHHO**	0.0000	0.0045	0.0000	0.0000	0.0057	0.0000	0.0000	0.0000	–	0.0000
**ALA**	0.0043	0.0000	0.0020	0.0194	0.0000	0.0100	**0.0771**	0.0096	0.0000	–

**Table 23:** Nemenyi post hoc test results on the CEC2019 test set.

	IGKSO	GKSO	IGKSO1	EGKSO	MGKSO	IAOA	MTV-SCA	MDBO	TTHHO	ALA
**IGKSO**	–	0.0000	0.0015	0.0061	0.0000	0.0040	0.0153	**0.0548**	0.0000	0.0157
**GKSO**	0.0000	–	0.0266	0.0074	**0.0721**	0.0110	0.0028	0.0001	0.0162	0.0027
**IGKSO** **1**	0.0015	0.0266	–	0.0259	0.0235	0.0372	0.0106	0.0025	0.0044	0.0103
**E** **GKSO**	0.0061	0.0074	0.0259	–	0.0064	**0.0580**	0.0369	0.0097	0.0006	0.0360
**M** **GKSO**	0.0000	**0.0721**	0.0235	0.0064	–	0.0097	0.0024	0.0001	0.0184	0.0024
**IAOA**	0.0040	0.0110	0.0372	**0.0580**	0.0097	–	0.0256	0.0065	0.0017	0.0250
**MTV–SCA**	0.0153	0.0028	0.0106	0.0369	0.0024	0.0256	–	0.0239	0.0000	**0.0783**
**MDBO**	**0.0548**	0.0001	0.0025	0.0097	0.0001	0.0065	0.0239	–	0.0000	0.0245
**TTHHO**	0.0000	0.0162	0.0044	0.0006	0.0184	0.0017	0.0000	0.0000	–	0.0000
**ALA**	0.0157	0.0027	0.0103	0.0360	0.0024	0.0250	**0.0783**	0.0245	0.0000	–

**Table 24:** The parameter configurations of the comparative algorithms.

Algorithm	Related Parameters
JADE	*P* = 0.1; *C* = 0.1;
KLDE	*LR* = 0.2; *EP* = 10; *F* = 0.5; *CR* = 0.9;
KLPSO	LR = 0.2; EP = 10; maxW = 0.9; minW = 0.4; C1 = 0.2; C2 = 0.2;

**Table 25:** RMSE on IGKSO and other comparative algorithms.

		IGKSO	JADE	KLDE	KLPSO	GWO
RMSE	mean std	**9.86 × 10^−4^** **2.28 × 10^−17^**	1.02 × 10^−3^ 5.36 × 10^−5^	1.07 × 10^−3^ 9.04 × 10^−5^	2.27 × 10^−3^ 5.52 × 10^−4^	2.27 × 10^−1^ 4.80 × 10^−2^

## Data Availability

The original contributions presented in this study are included in the article. Further inquiries can be directed to the corresponding author.
